# Supplementary material for: BCAS2 promotes primitive hematopoiesis by sequestering β-catenin within the nucleus
Source: eLife. 2025 Jun 13;13:RP100497. doi: 10.7554/eLife.100497 (PMC12165693; doi:10.7554/eLife.100497)

Fig S2C

Uncropped gels for Supplemental Figure2C T7 endonuclease

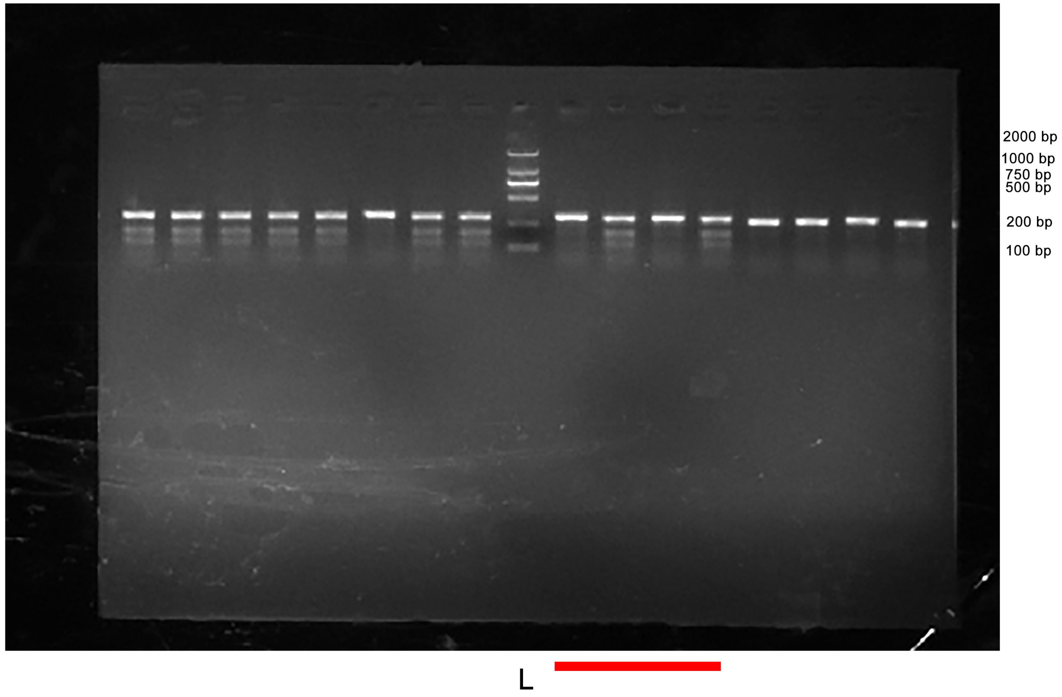

Uncropped gels for Supplemental Figure2C FspI

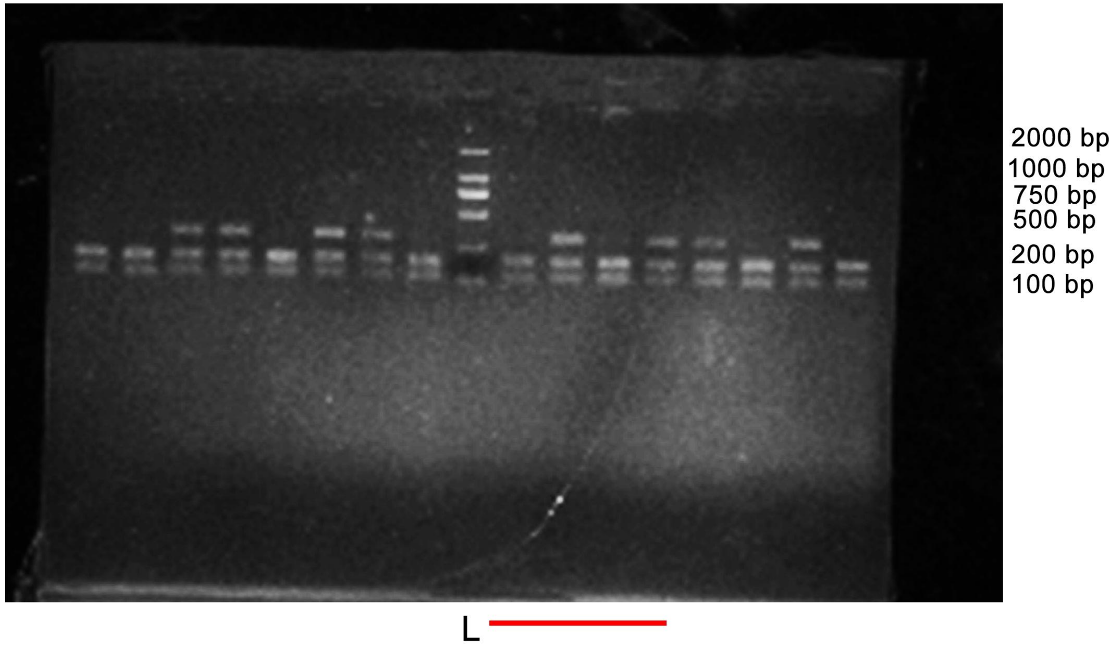

Supplement: Figure 1—figure supplement 2—source data 1. [file elife-100497-fig1-figsupp2-data1.zip › Figure 1—figure supplement 2—source data 1.pdf]
