# Supplementary figures and images for: BCAS2 promotes primitive hematopoiesis by sequestering β-catenin within the nucleus
Source: eLife. 2025 Jun 13;13:RP100497. doi: 10.7554/eLife.100497 (PMC12165693; doi:10.7554/eLife.100497)

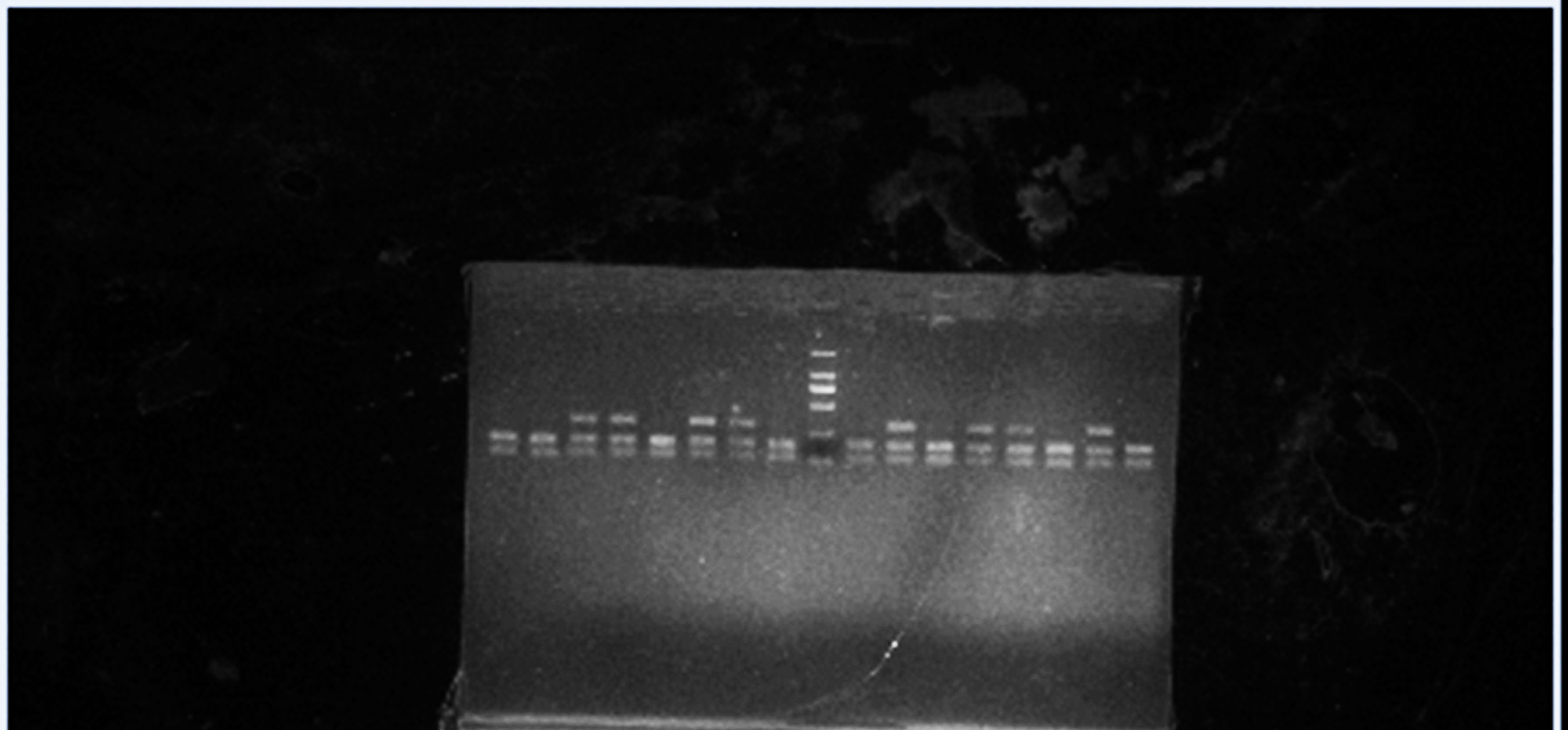

Supplement: Figure 1—figure supplement 2—source data 2. [file elife-100497-fig1-figsupp2-data2.zip › Figure 1—figure supplement 2/Fsip.jpg]

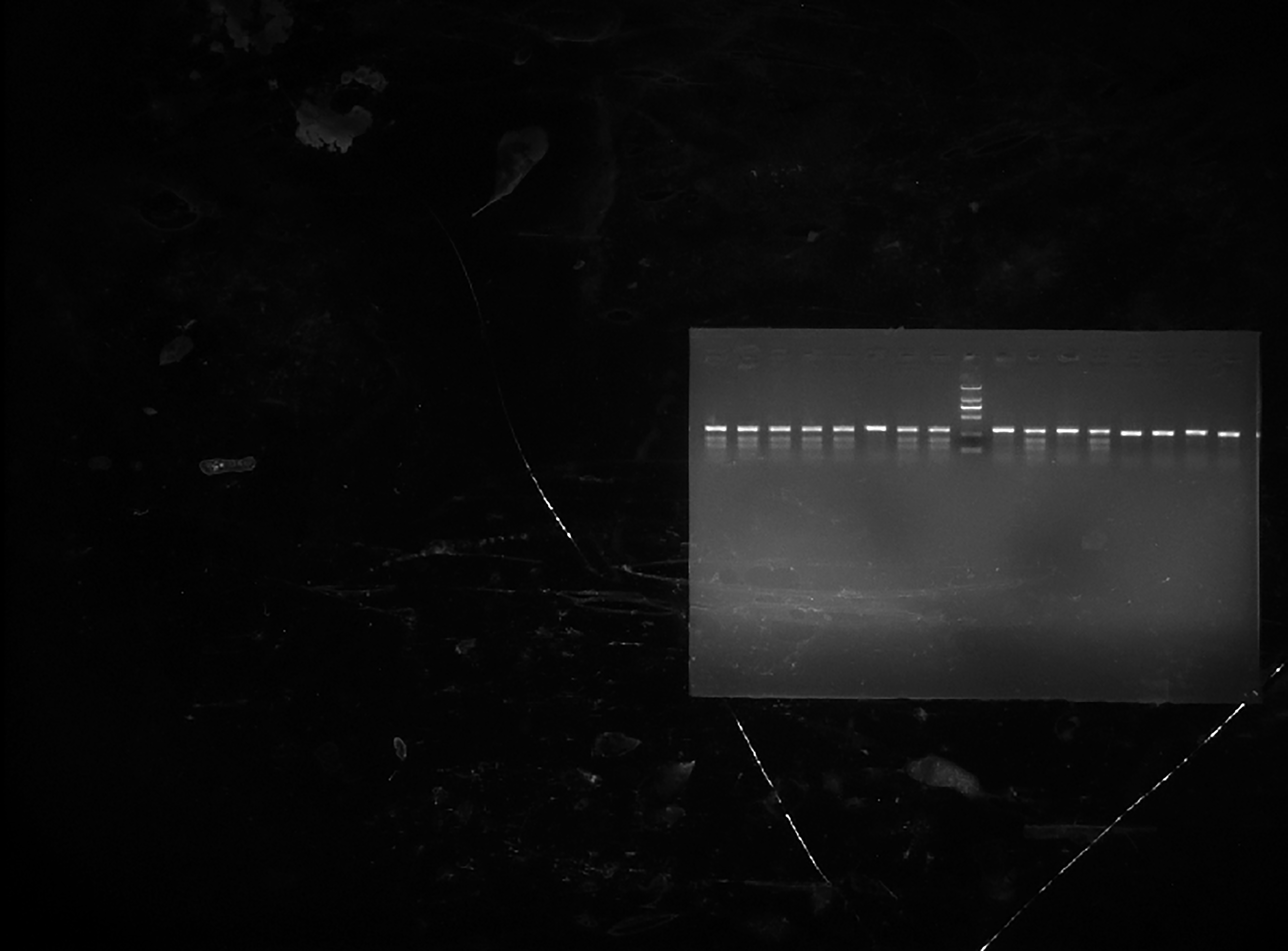

Supplement: Figure 1—figure supplement 2—source data 2. [file elife-100497-fig1-figsupp2-data2.zip › Figure 1—figure supplement 2/T7E1.jpg]

Figure 1-figure supplement 4

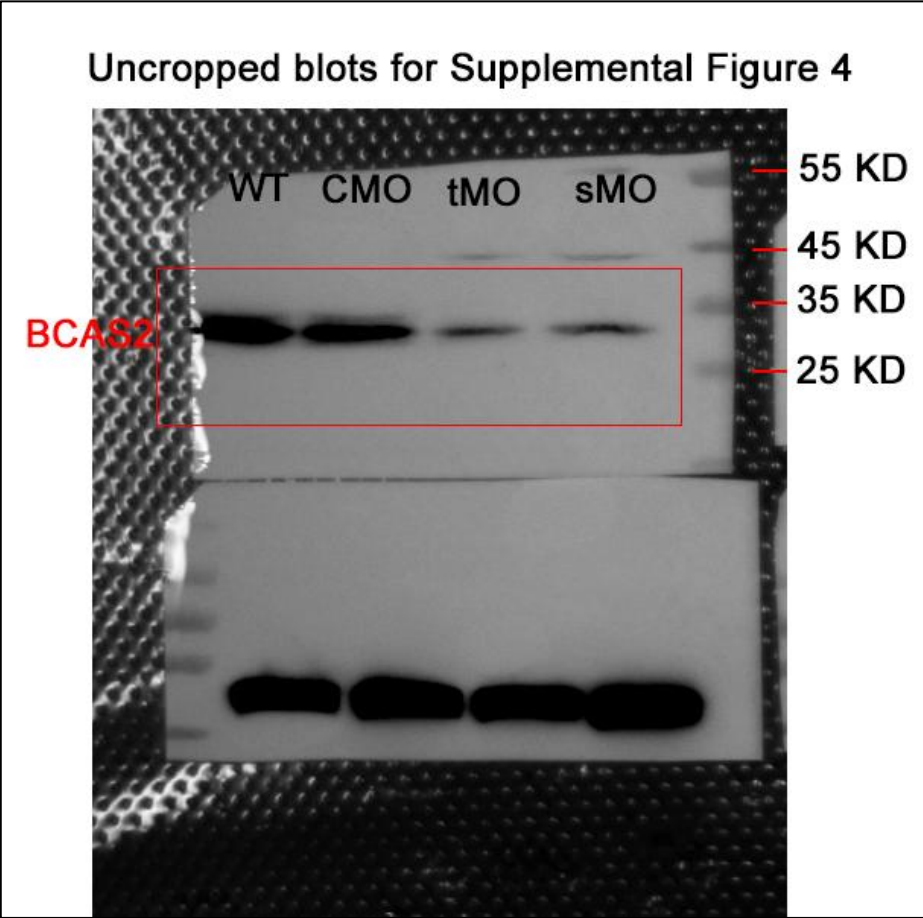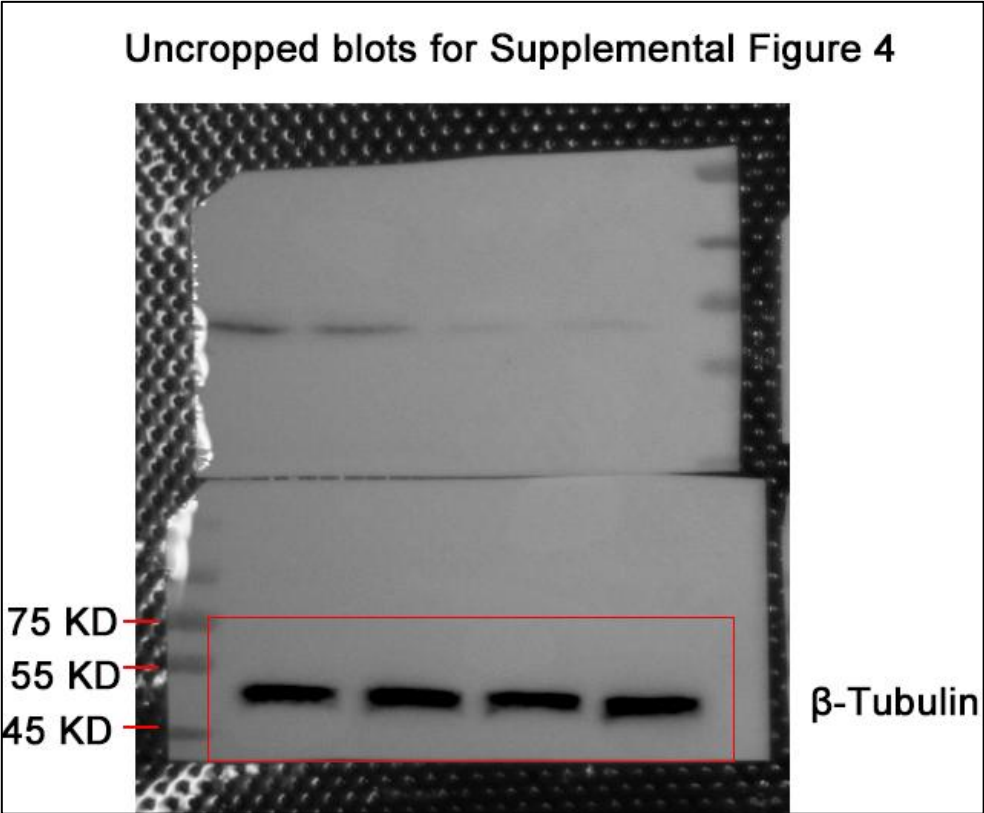

Supplement: Figure 1—figure supplement 4—source data 1. [file elife-100497-fig1-figsupp4-data1.zip › Figure 1-figure supplement 4-source data 1.pdf]

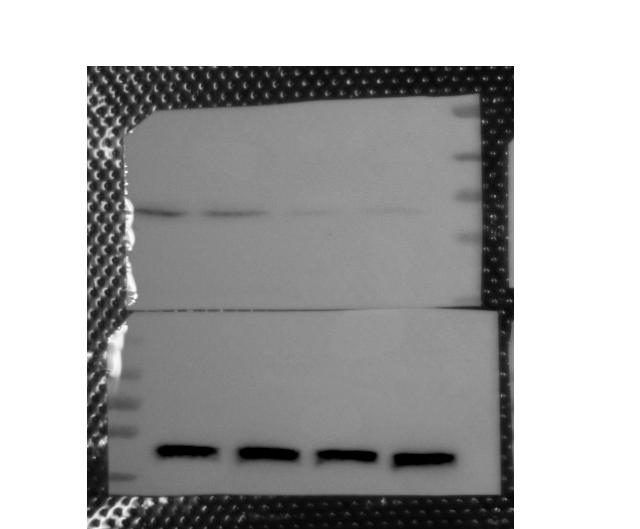

Supplement: Figure 1—figure supplement 4—source data 2. [file elife-100497-fig1-figsupp4-data2.zip › Figure 1-figure supplement 4-source data 2/b-TUBULIN.tif]

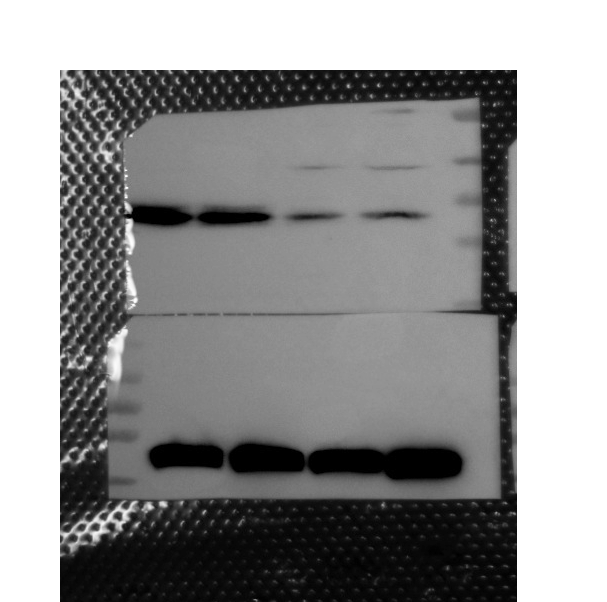

Supplement: Figure 1—figure supplement 4—source data 2. [file elife-100497-fig1-figsupp4-data2.zip › Figure 1-figure supplement 4-source data 2/BCAS2.tif]

Figure 3D

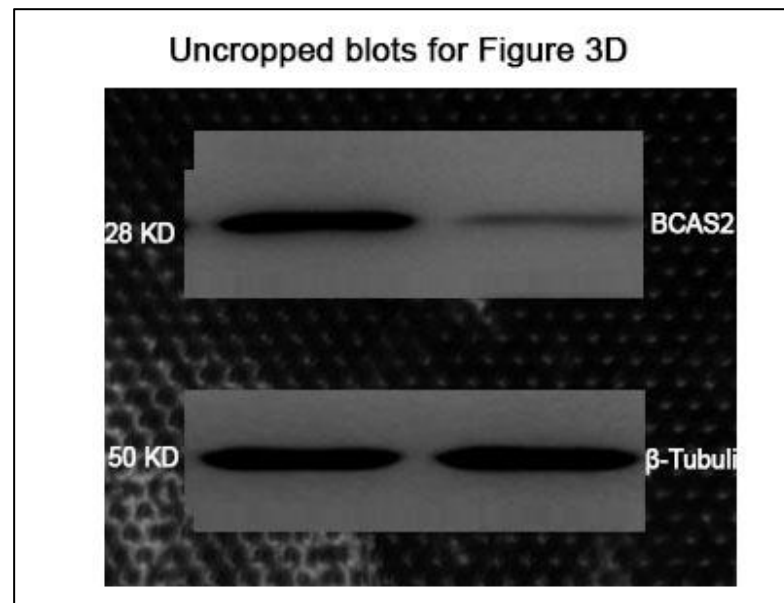

Supplement: Figure 3—source data 1. [file elife-100497-fig3-data1.zip › Figure 3-source data 1.pdf]

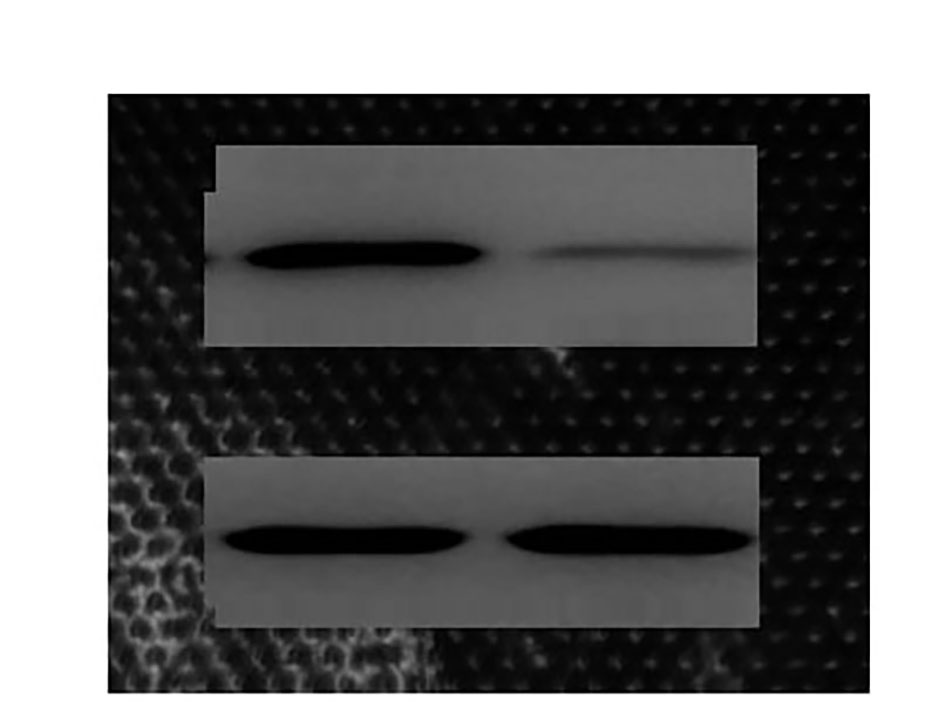

Supplement: Figure 3—source data 2. [file elife-100497-fig3-data2.zip › Figure 3-source data 2/Figure 3-source data 2.tif]

Figure 3-figure supplement 1

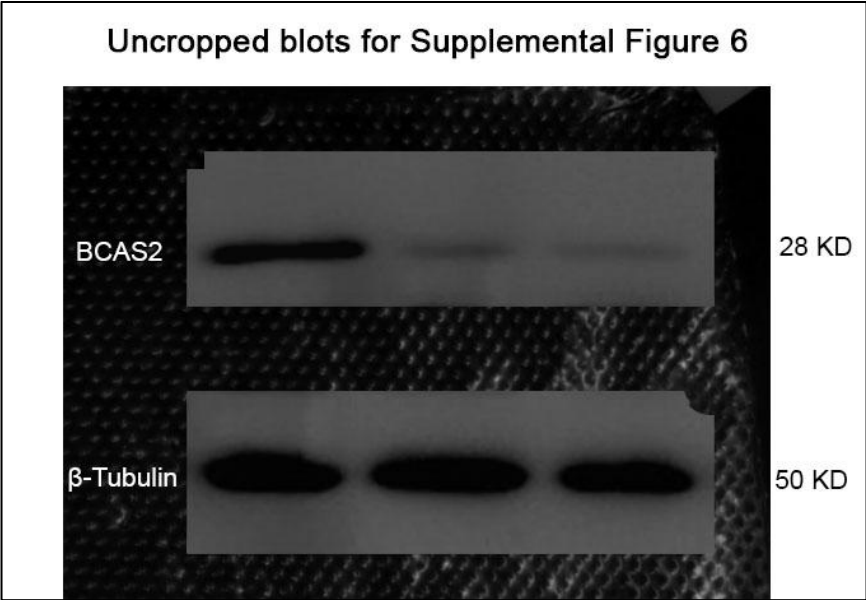

Supplement: Figure 3—figure supplement 1—source data 1. [file elife-100497-fig3-figsupp1-data1.zip › Figure 3-figure supplement 1-source data 1.pdf]

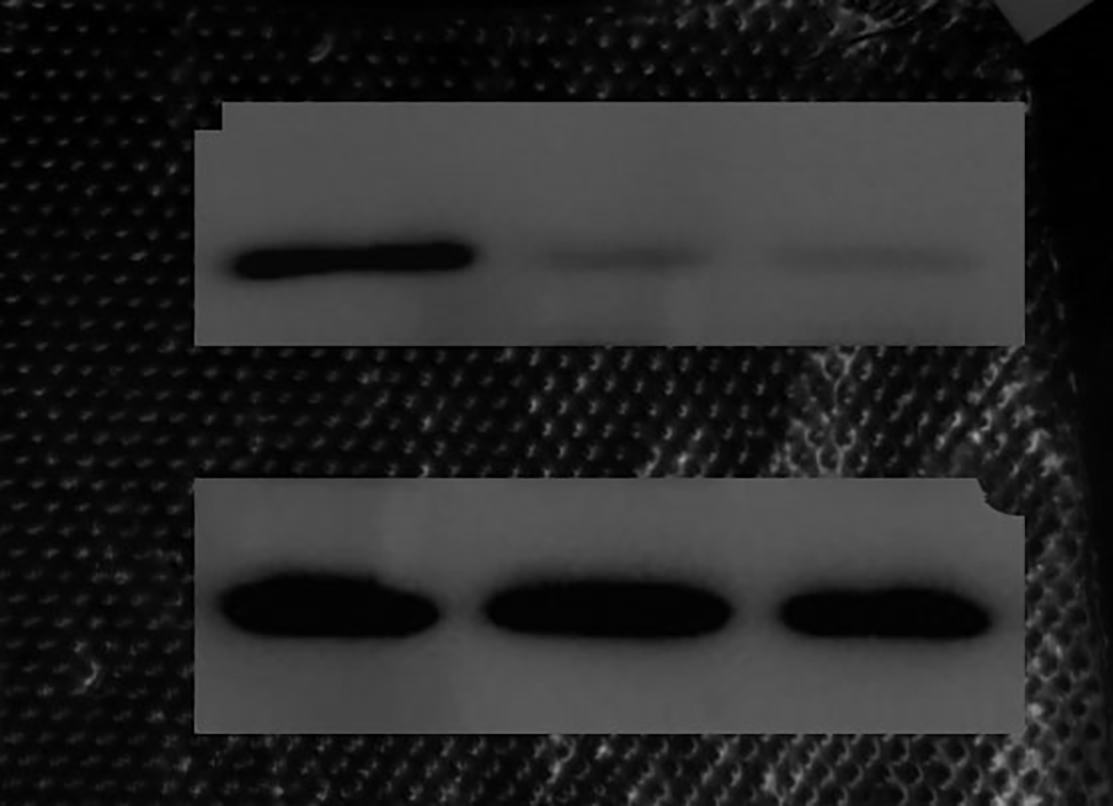

Supplement: Figure 3—figure supplement 1—source data 2. [file elife-100497-fig3-figsupp1-data2.zip › Figure 3-figure supplement 1-source data 2/Figure 3-figure supplement 1-source data 2.tif]

Figure 4E

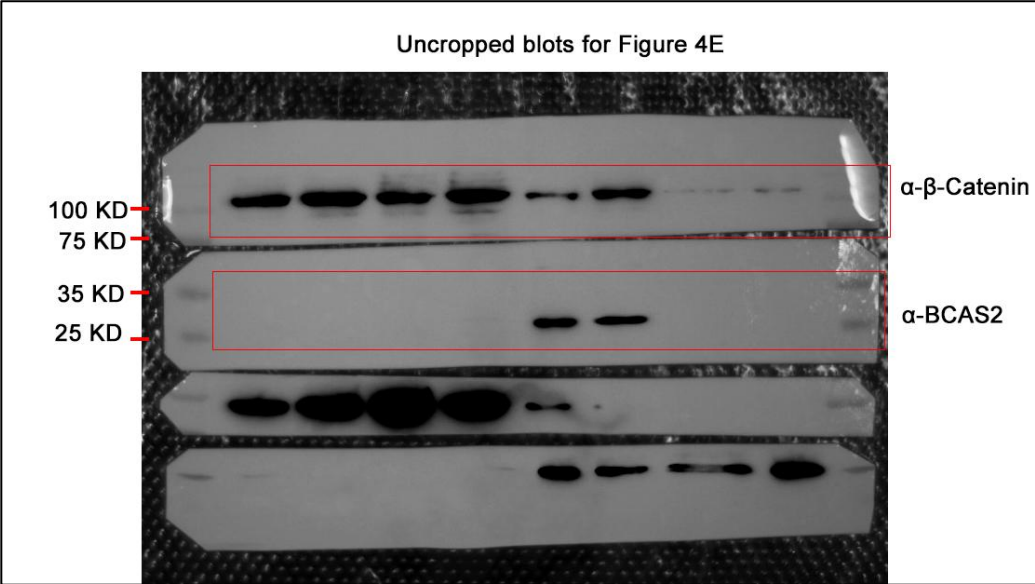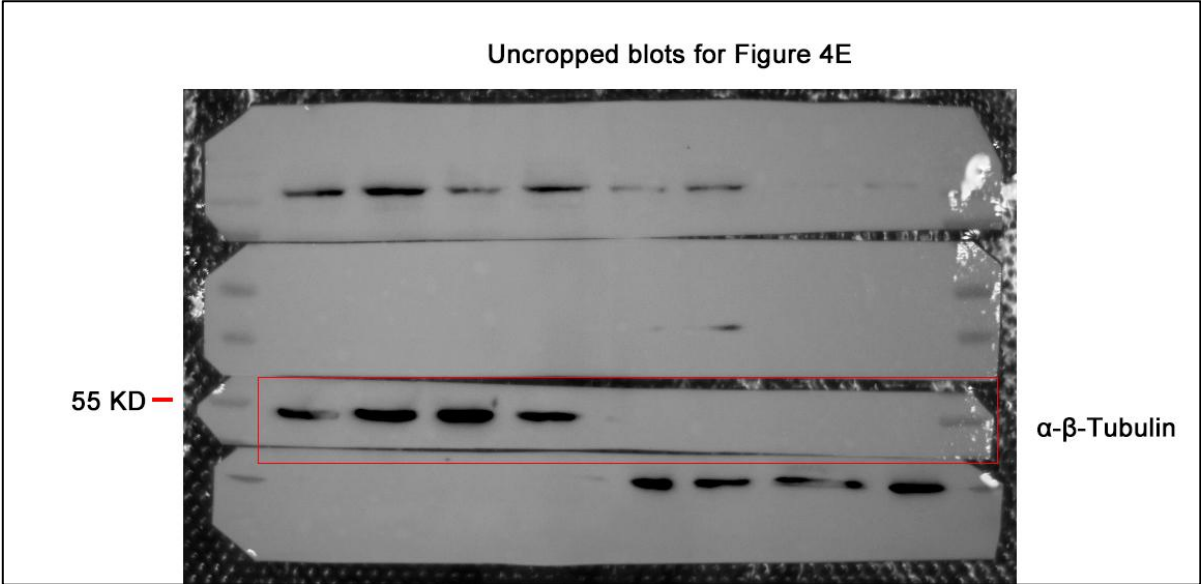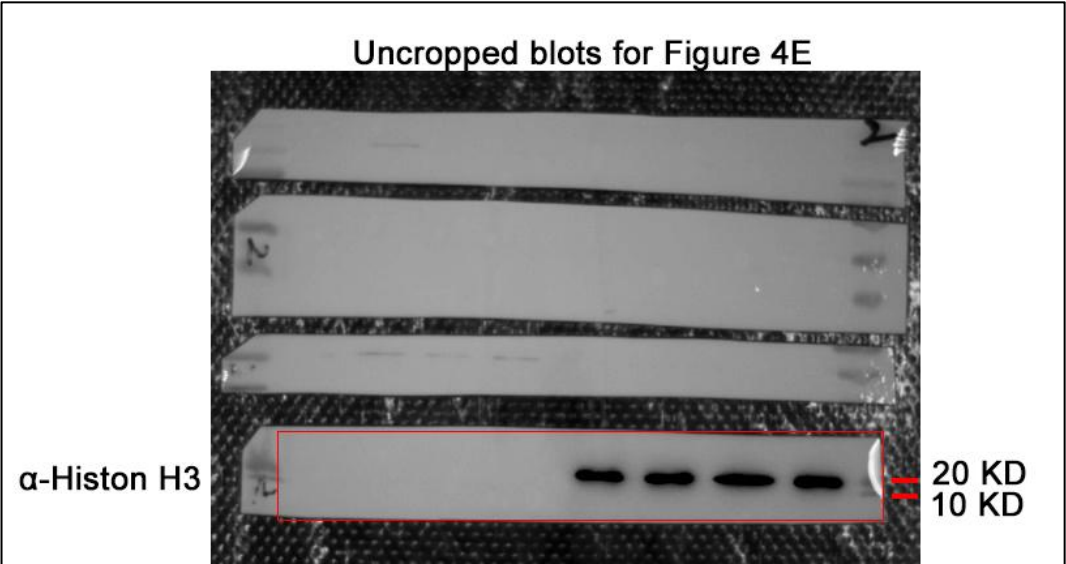

Supplement: Figure 4—source data 1. [file elife-100497-fig4-data1.zip › Figure 4-source data 1.pdf]

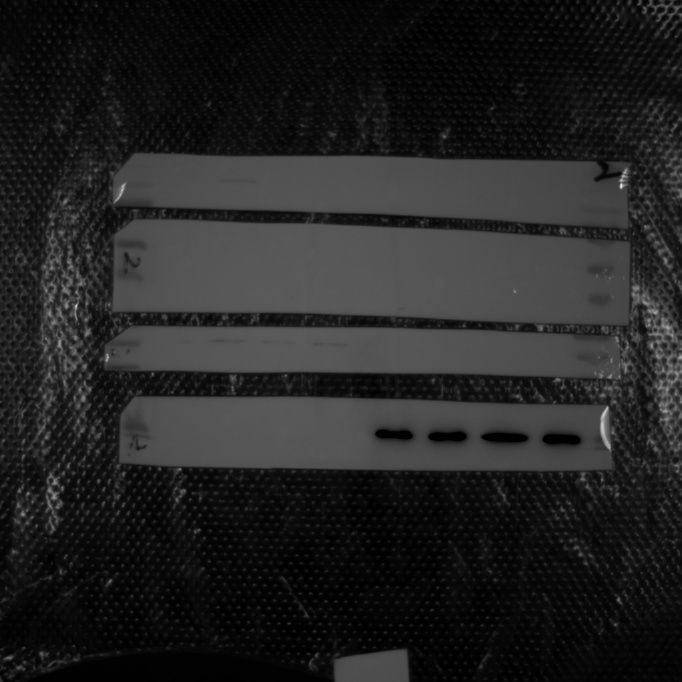

Supplement: Figure 4—source data 2. [file elife-100497-fig4-data2.zip › Figure 4-source data 2/Figure 4-source data 2 (1).jpg]

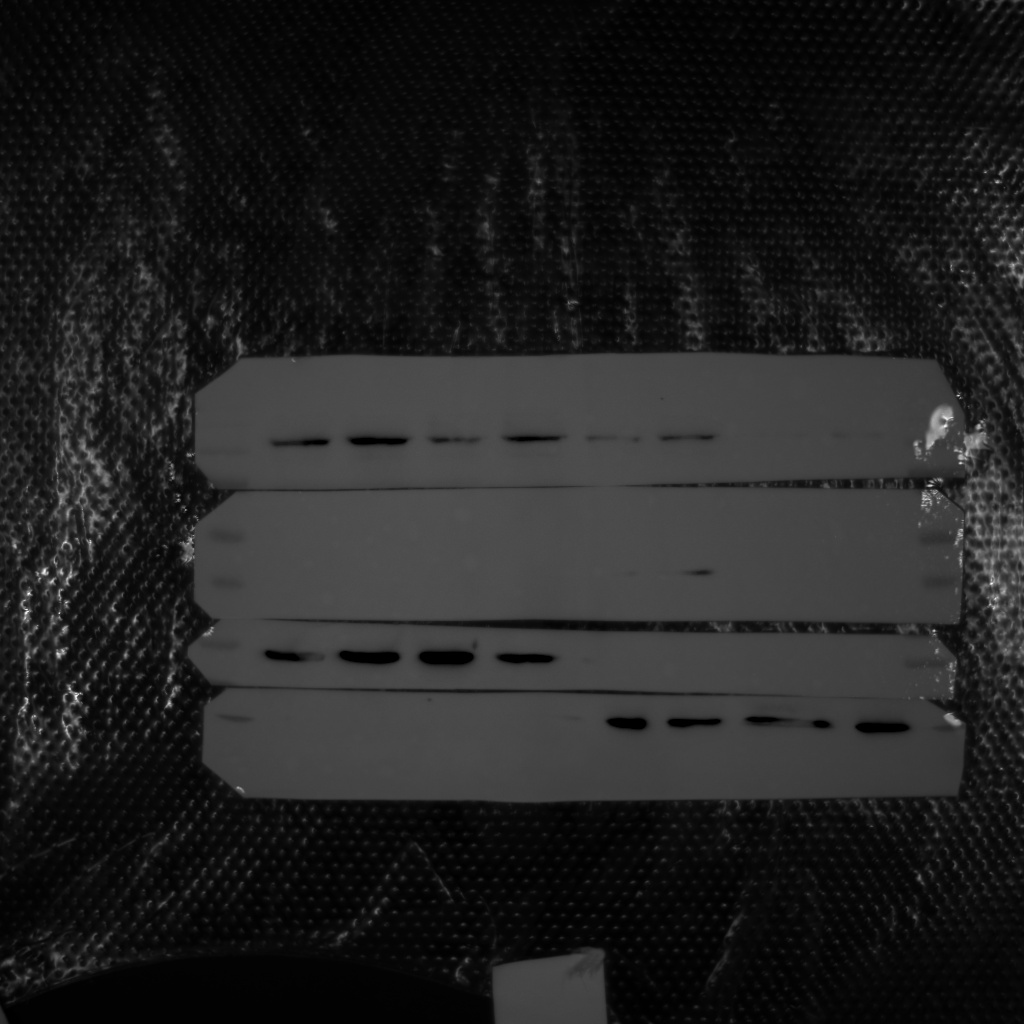

Supplement: Figure 4—source data 2. [file elife-100497-fig4-data2.zip › Figure 4-source data 2/Figure 4-source data 2 (2).jpg]

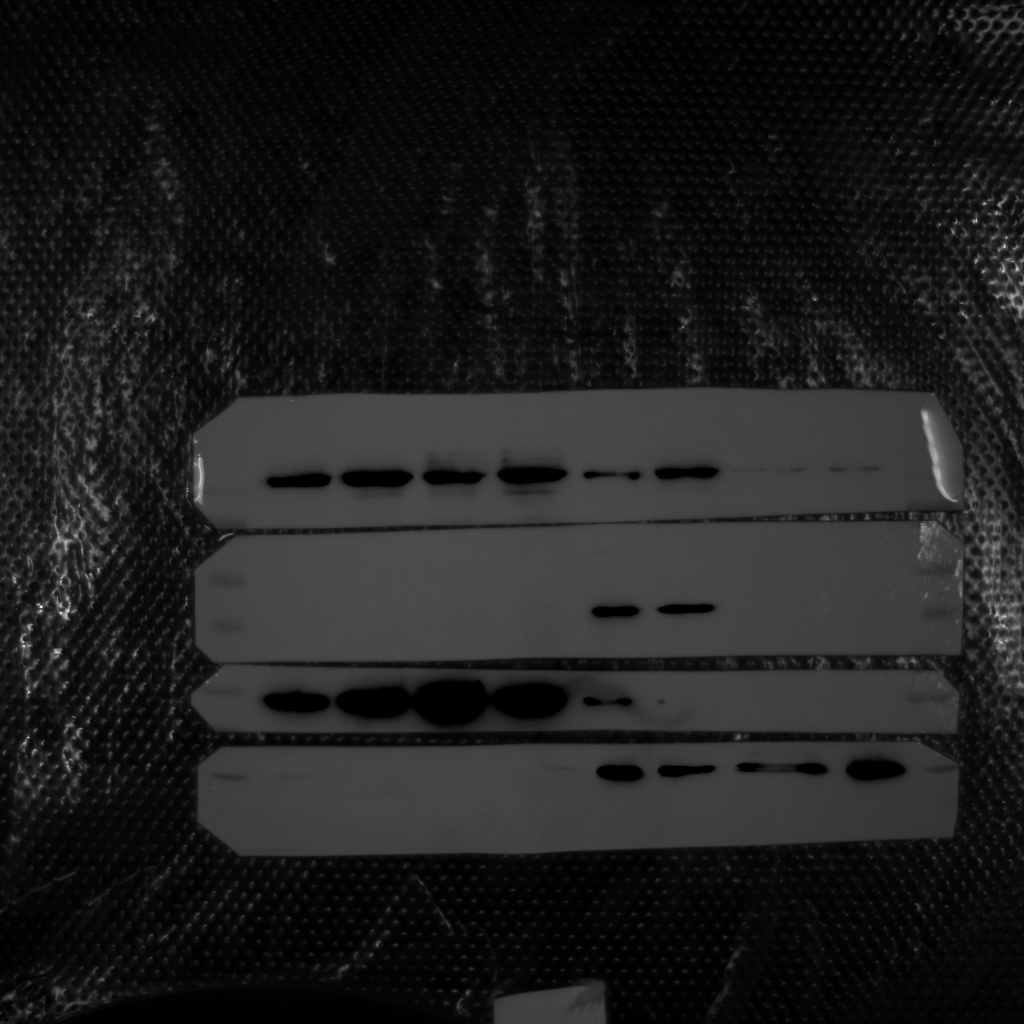

Supplement: Figure 4—source data 2. [file elife-100497-fig4-data2.zip › Figure 4-source data 2/Figure 4-source data 2.jpg]

Figure 6A

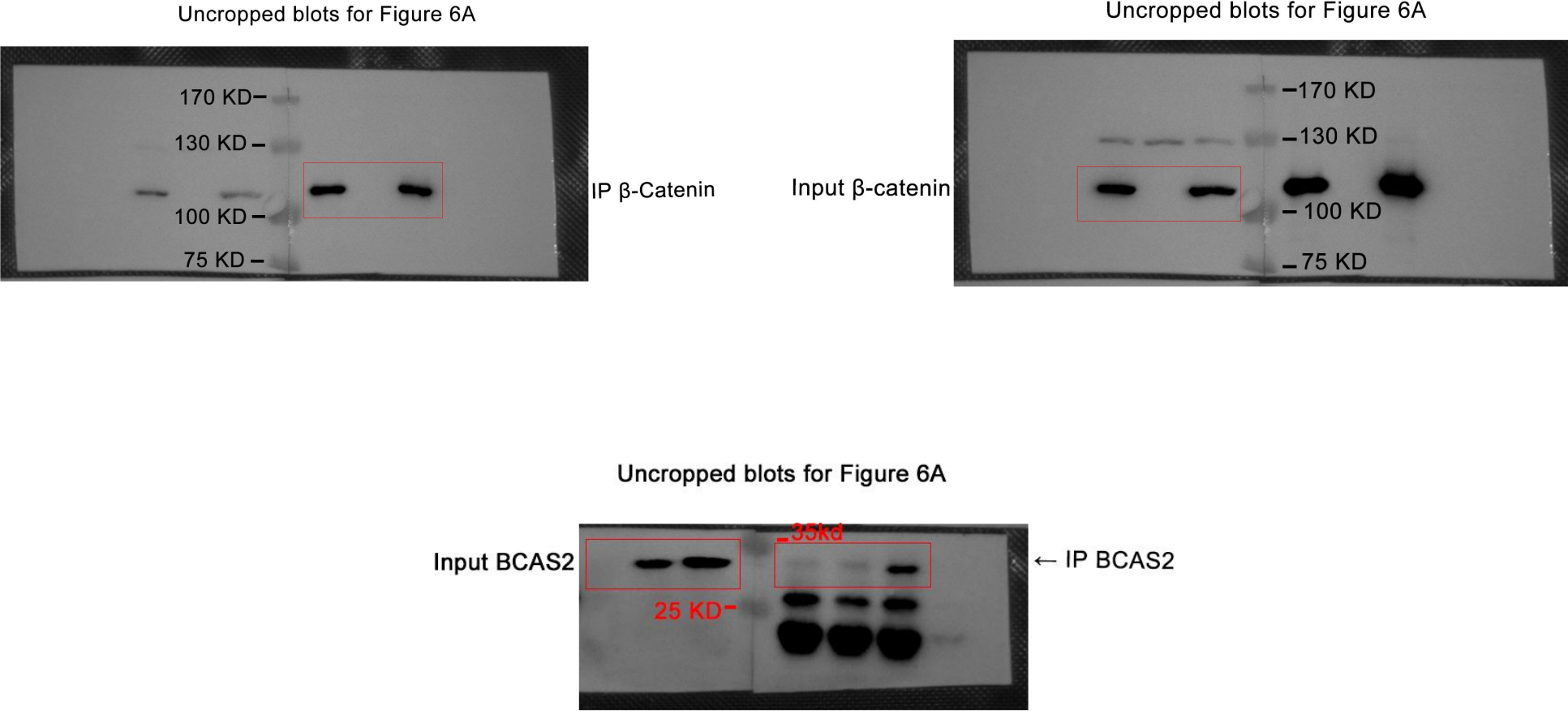

Figure 6B

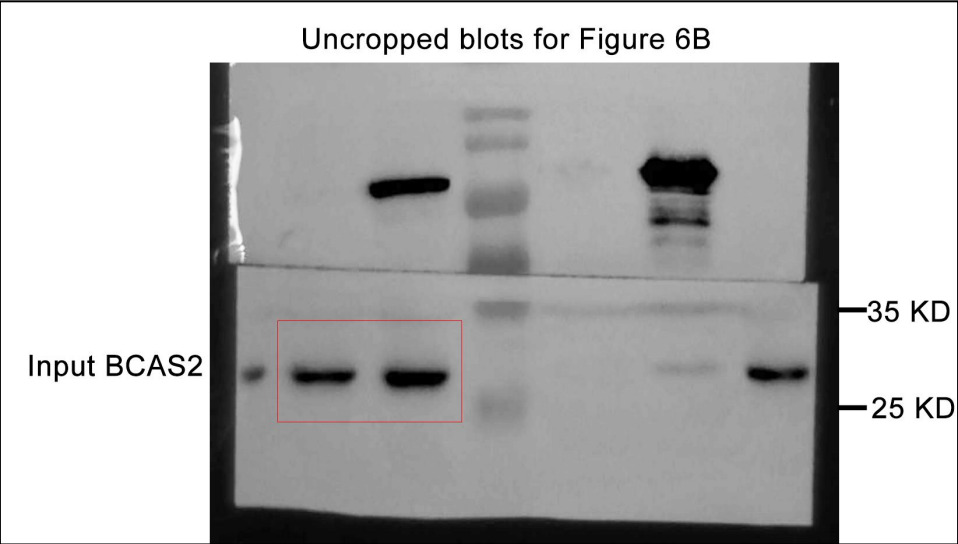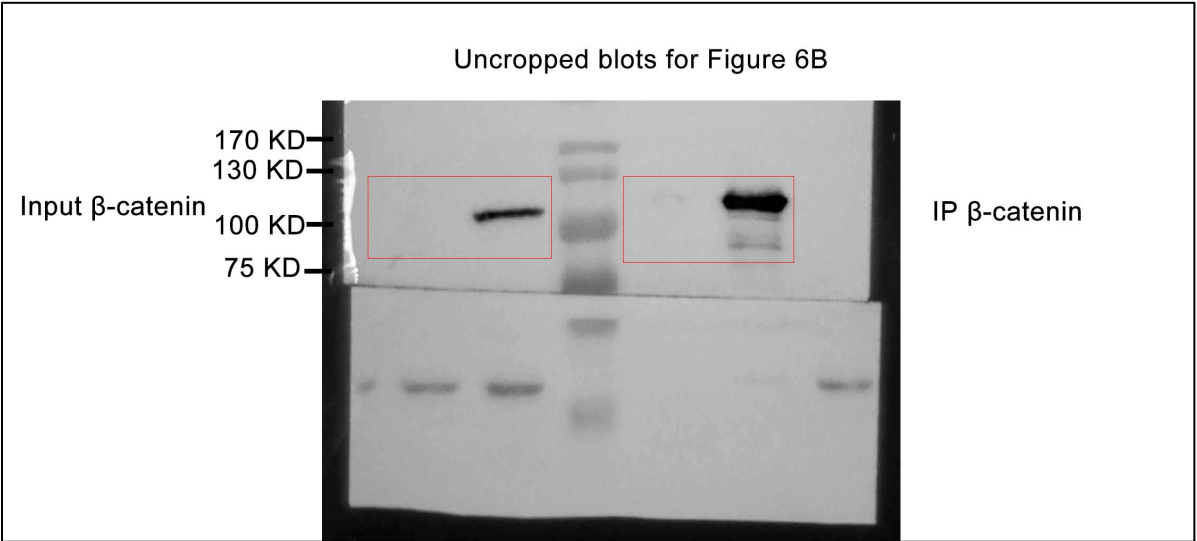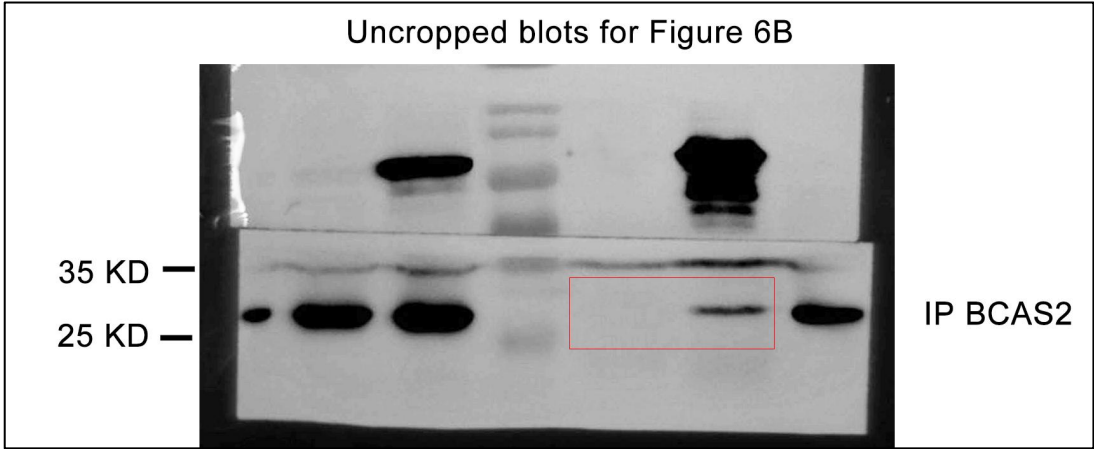

Figure 6C

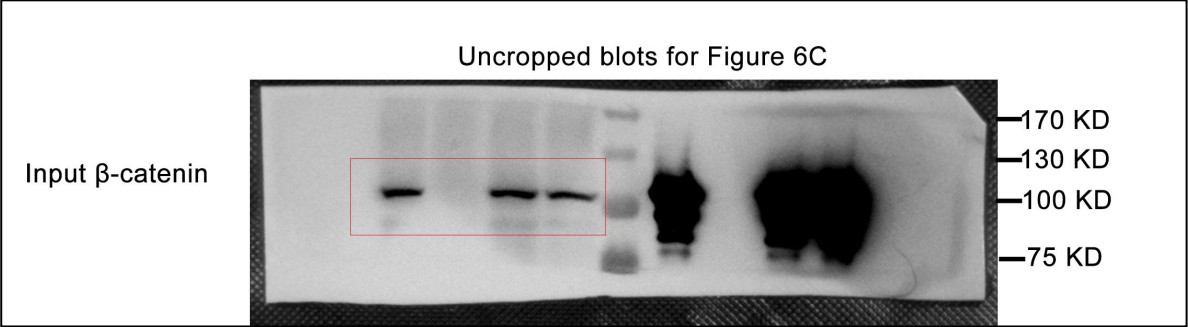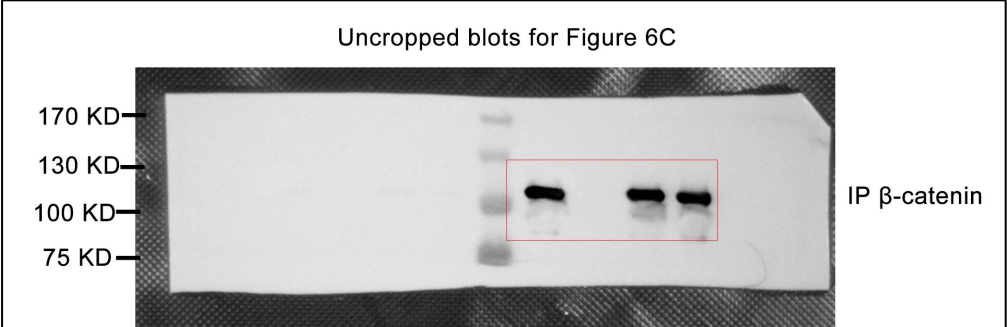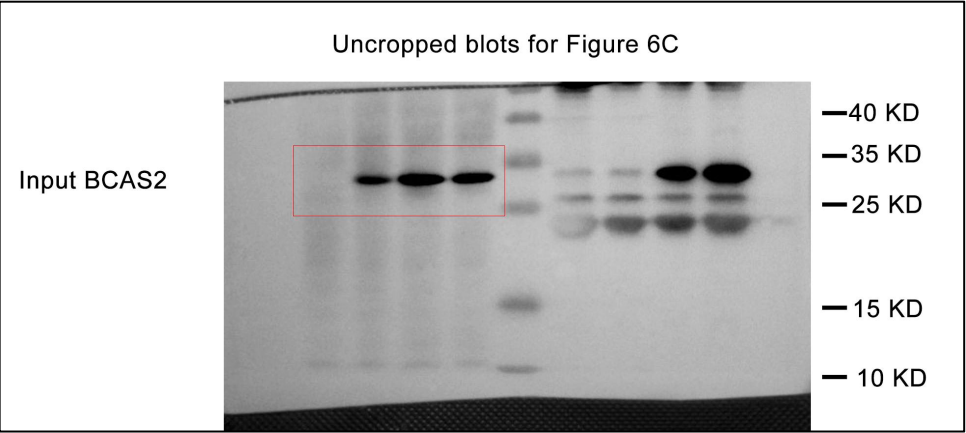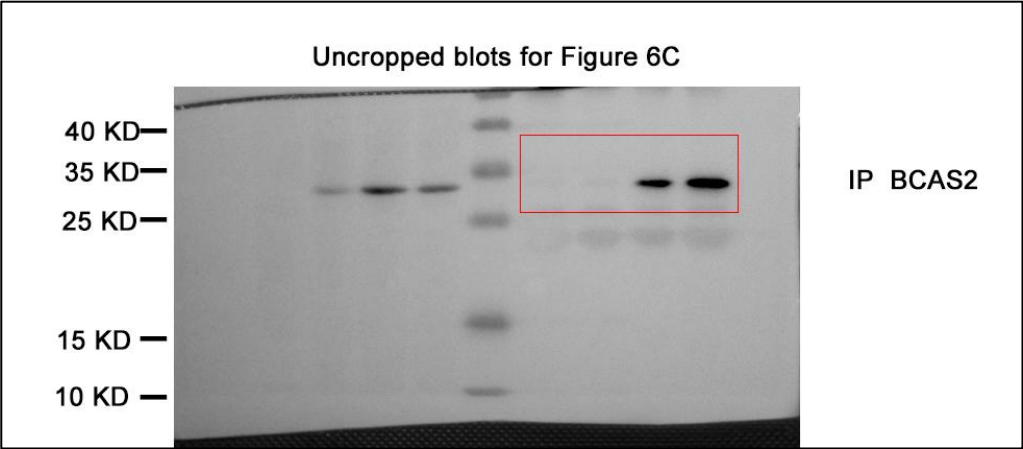

Figure 6G

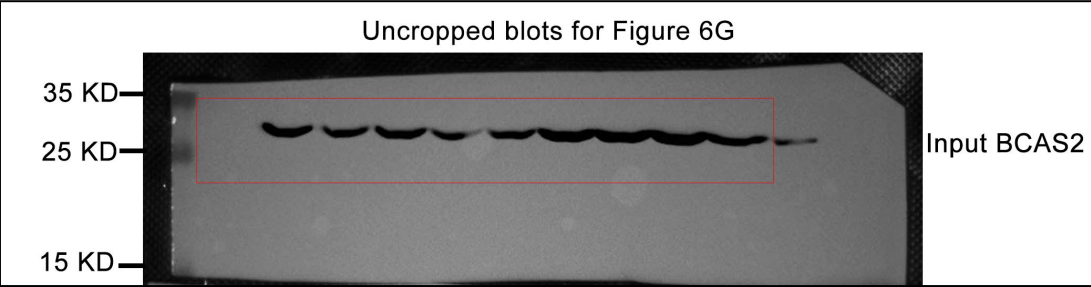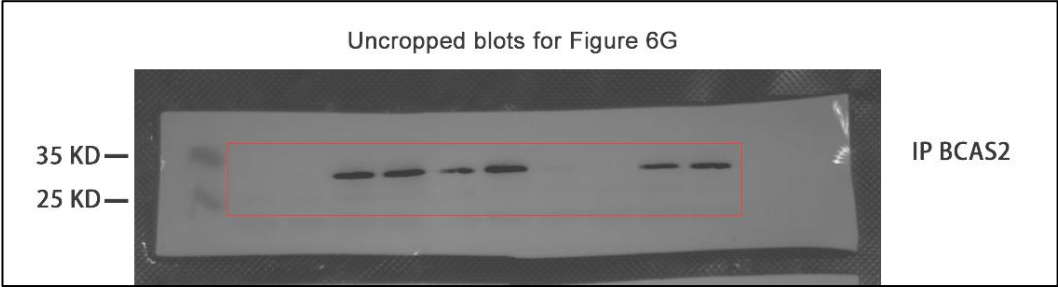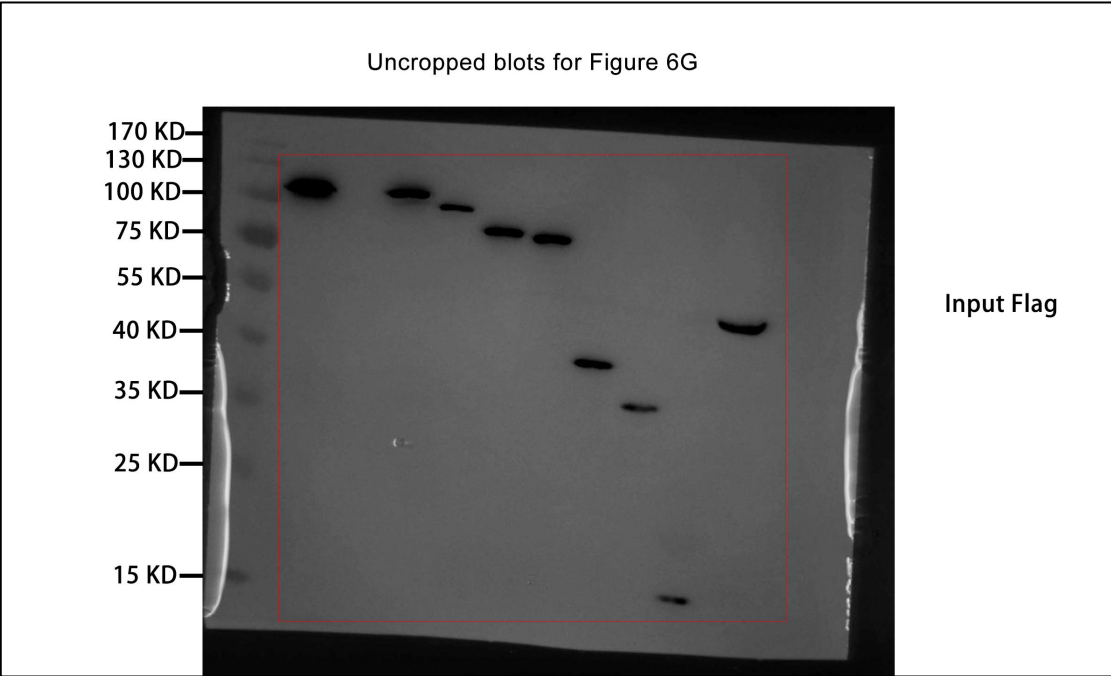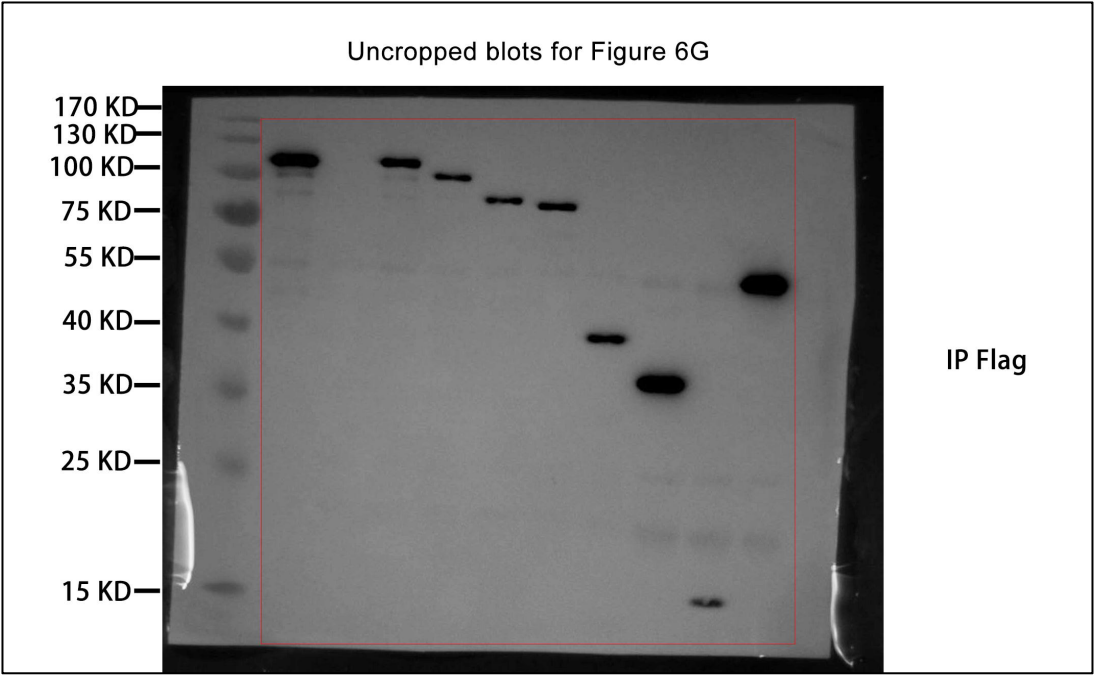

Figure 6H

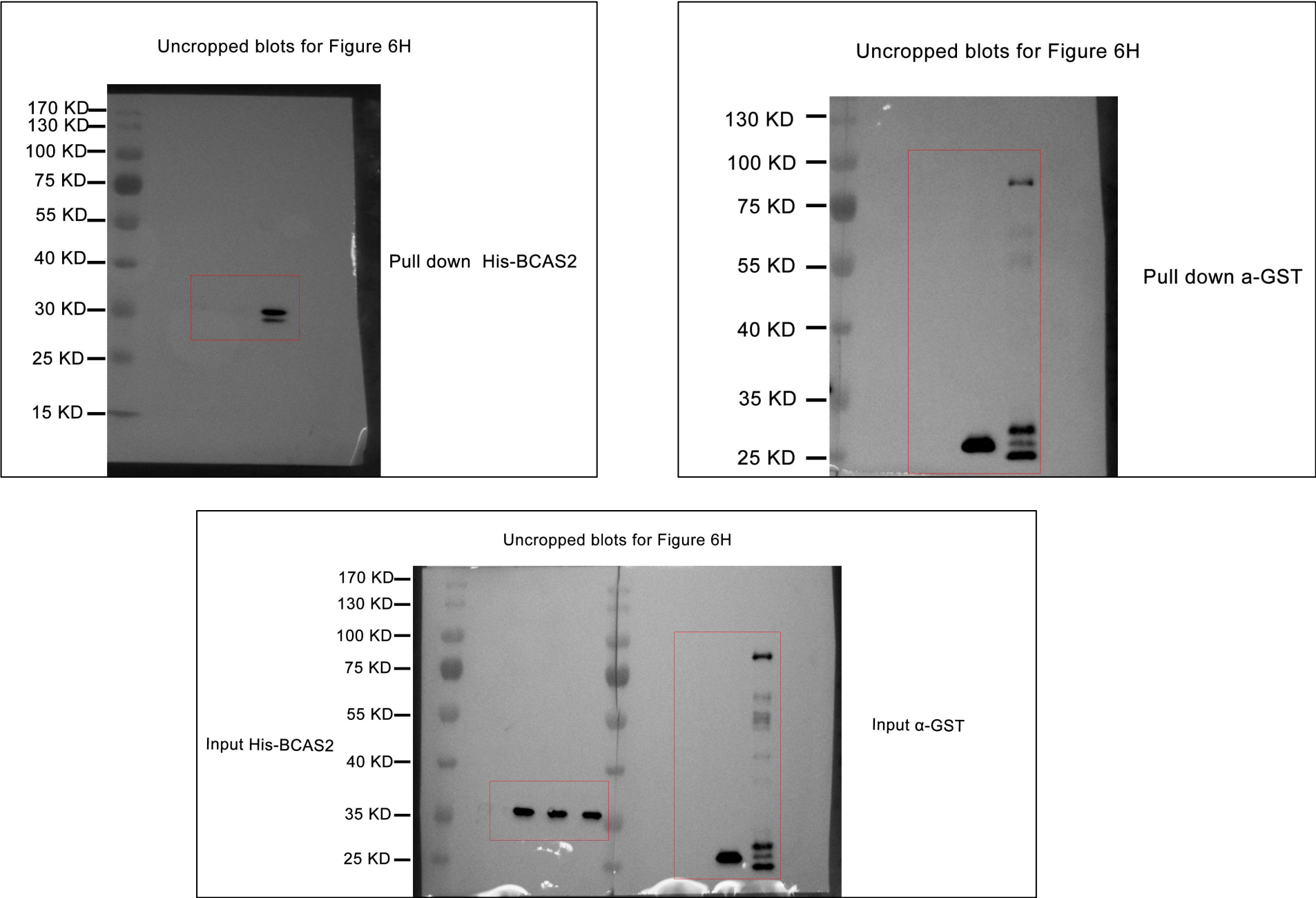

Supplement: Figure 6—source data 1. [file elife-100497-fig6-data1.zip › Figure 6-source data 1.pdf]

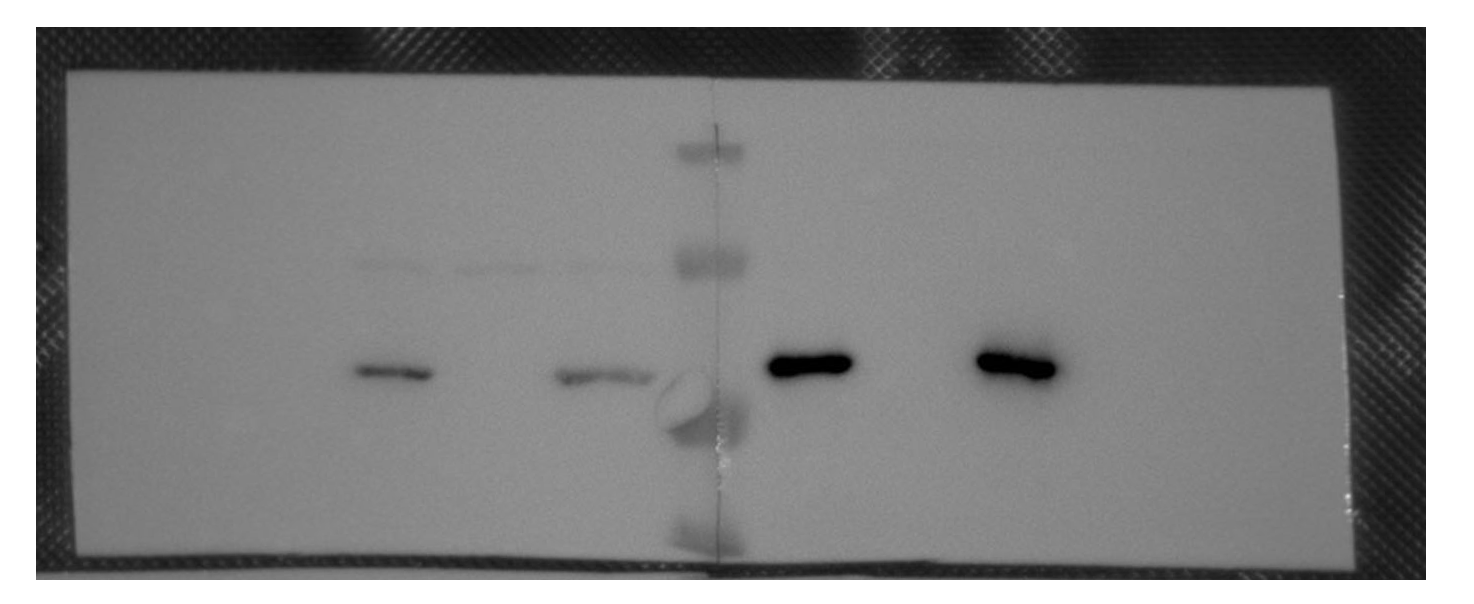

Supplement: Figure 6—source data 2. [file elife-100497-fig6-data2.zip › Figure 6-source data 2/Figure 6A-source data 2/Flag b cat ip.tif]

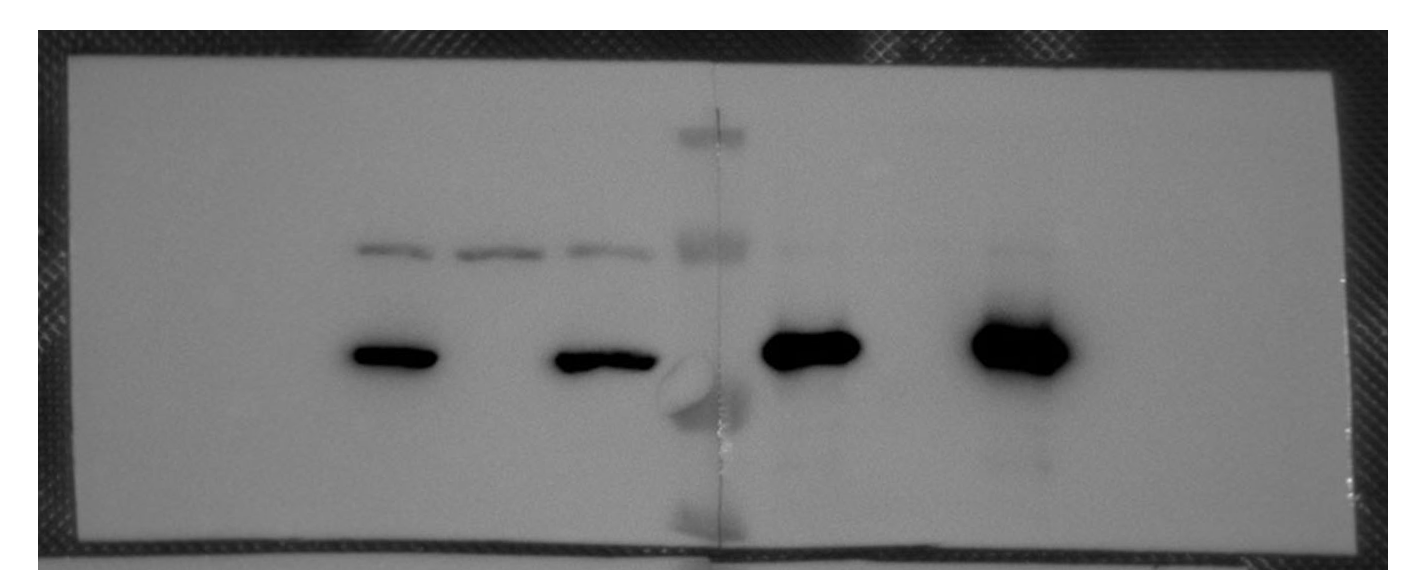

Supplement: Figure 6—source data 2. [file elife-100497-fig6-data2.zip › Figure 6-source data 2/Figure 6A-source data 2/Flag b cat input.tif]

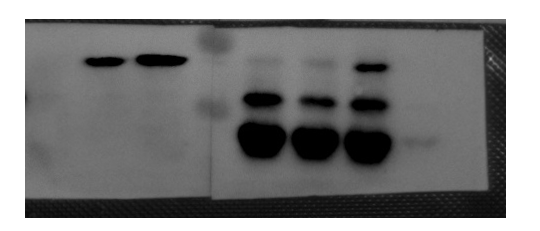

Supplement: Figure 6—source data 2. [file elife-100497-fig6-data2.zip › Figure 6-source data 2/Figure 6A-source data 2/HA bcas2 ip.tif]

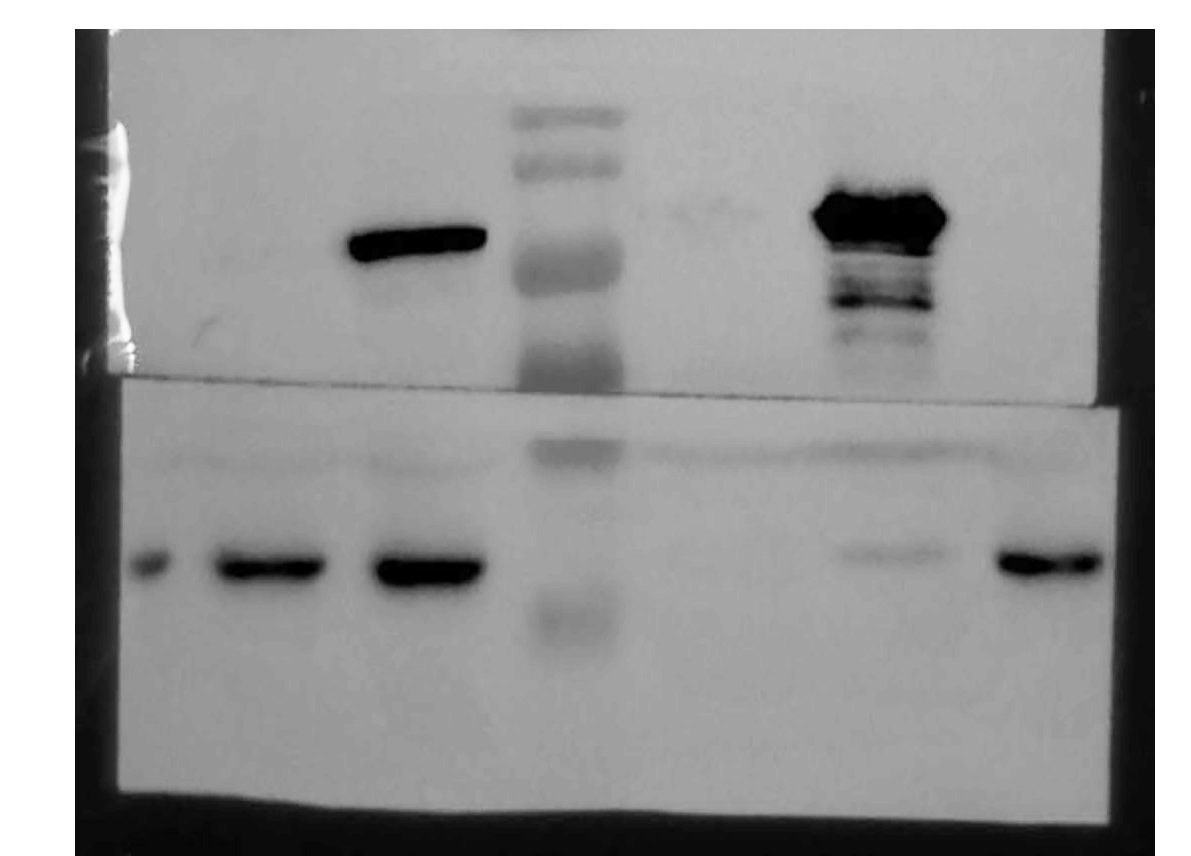

Supplement: Figure 6—source data 2. [file elife-100497-fig6-data2.zip › Figure 6-source data 2/Figure 6B-source data 2/Input BCAS2.tif]

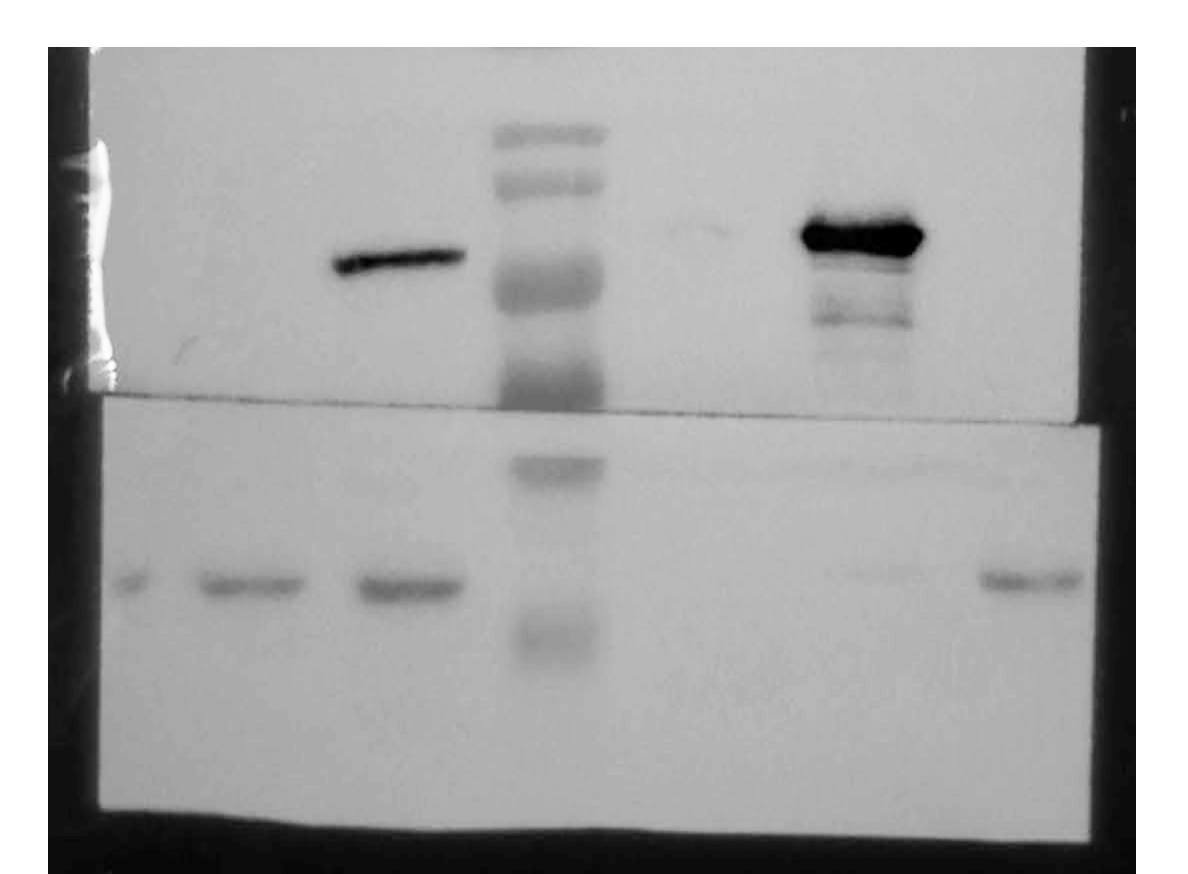

Supplement: Figure 6—source data 2. [file elife-100497-fig6-data2.zip › Figure 6-source data 2/Figure 6B-source data 2/IP input Bcatenin.tif]

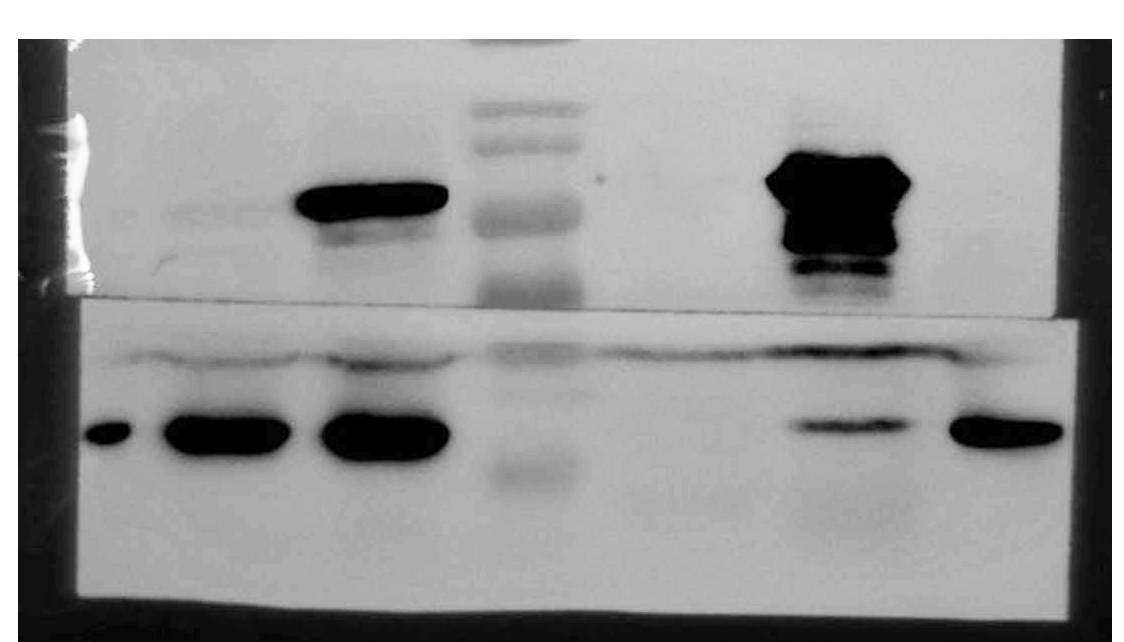

Supplement: Figure 6—source data 2. [file elife-100497-fig6-data2.zip › Figure 6-source data 2/Figure 6B-source data 2/IP半5.tif]

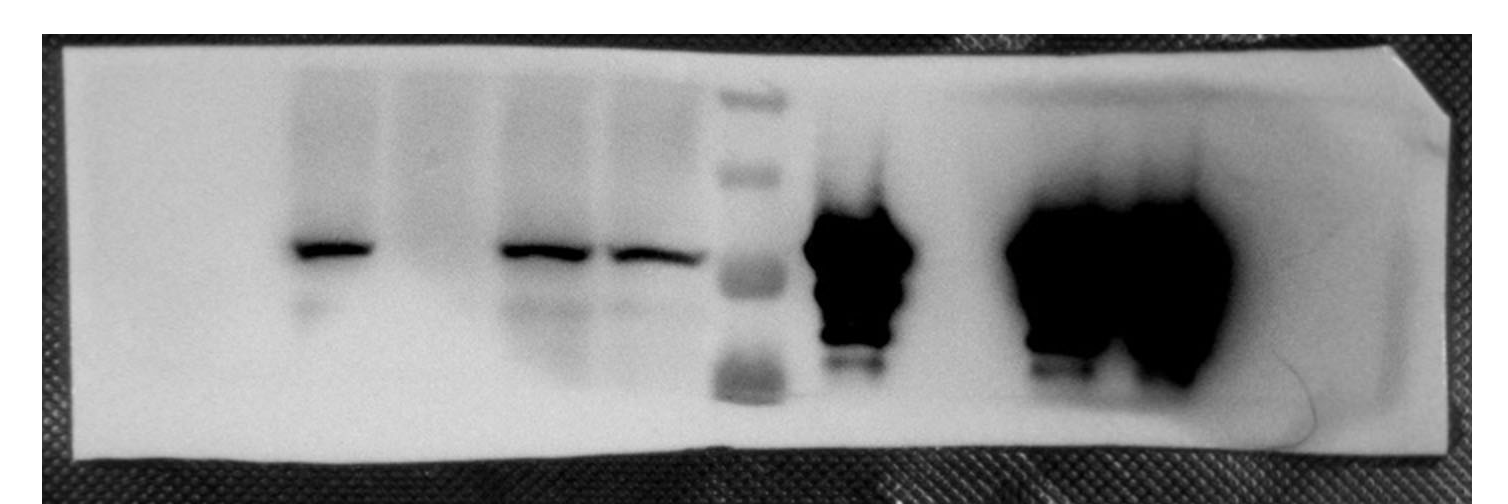

Supplement: Figure 6—source data 2. [file elife-100497-fig6-data2.zip › Figure 6-source data 2/Figure 6C-source data 2/ip wnt bcat 2.tif]

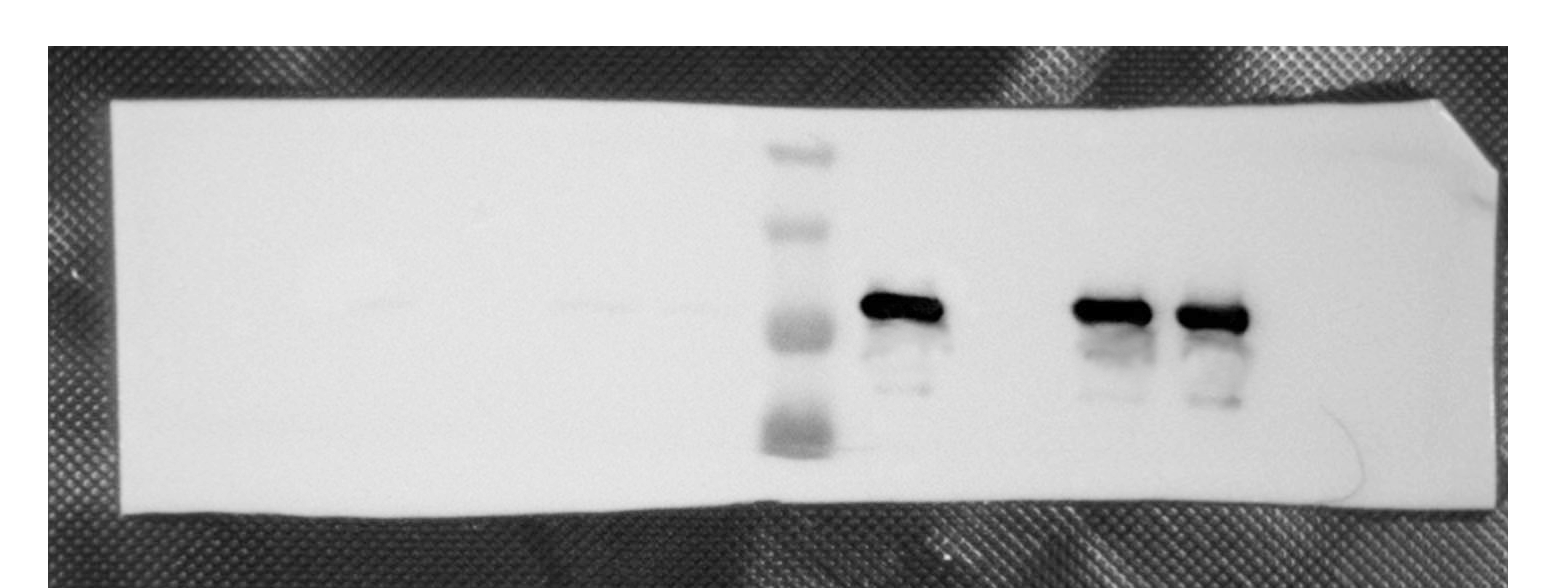

Supplement: Figure 6—source data 2. [file elife-100497-fig6-data2.zip › Figure 6-source data 2/Figure 6C-source data 2/ip wnt bcat.tif]

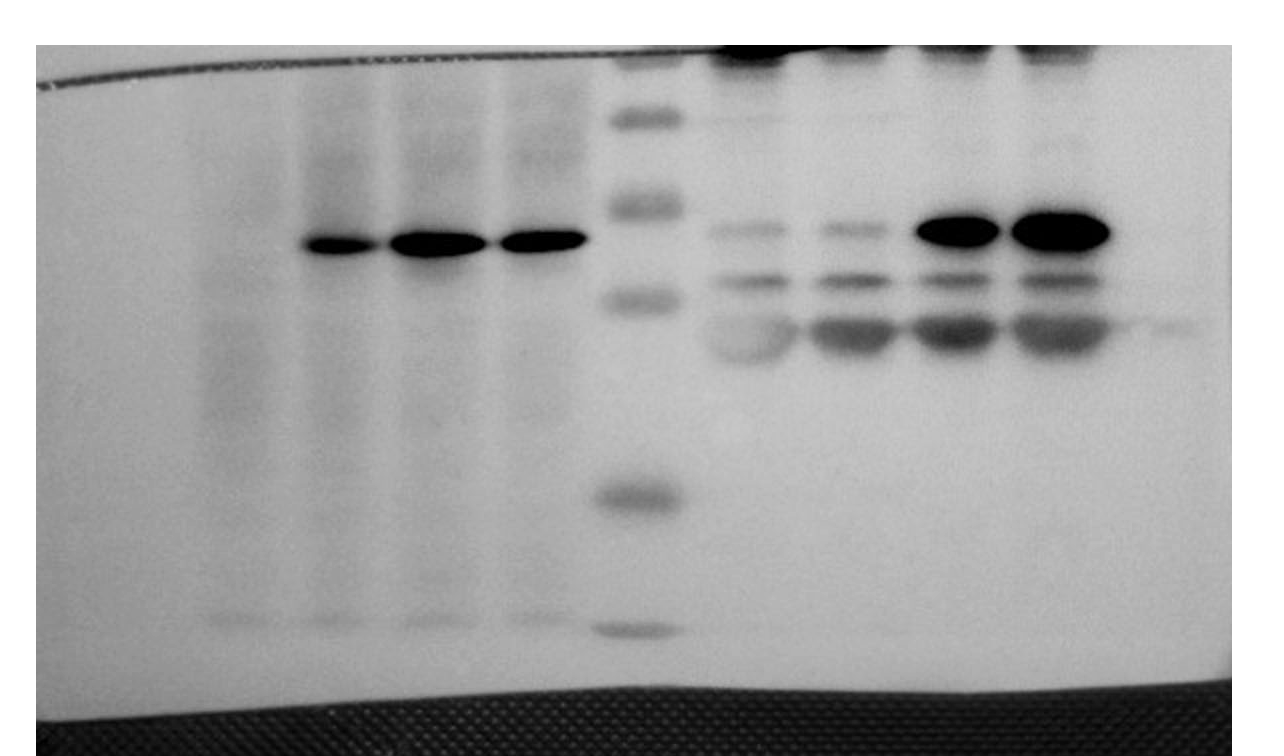

Supplement: Figure 6—source data 2. [file elife-100497-fig6-data2.zip › Figure 6-source data 2/Figure 6C-source data 2/ip wnt3 input.tif]

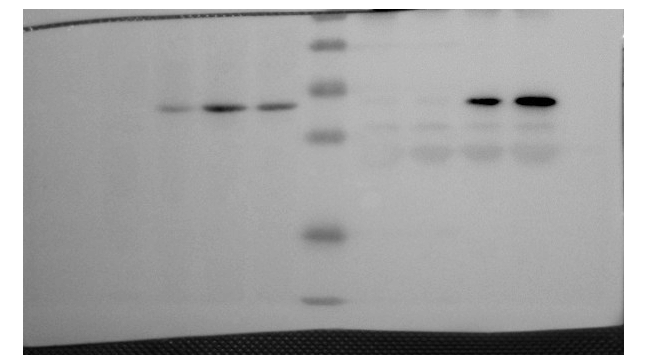

Supplement: Figure 6—source data 2. [file elife-100497-fig6-data2.zip › Figure 6-source data 2/Figure 6C-source data 2/ip wnt3.tif]

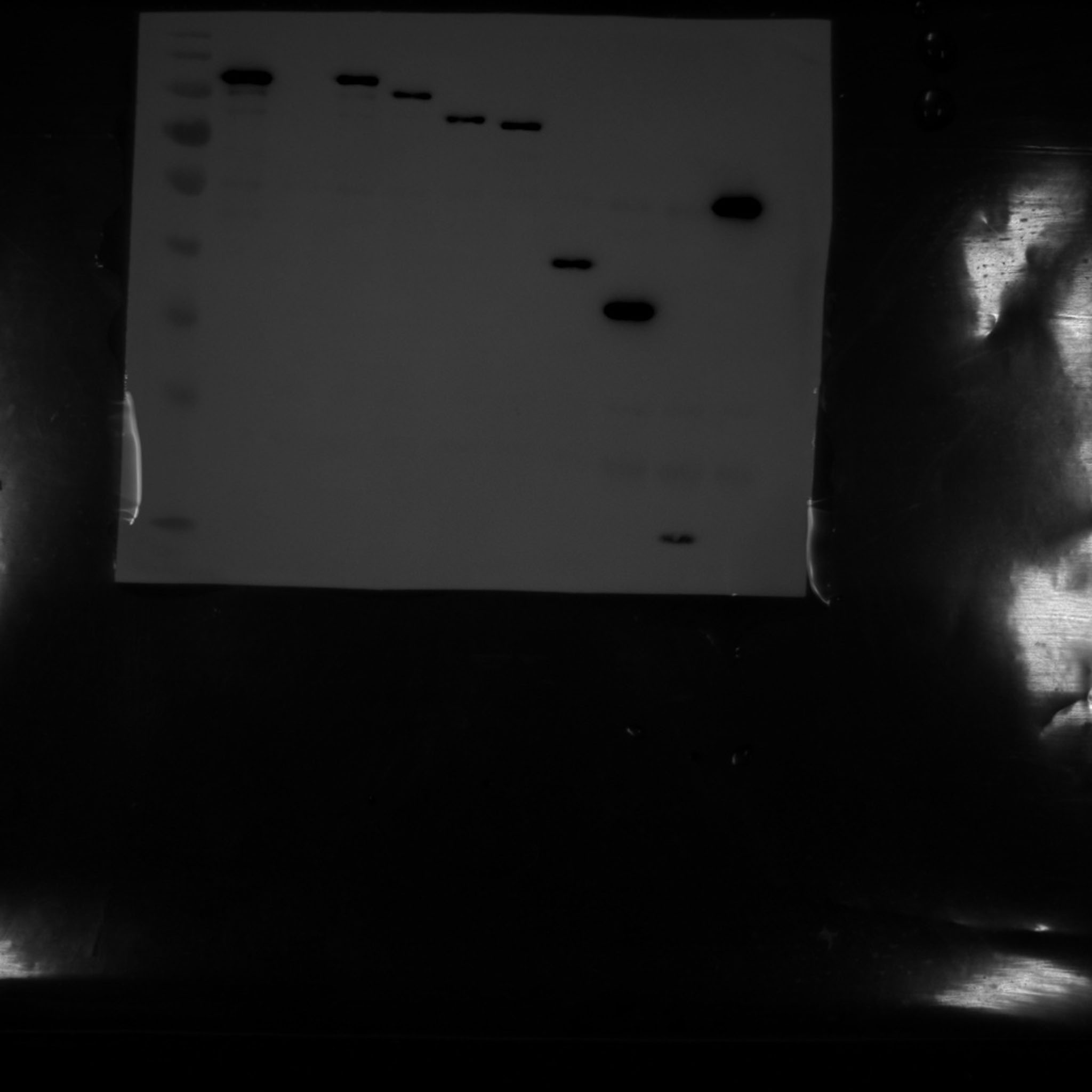

Supplement: Figure 6—source data 2. [file elife-100497-fig6-data2.zip › Figure 6-source data 2/Figure 6G-source data 2/BCAT DELEPTION 3.jpg]

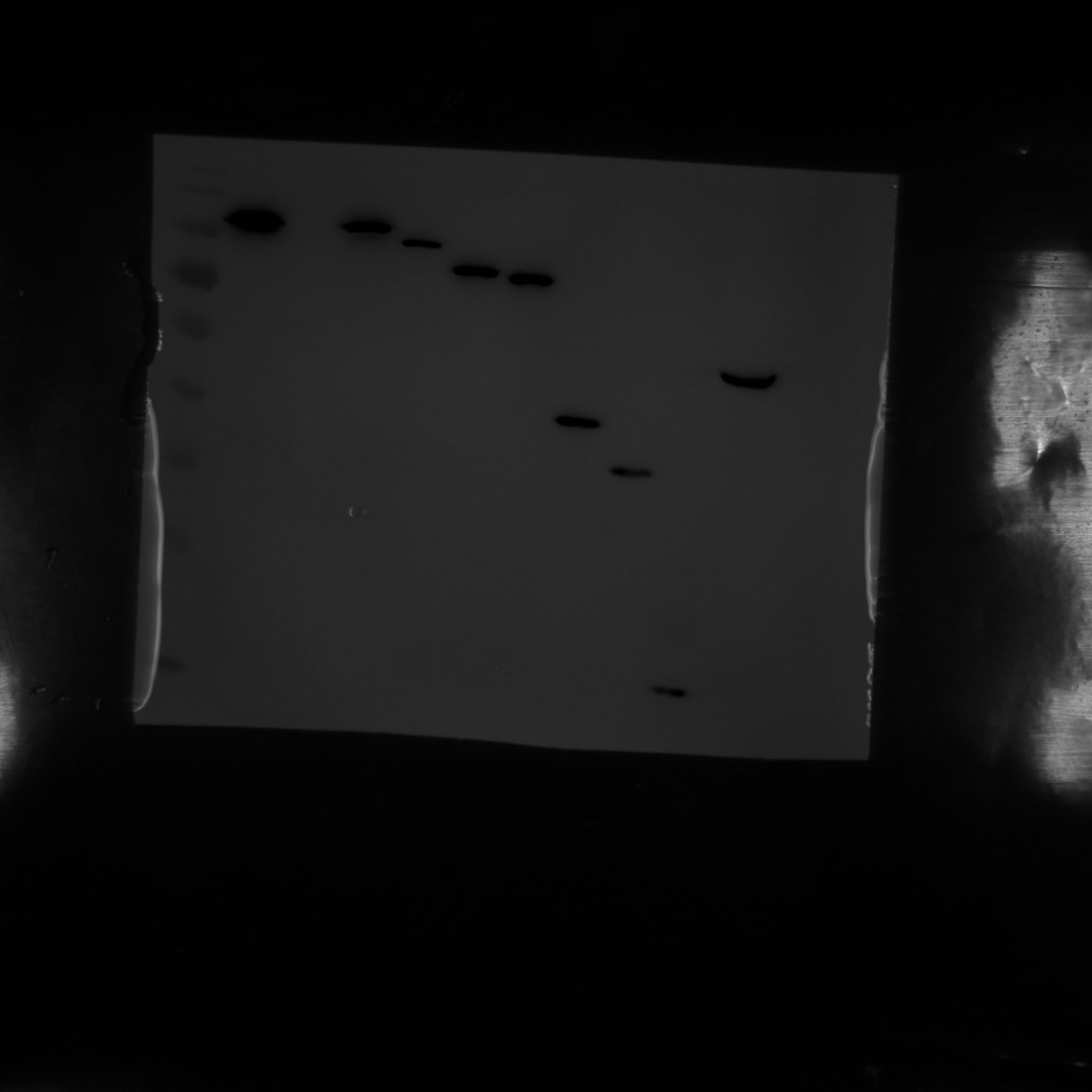

Supplement: Figure 6—source data 2. [file elife-100497-fig6-data2.zip › Figure 6-source data 2/Figure 6G-source data 2/BCAT DELEPTION.jpg]

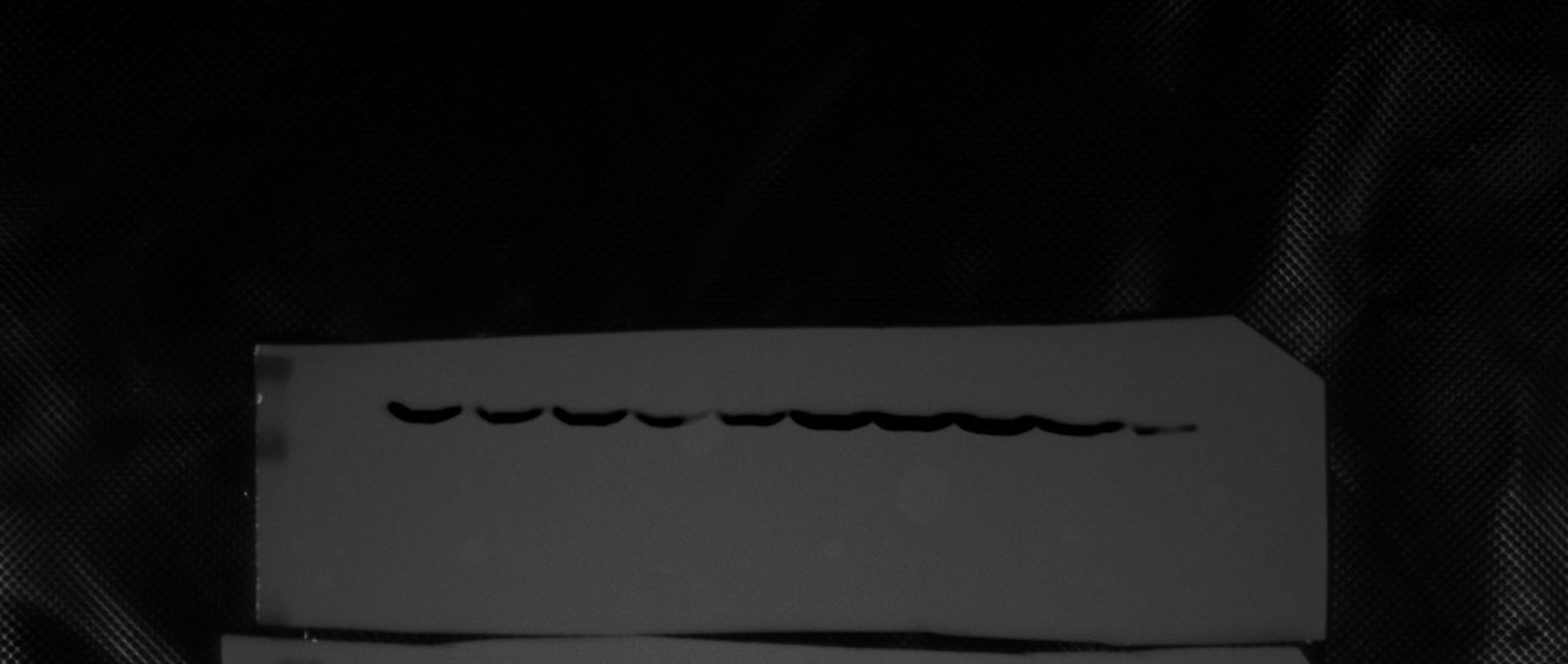

Supplement: Figure 6—source data 2. [file elife-100497-fig6-data2.zip › Figure 6-source data 2/Figure 6G-source data 2/INPUT 1.jpg]

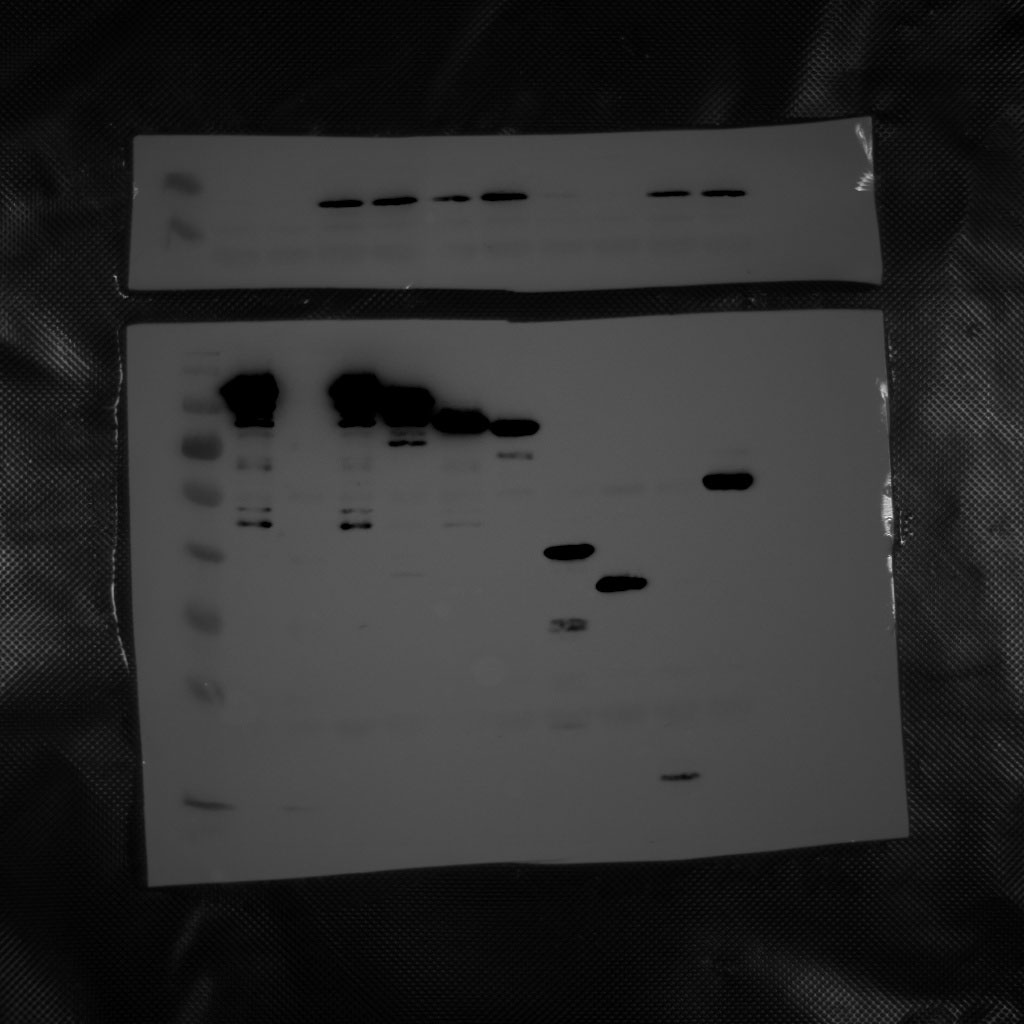

Supplement: Figure 6—source data 2. [file elife-100497-fig6-data2.zip › Figure 6-source data 2/Figure 6G-source data 2/ip bcas2.jpg]

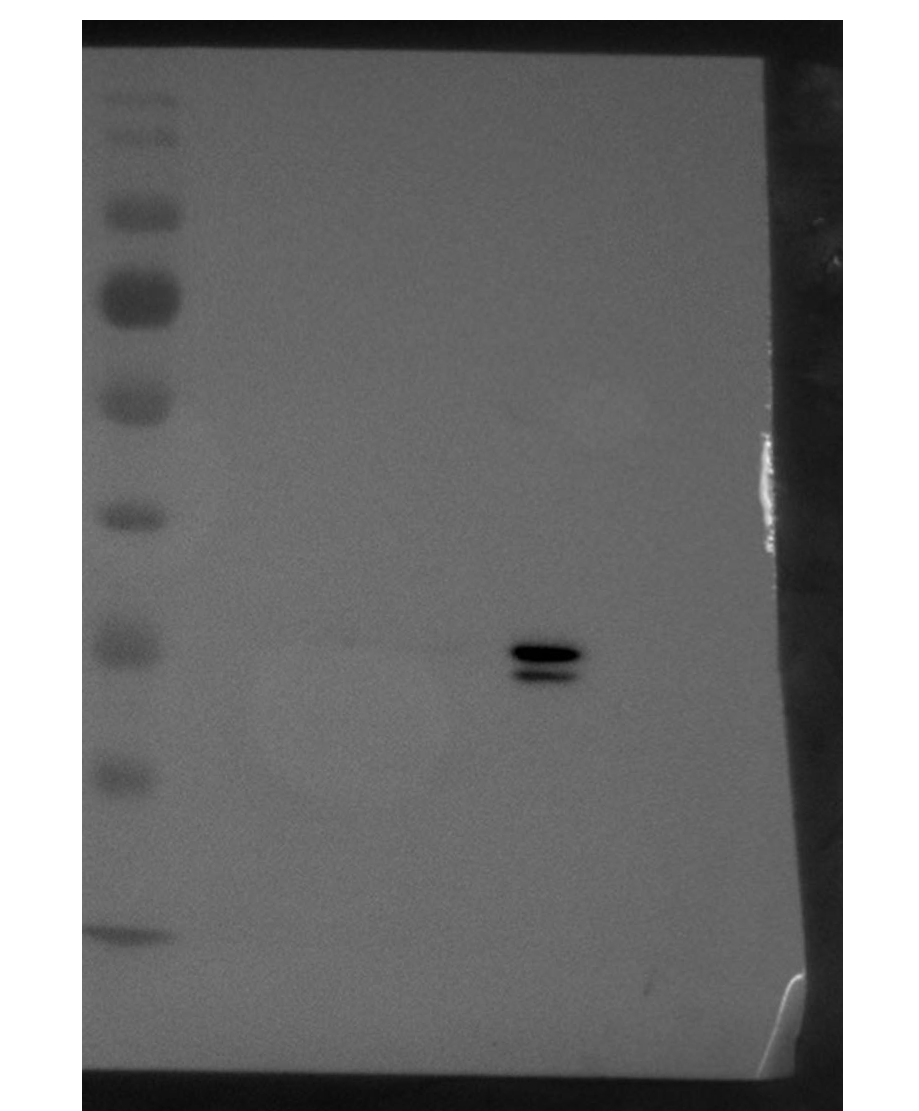

Supplement: Figure 6—source data 2. [file elife-100497-fig6-data2.zip › Figure 6-source data 2/Figure 6H-source data 2/his-BCAS2.tif]

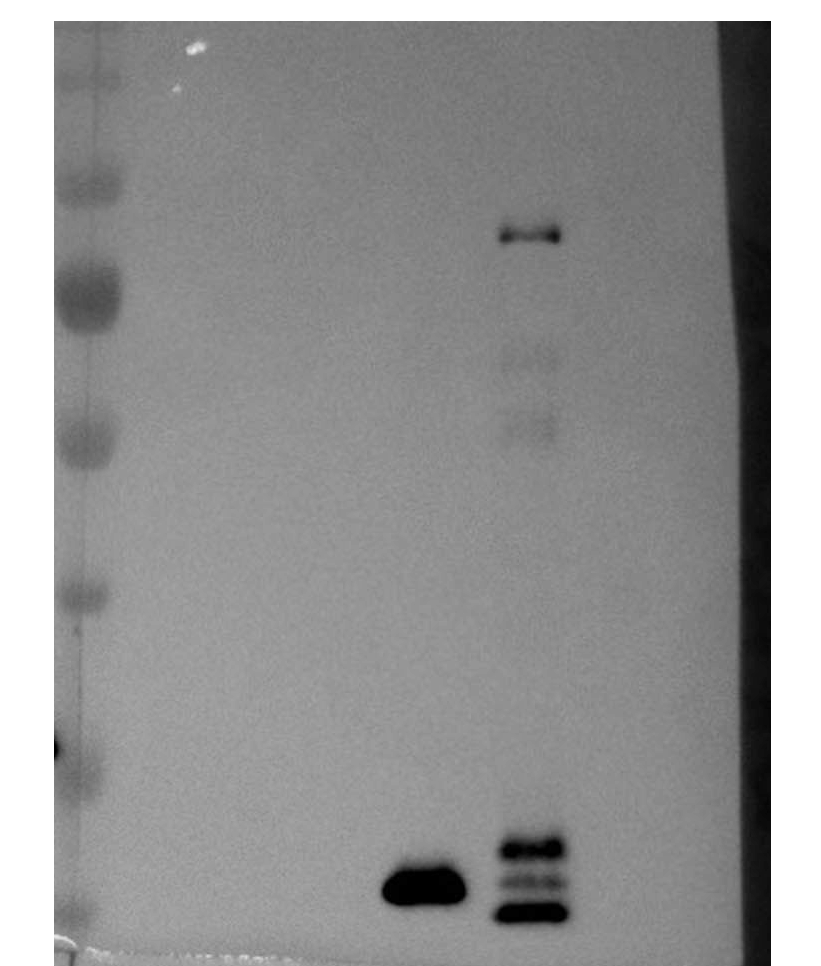

Supplement: Figure 6—source data 2. [file elife-100497-fig6-data2.zip › Figure 6-source data 2/Figure 6H-source data 2/input pull down GST.tif]

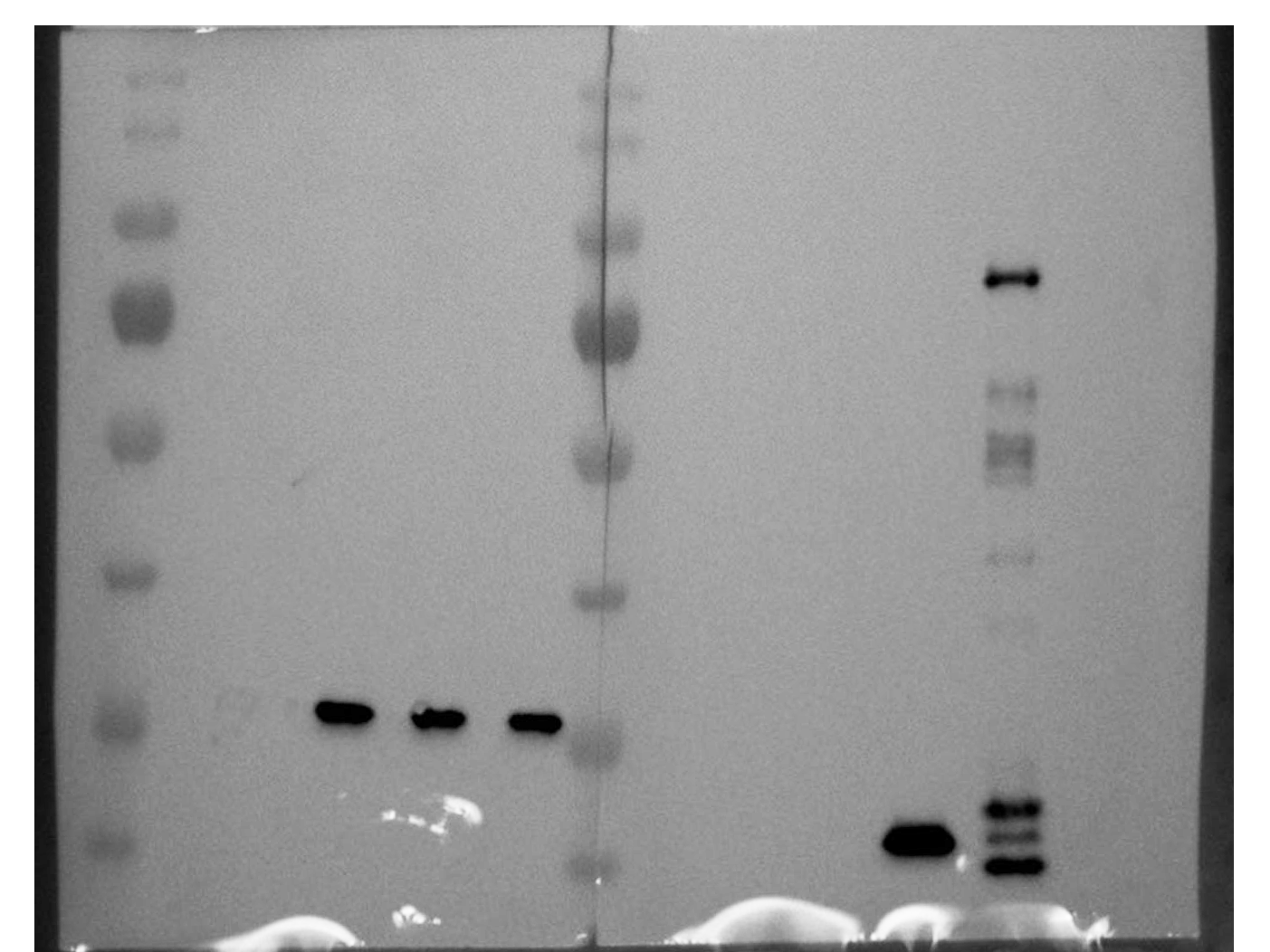

Supplement: Figure 6—source data 2. [file elife-100497-fig6-data2.zip › Figure 6-source data 2/Figure 6H-source data 2/input pull down GST2.tif]

Figure 6-figure supplement 1

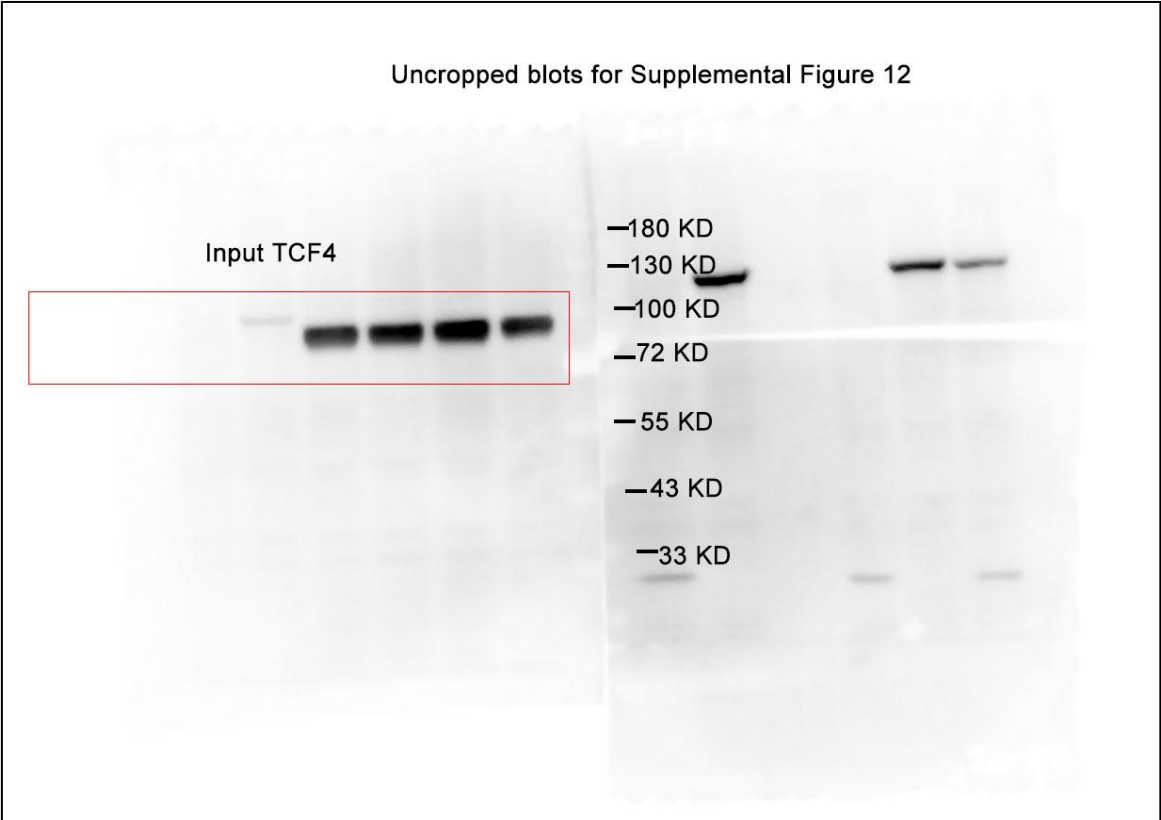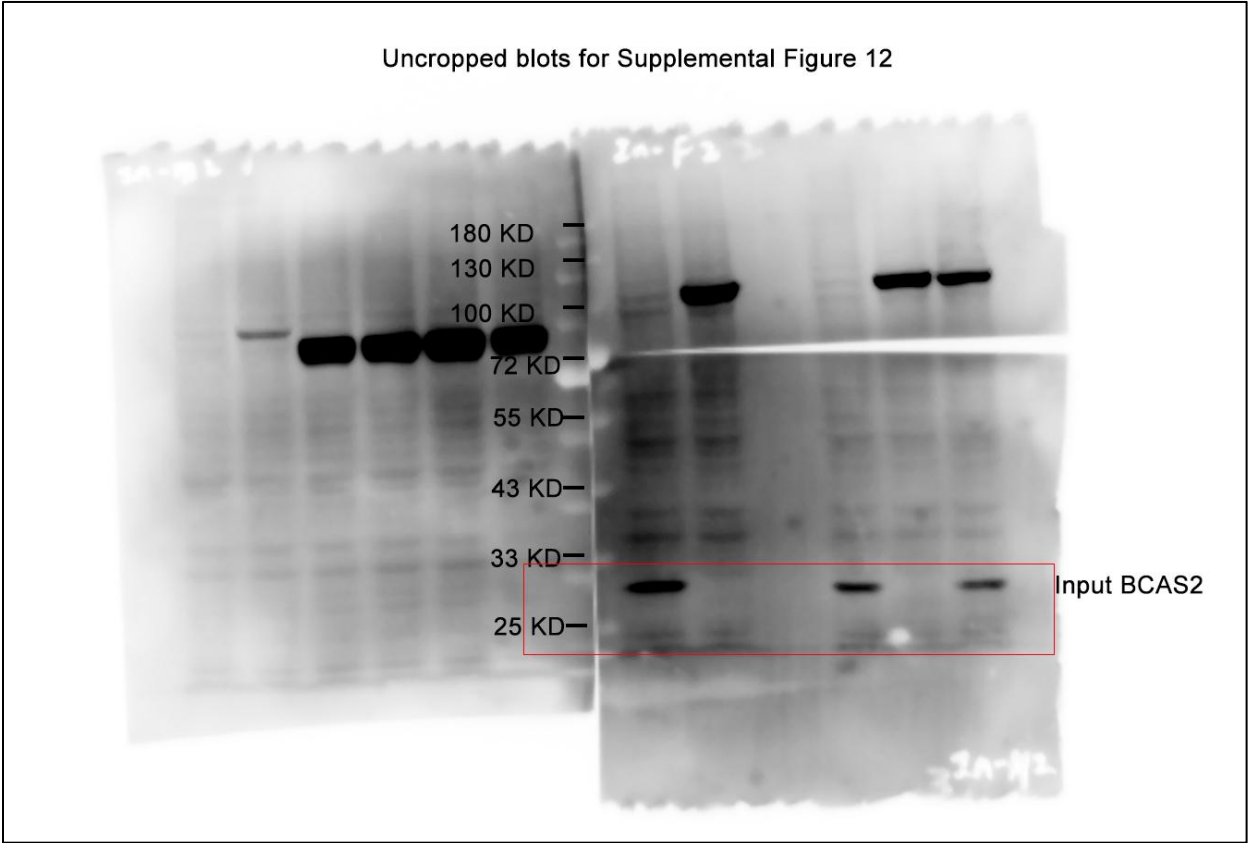

Figure 6-figure supplement 1

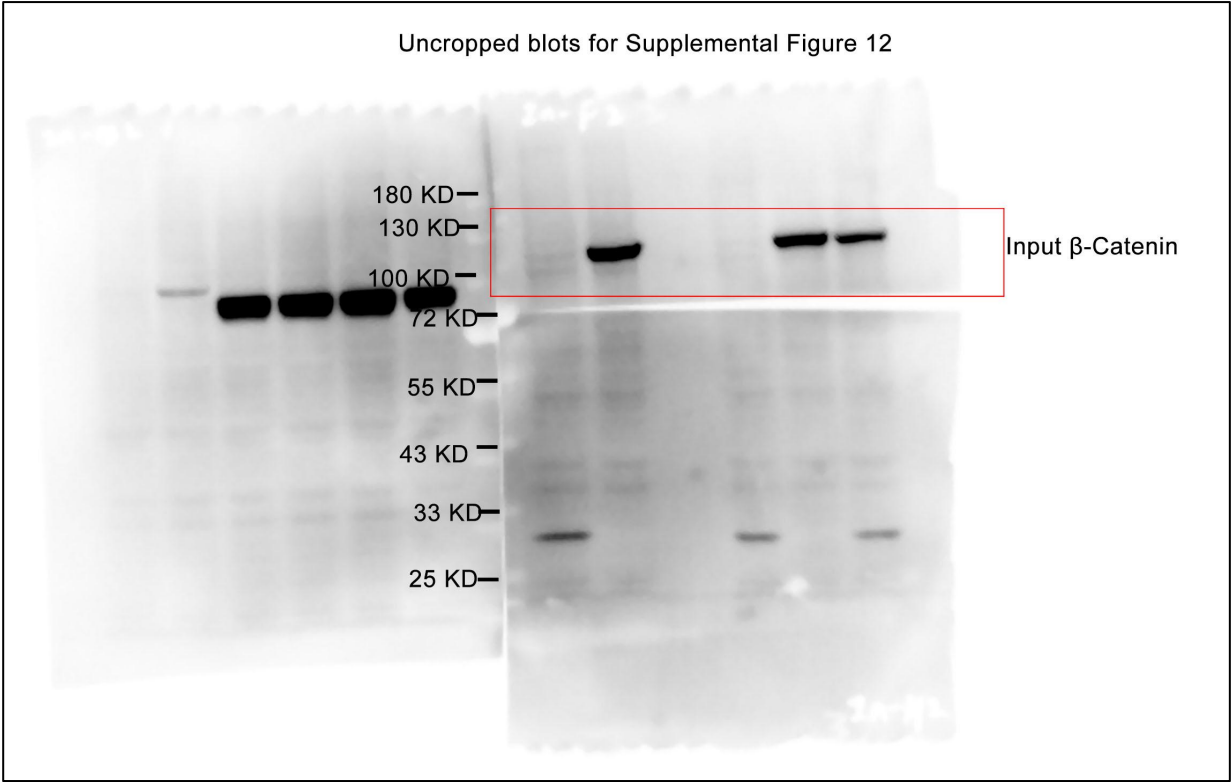

Figure 6-figure supplement 1

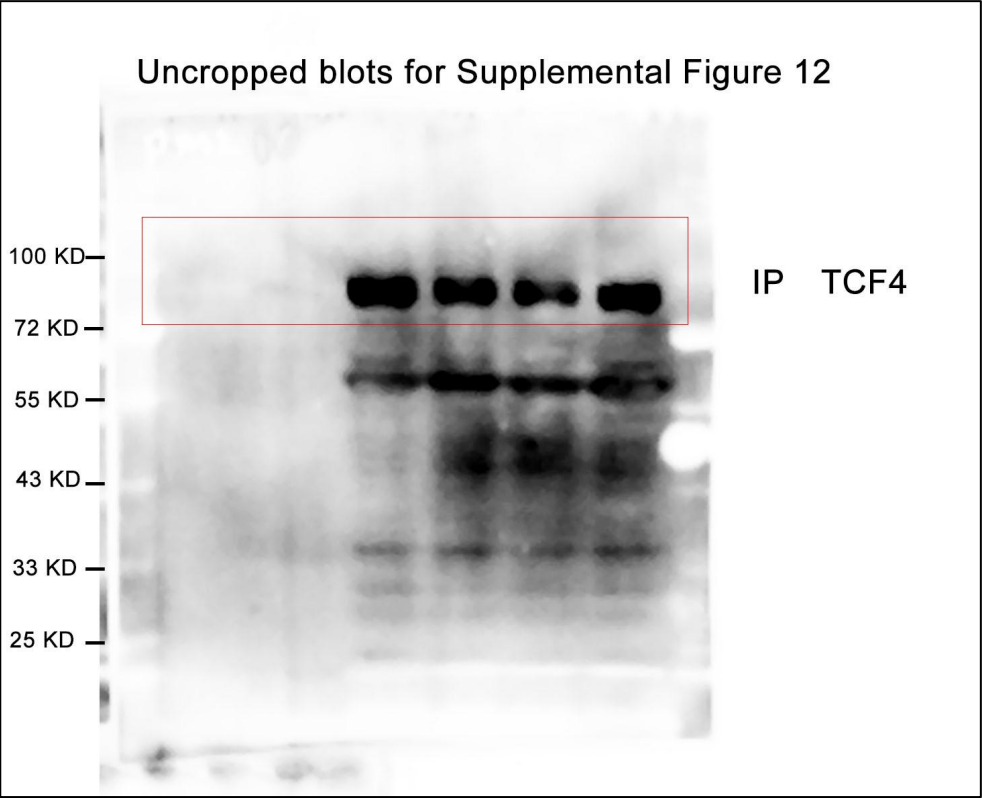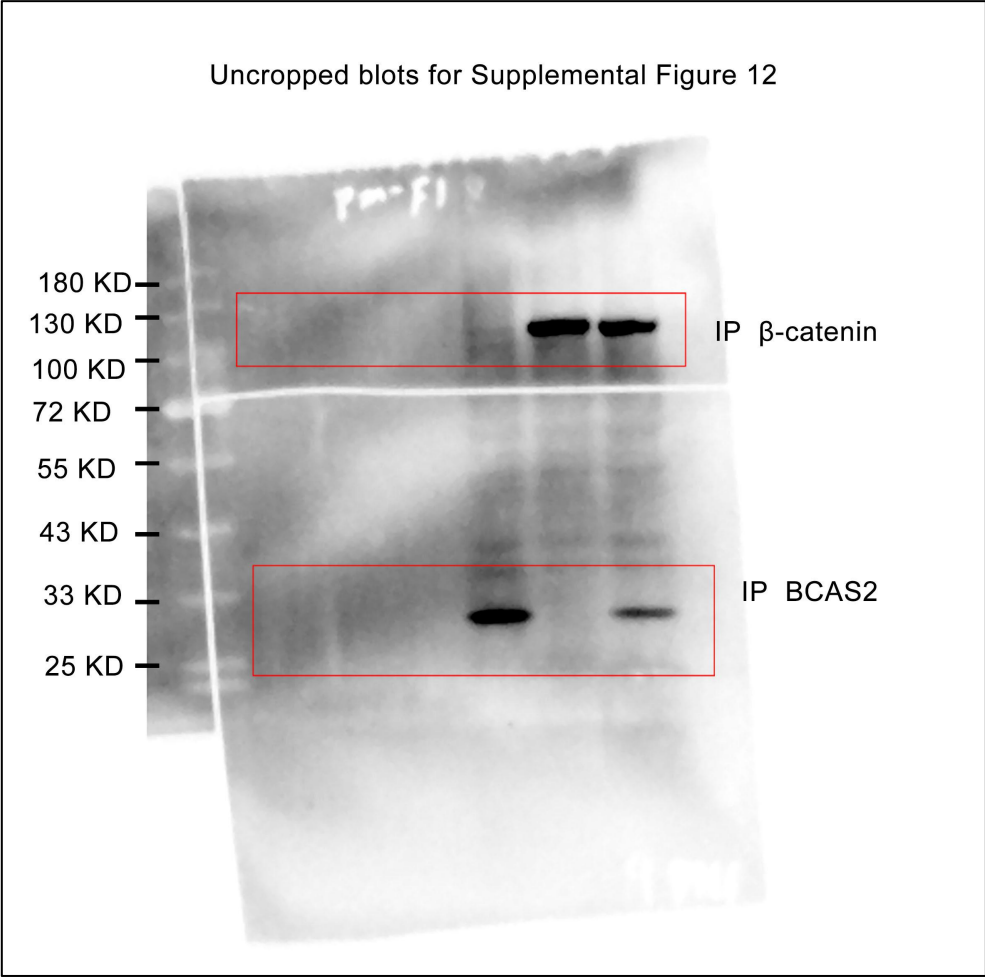

Supplement: Figure 6—figure supplement 1—source data 1. [file elife-100497-fig6-figsupp1-data1.zip › Figure 6-figure supplement 1-source data 1.pdf]

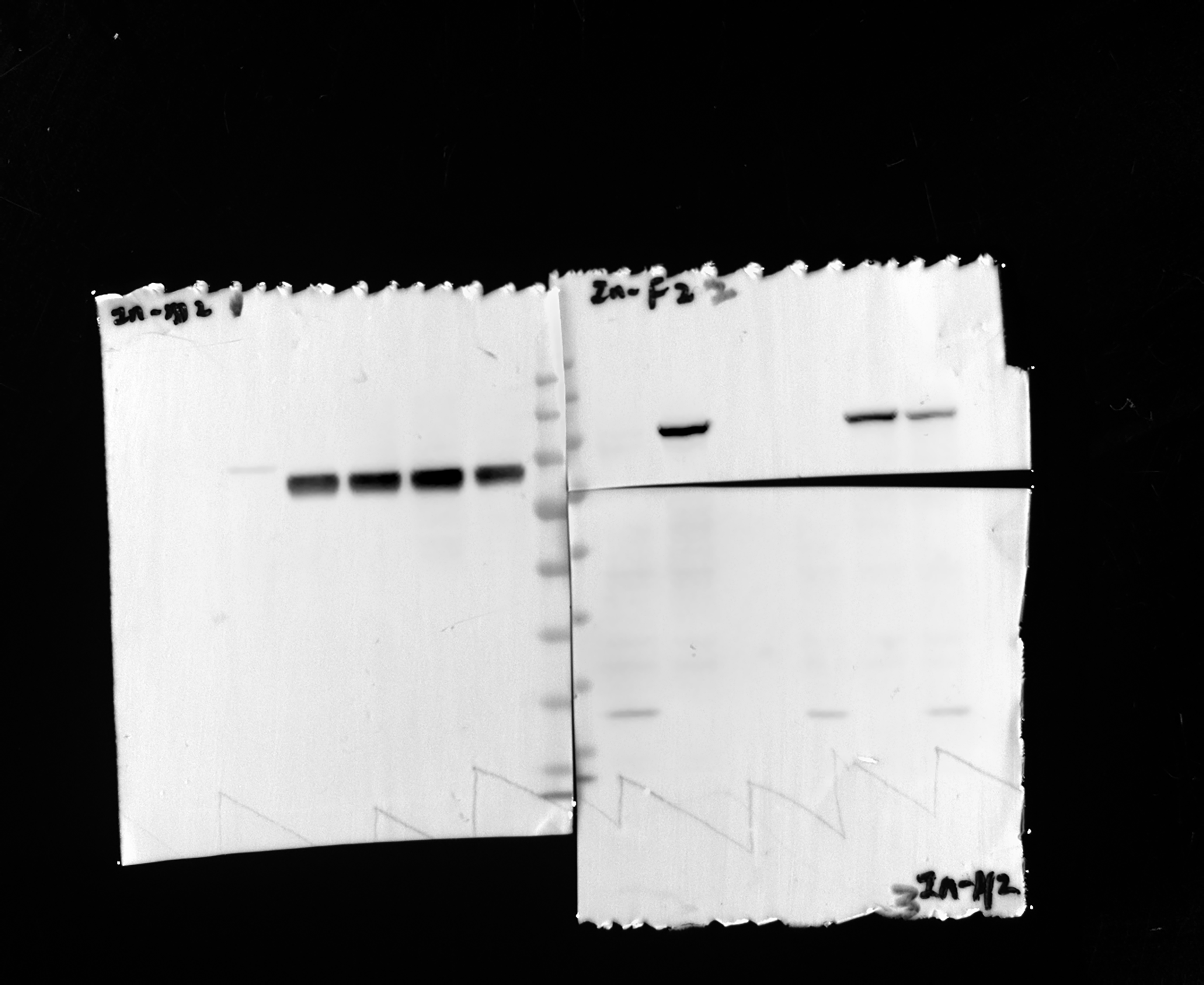

Supplement: Figure 6—figure supplement 1—source data 2. [file elife-100497-fig6-figsupp1-data2.zip › Figure 6-figure supplement 1-source data 2/input/007[GA]-20250320-150126-luminescence-overlay.tif]

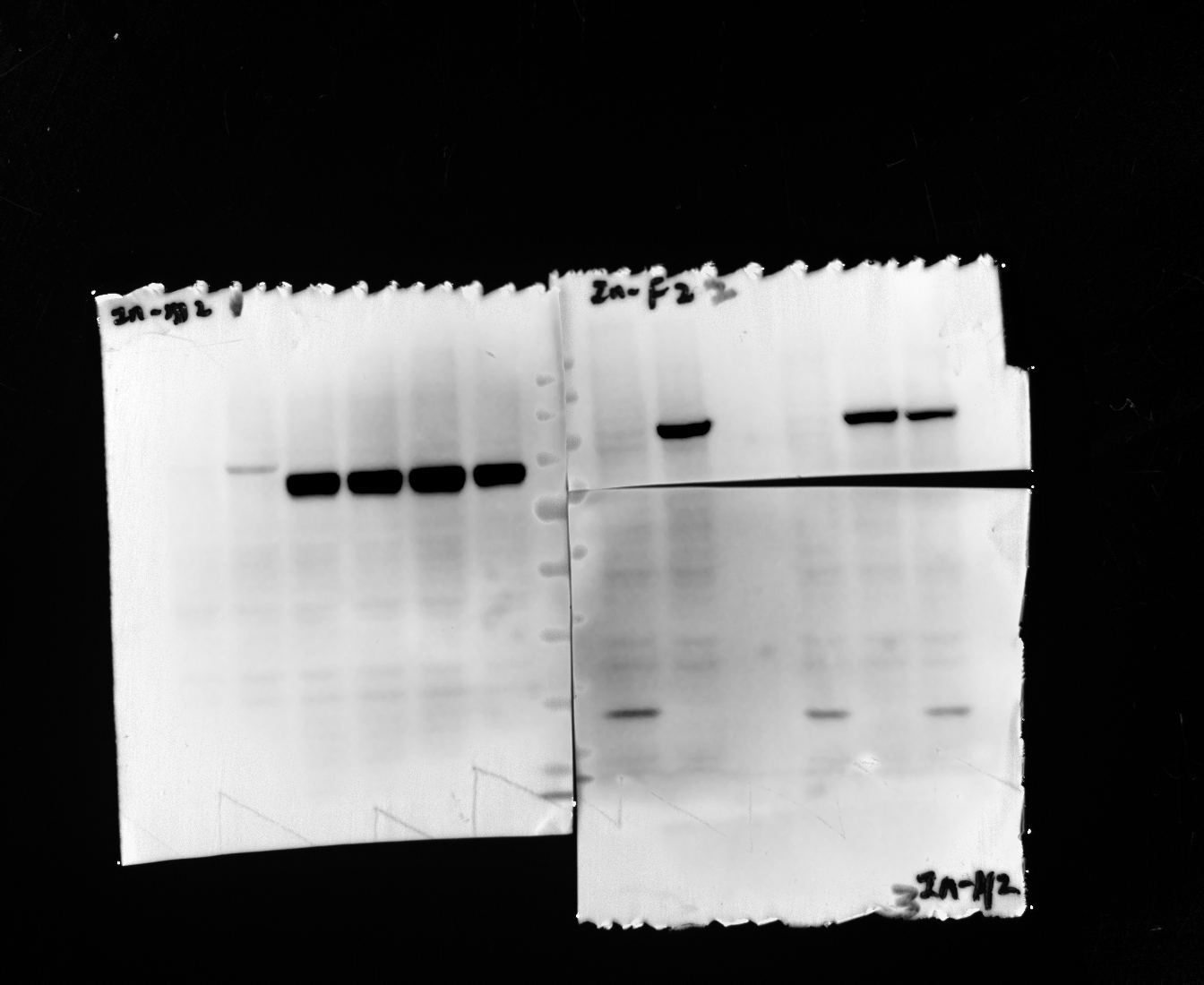

Supplement: Figure 6—figure supplement 1—source data 2. [file elife-100497-fig6-figsupp1-data2.zip › Figure 6-figure supplement 1-source data 2/input/008[GA]-20250320-150127-luminescence-overlay.tif]

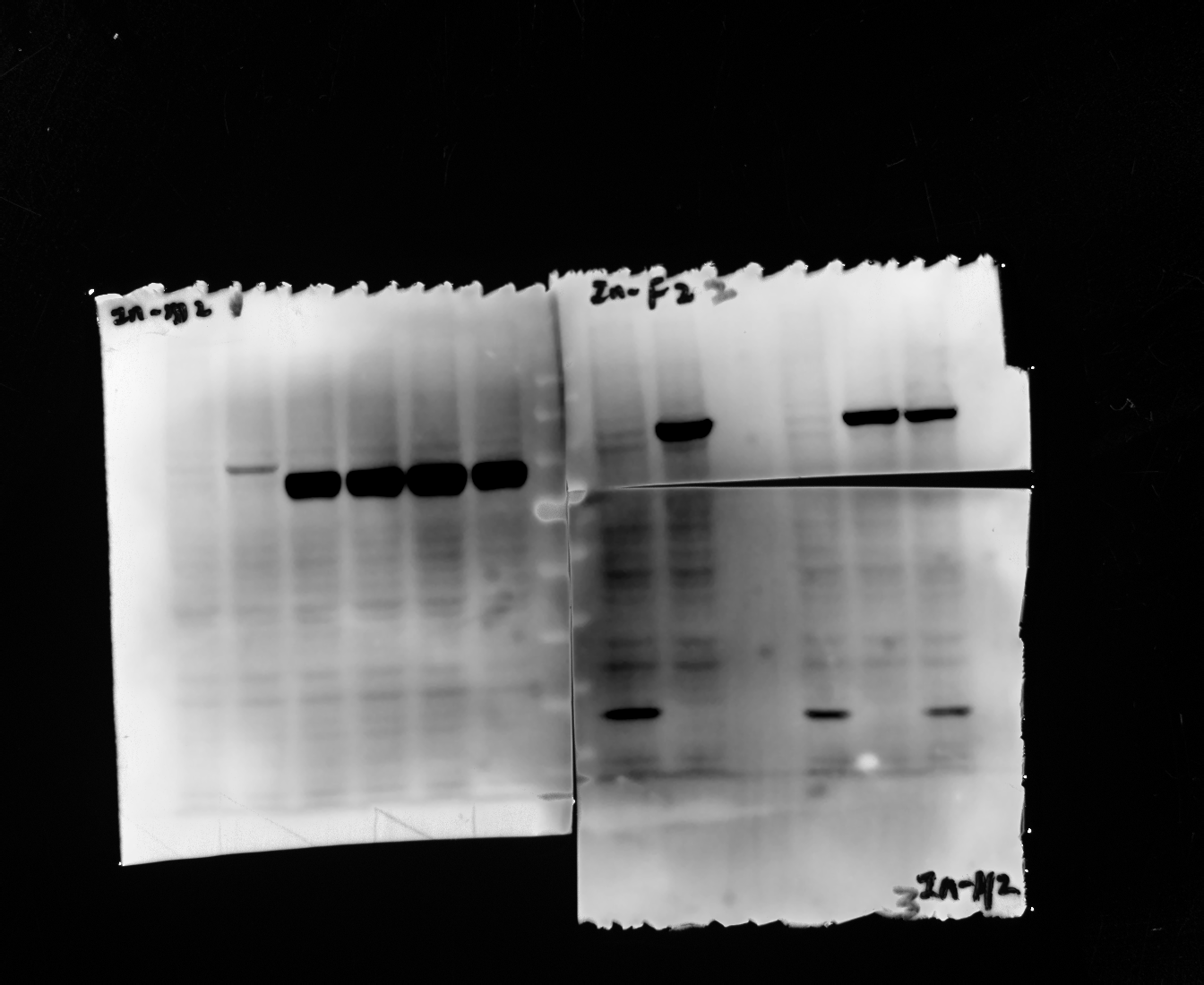

Supplement: Figure 6—figure supplement 1—source data 2. [file elife-100497-fig6-figsupp1-data2.zip › Figure 6-figure supplement 1-source data 2/input/010[GA]-20250320-150127-luminescence-overlay.tif]

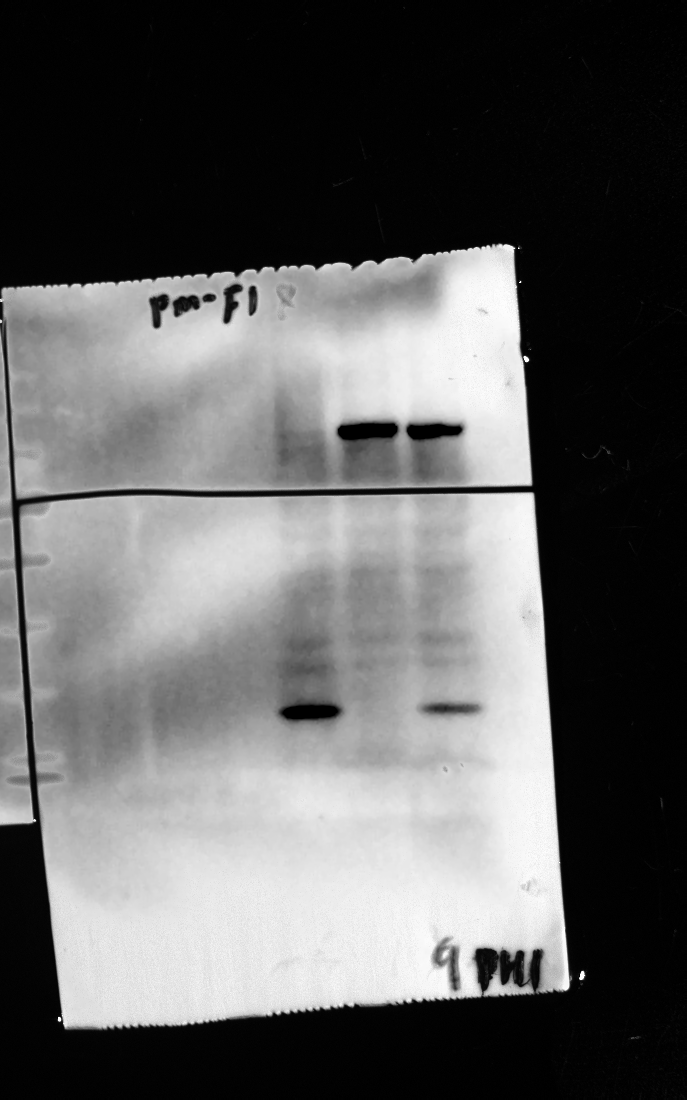

Supplement: Figure 6—figure supplement 1—source data 2. [file elife-100497-fig6-figsupp1-data2.zip › Figure 6-figure supplement 1-source data 2/IP/003[GA]-20250320-150602-luminescence-overlay.tif]

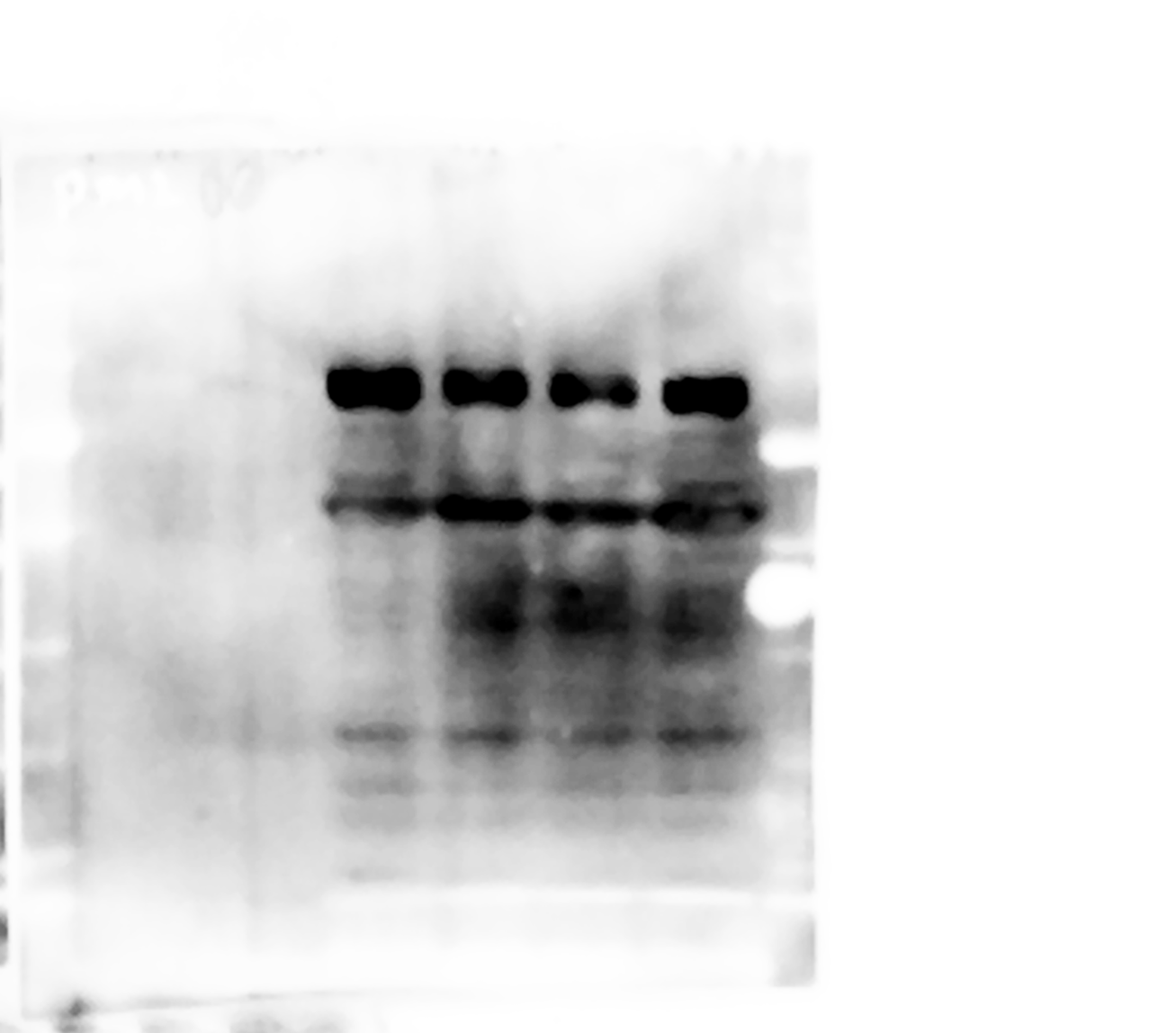

Supplement: Figure 6—figure supplement 1—source data 2. [file elife-100497-fig6-figsupp1-data2.zip › Figure 6-figure supplement 1-source data 2/IP/003[si Selp IP ubiquitin]-20250322-103000-luminescence.tif]

Figure 7B

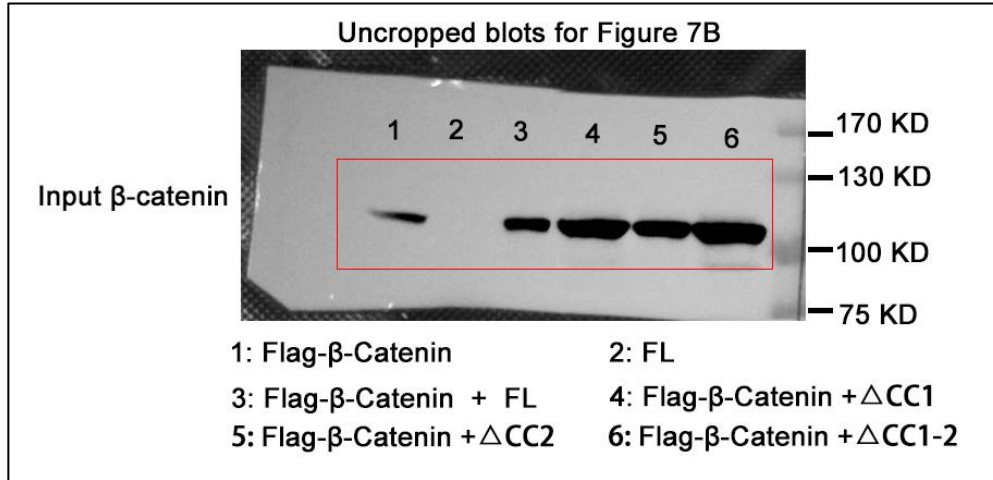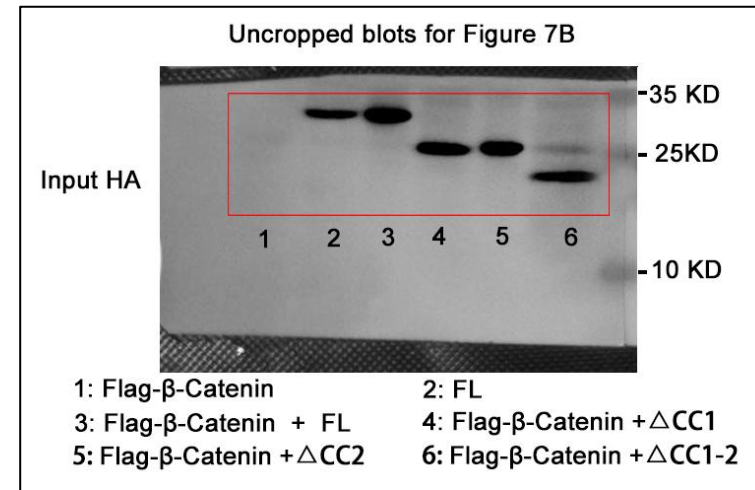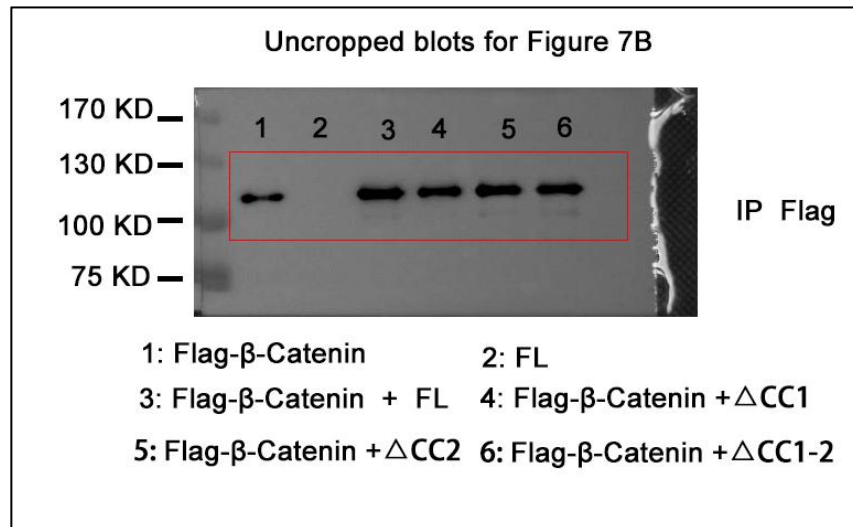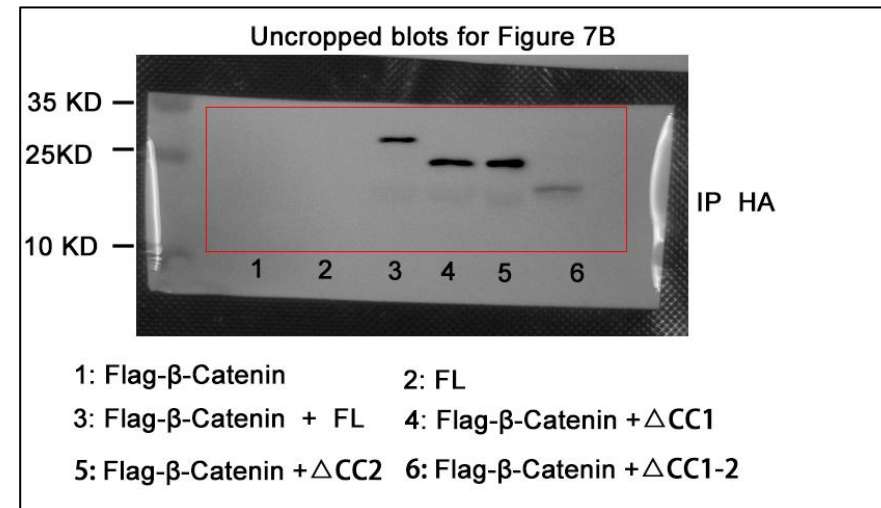

Figure 7C

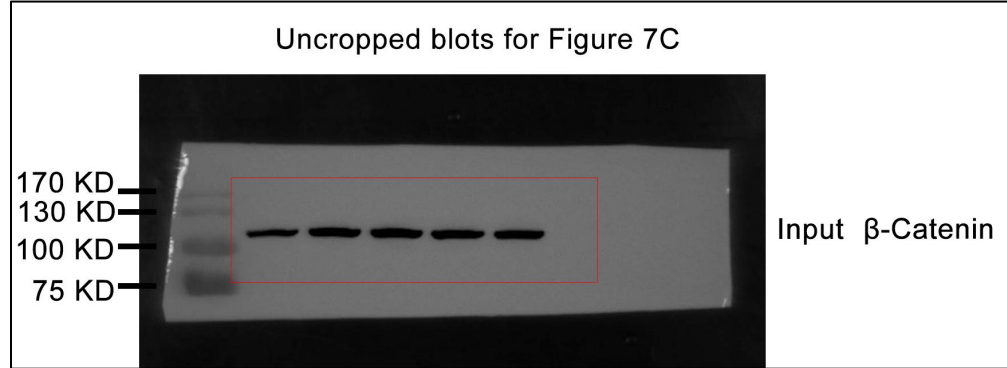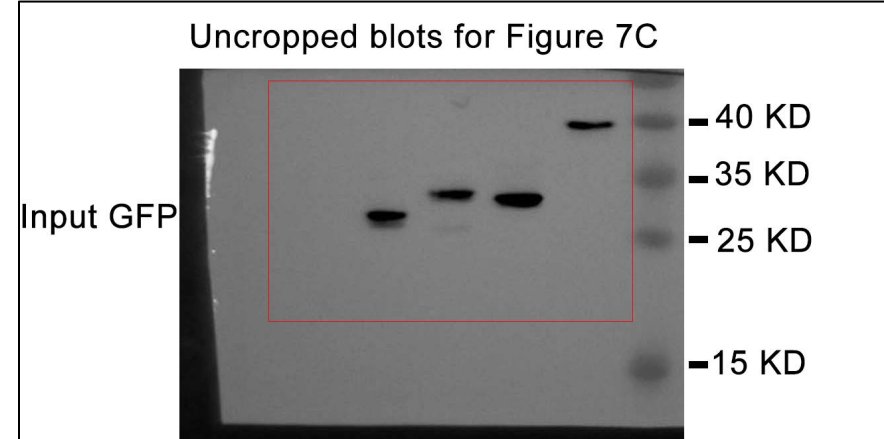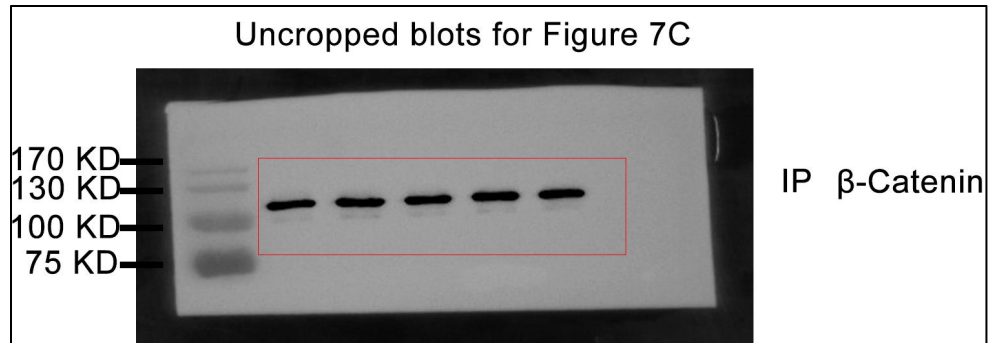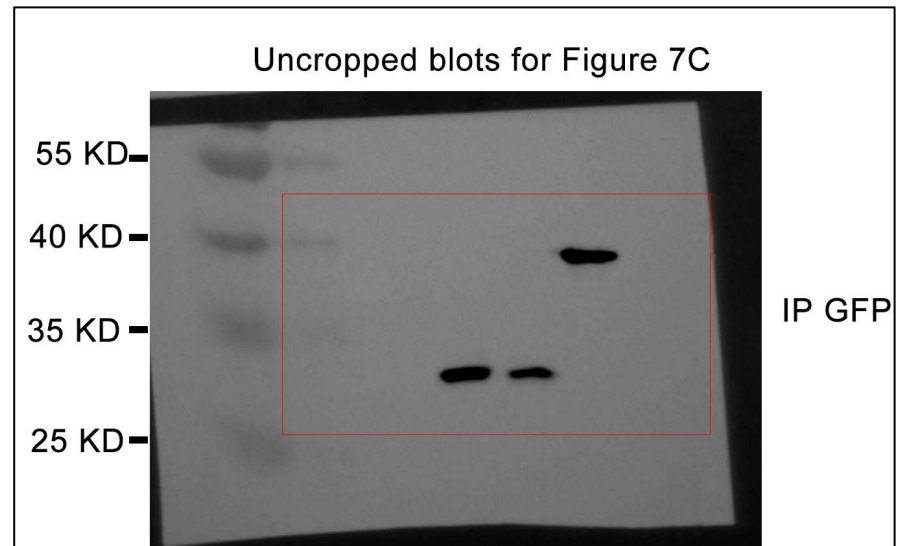

Supplement: Figure 7—source data 1. [file elife-100497-fig7-data1.zip › Figure 7-source data 1.pdf]

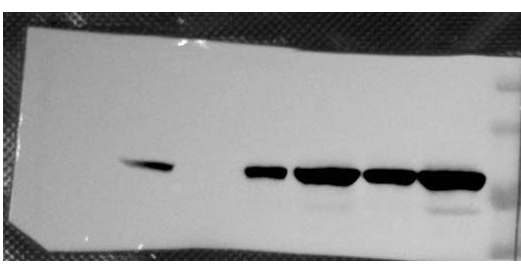

Supplement: Figure 7—source data 2. [file elife-100497-fig7-data2.zip › Figure 7-source data 2/Figure 7B-source data 2/INPUT flag b-catenin.tif]

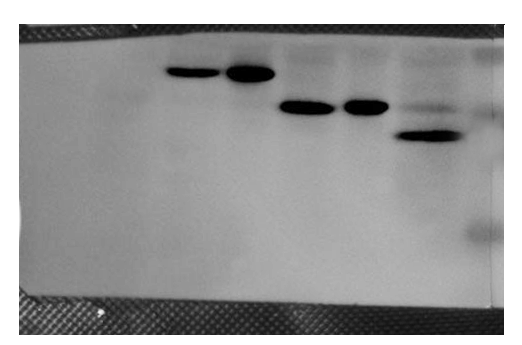

Supplement: Figure 7—source data 2. [file elife-100497-fig7-data2.zip › Figure 7-source data 2/Figure 7B-source data 2/INPUT.tif]

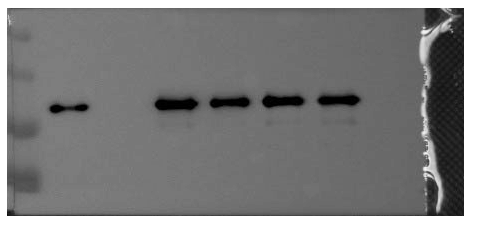

Supplement: Figure 7—source data 2. [file elife-100497-fig7-data2.zip › Figure 7-source data 2/Figure 7B-source data 2/IP flag.tif]

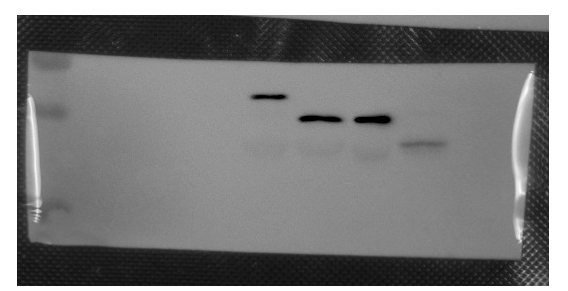

Supplement: Figure 7—source data 2. [file elife-100497-fig7-data2.zip › Figure 7-source data 2/Figure 7B-source data 2/IP HA.tif]

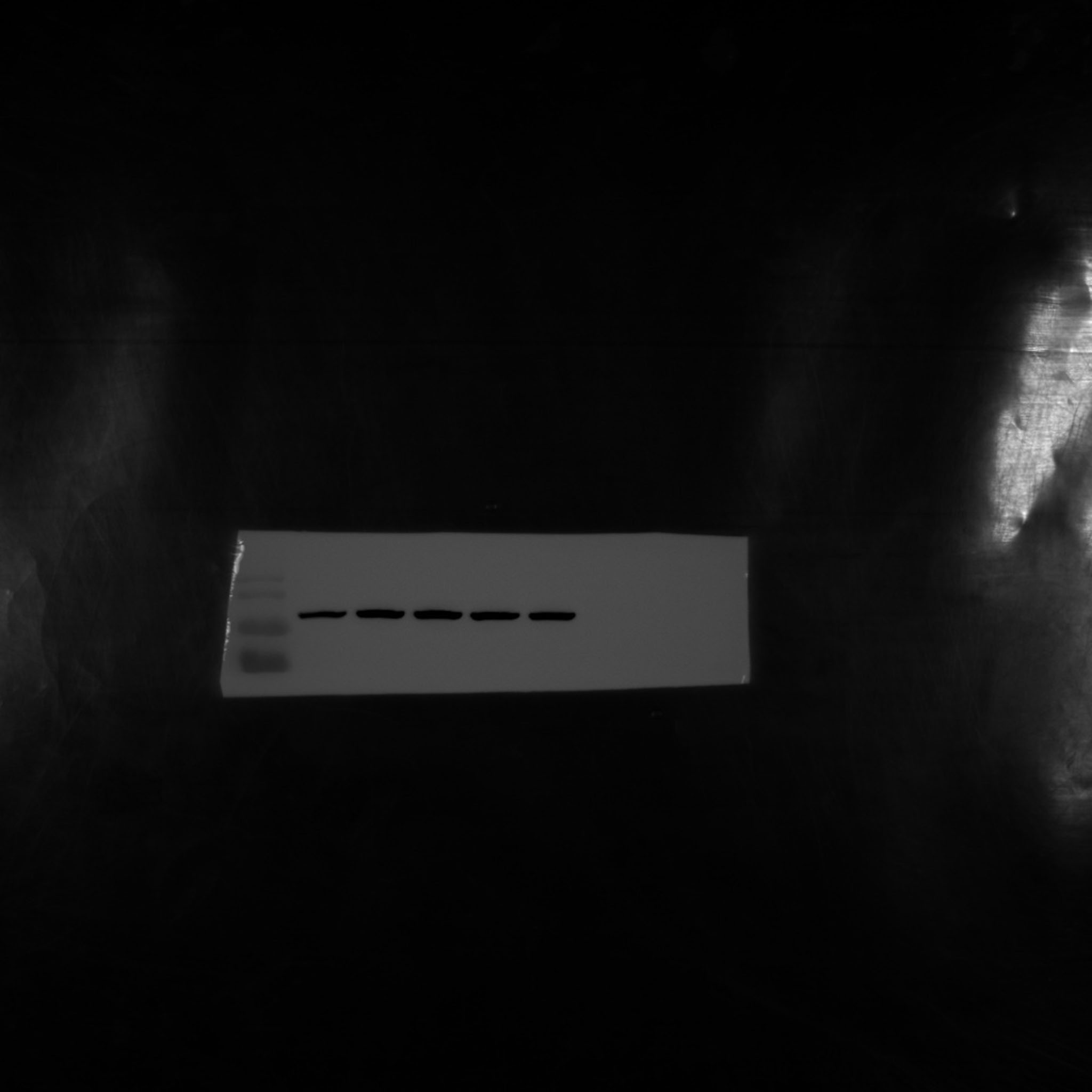

Supplement: Figure 7—source data 2. [file elife-100497-fig7-data2.zip › Figure 7-source data 2/Figure 7C-source data 2/Input Flag B-catenin.jpg]

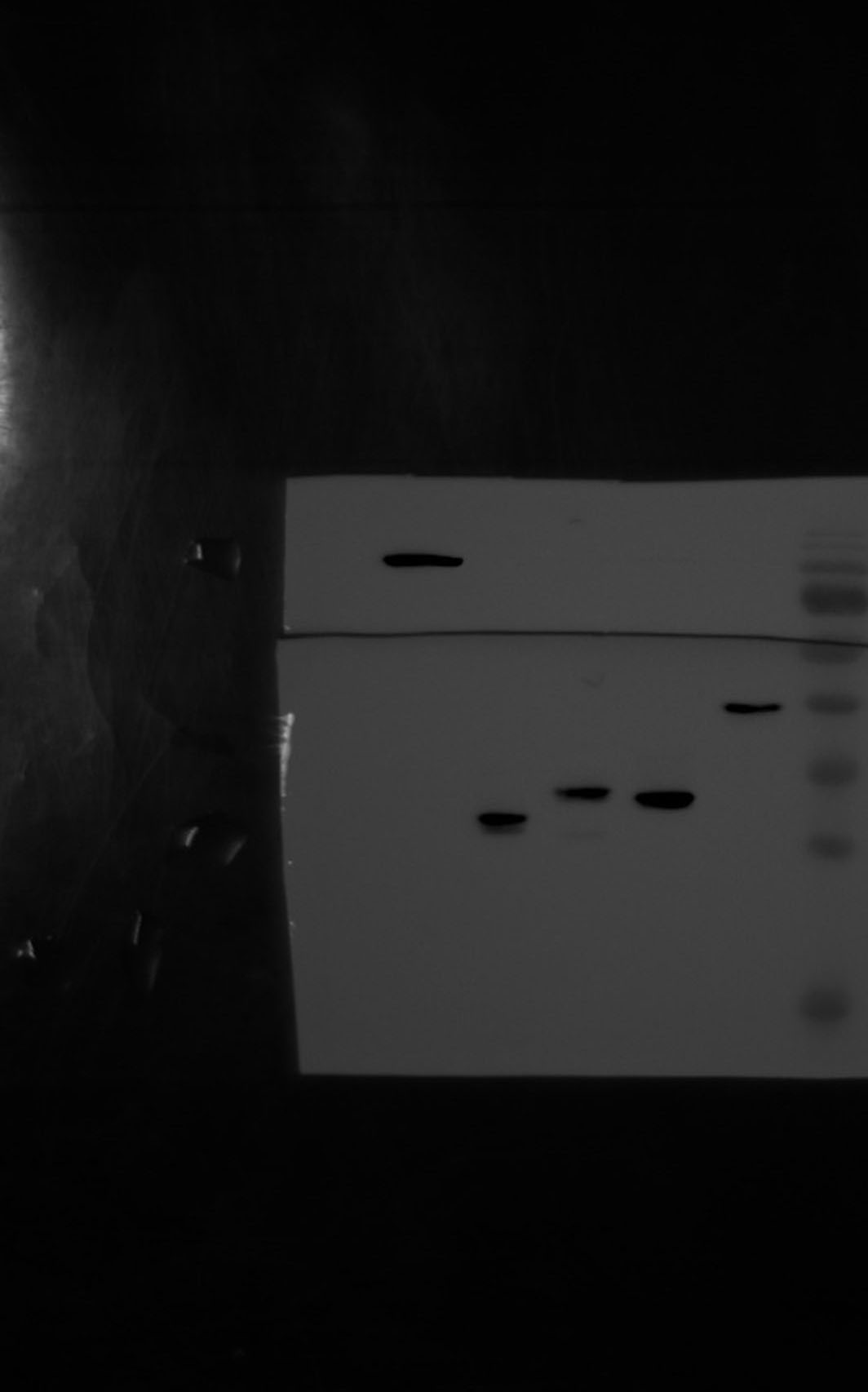

Supplement: Figure 7—source data 2. [file elife-100497-fig7-data2.zip › Figure 7-source data 2/Figure 7C-source data 2/input gfp.jpg]

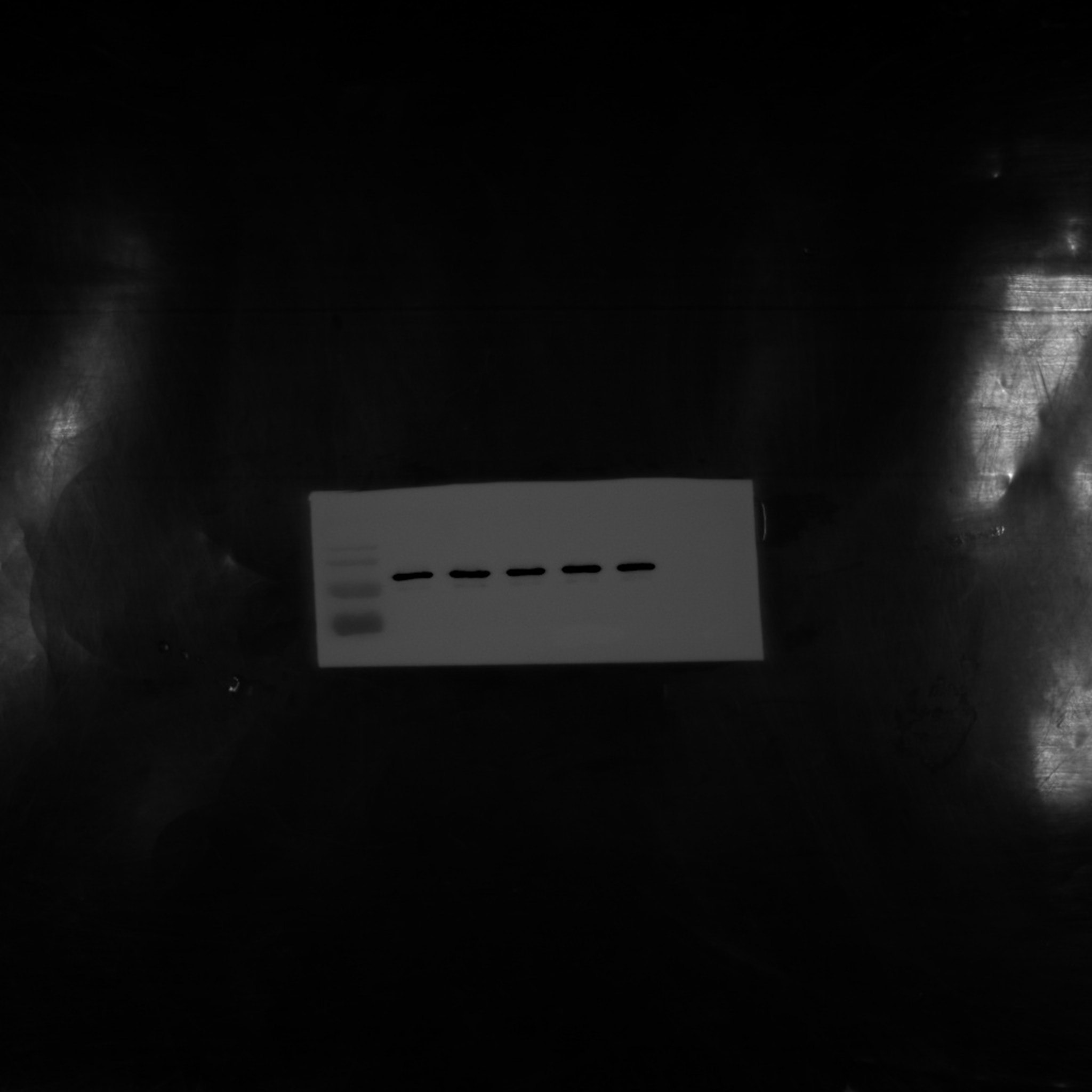

Supplement: Figure 7—source data 2. [file elife-100497-fig7-data2.zip › Figure 7-source data 2/Figure 7C-source data 2/IP F.jpg]

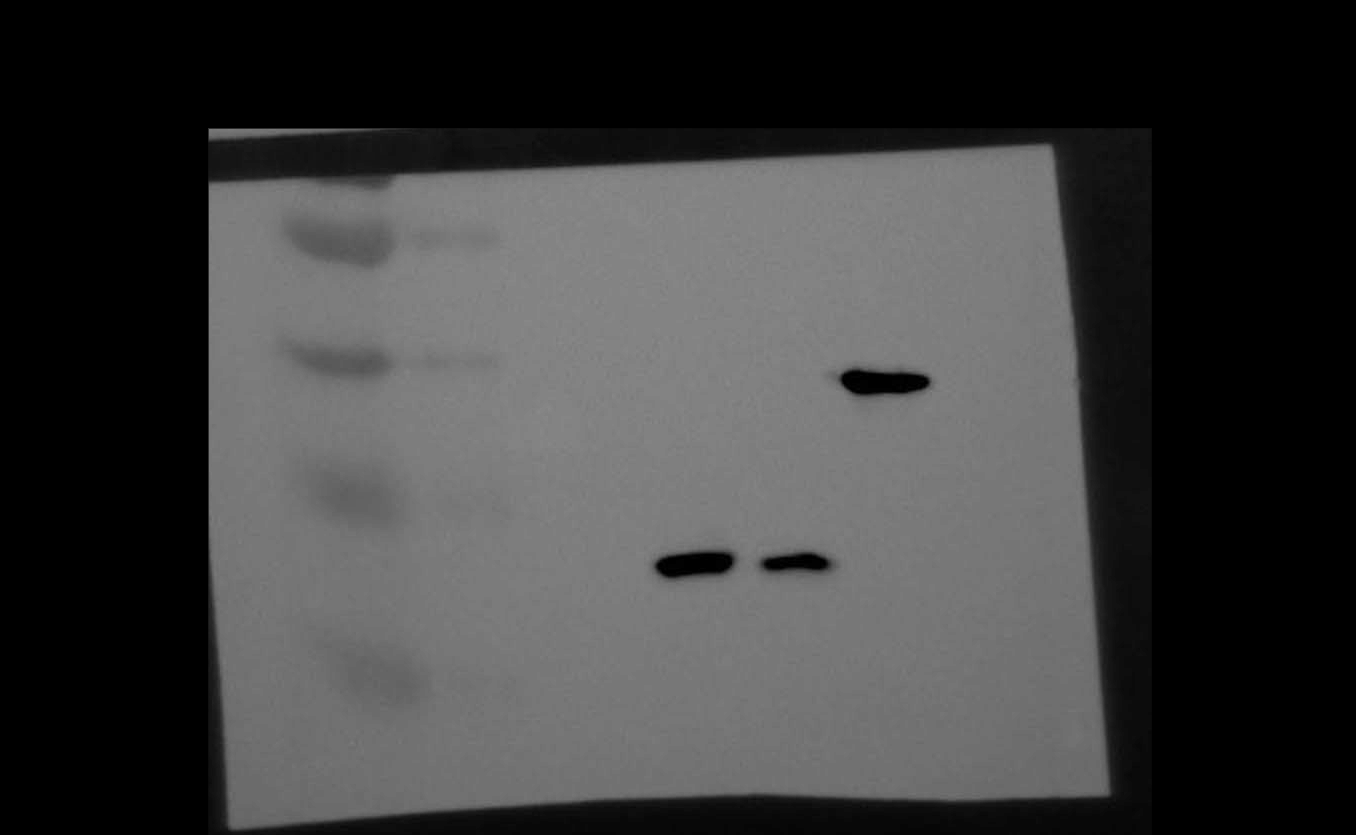

Supplement: Figure 7—source data 2. [file elife-100497-fig7-data2.zip › Figure 7-source data 2/Figure 7C-source data 2/IP GFP.tif]

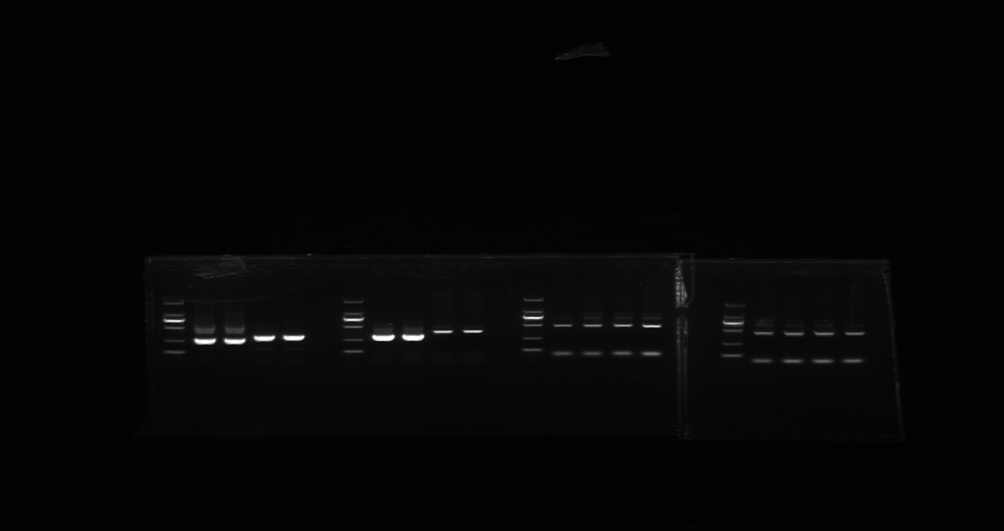

Supplement: Figure 7—figure supplement 2—source data 2. [file elife-100497-fig7-figsupp2-data2.zip › Figure 7—figure supplement 2B/alternative 3' splice site_ cirbpb cdkn2aip.tif]

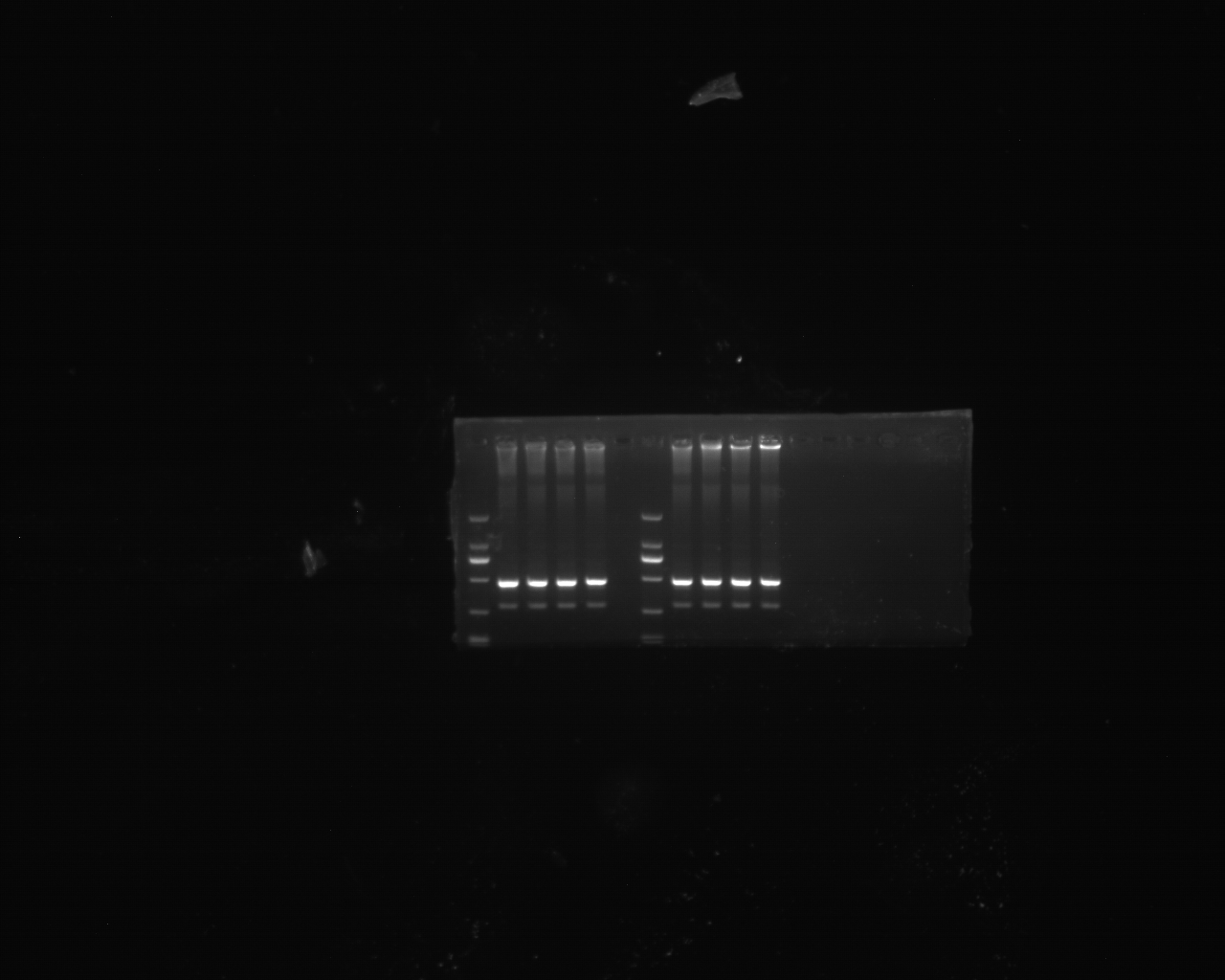

Supplement: Figure 7—figure supplement 2—source data 2. [file elife-100497-fig7-figsupp2-data2.zip › Figure 7—figure supplement 2B/alternative 3' splice site_actin.Tif]

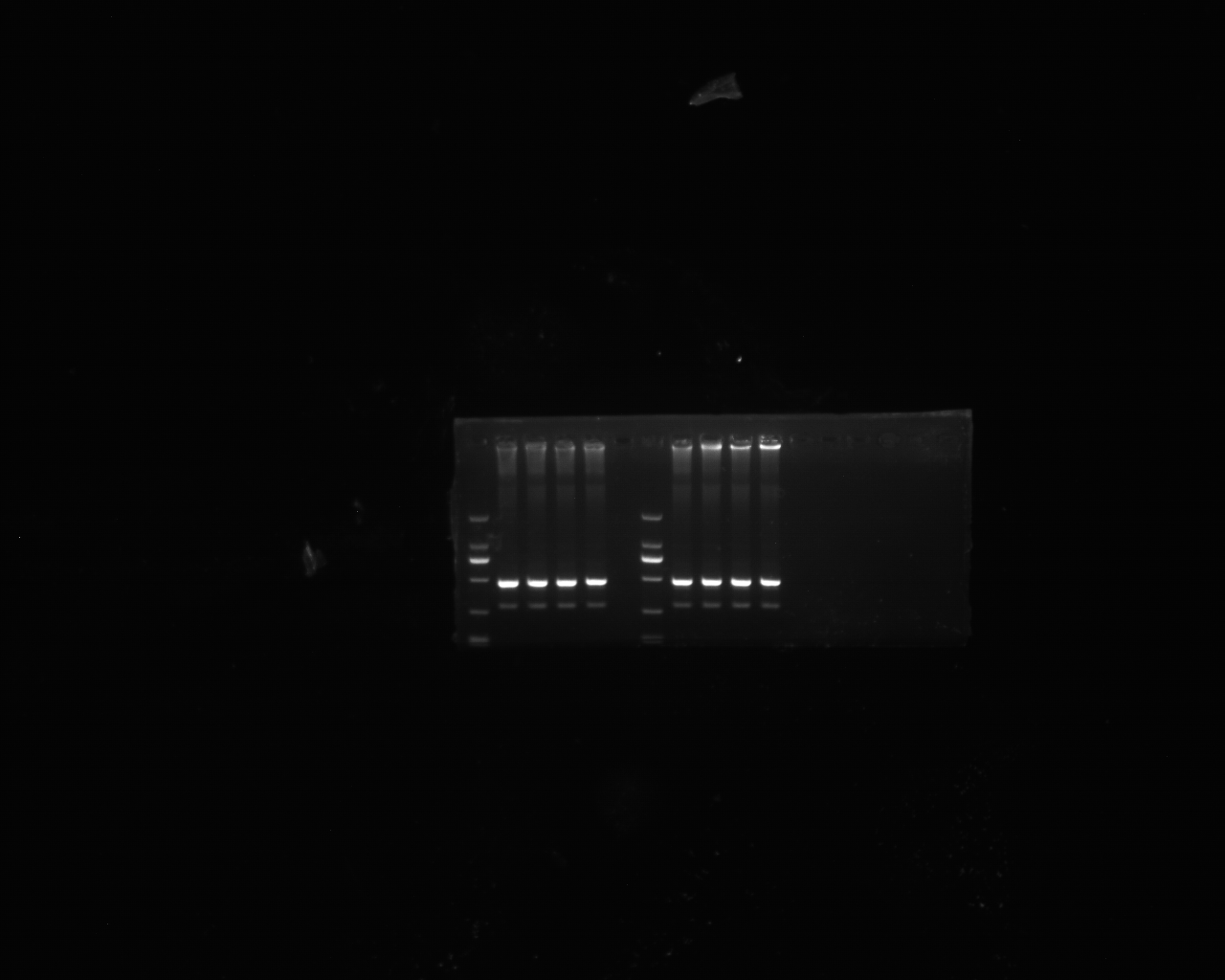

Supplement: Figure 7—figure supplement 2—source data 2. [file elife-100497-fig7-figsupp2-data2.zip › Figure 7—figure supplement 2B/alternative 5' splice site_actin.tif]

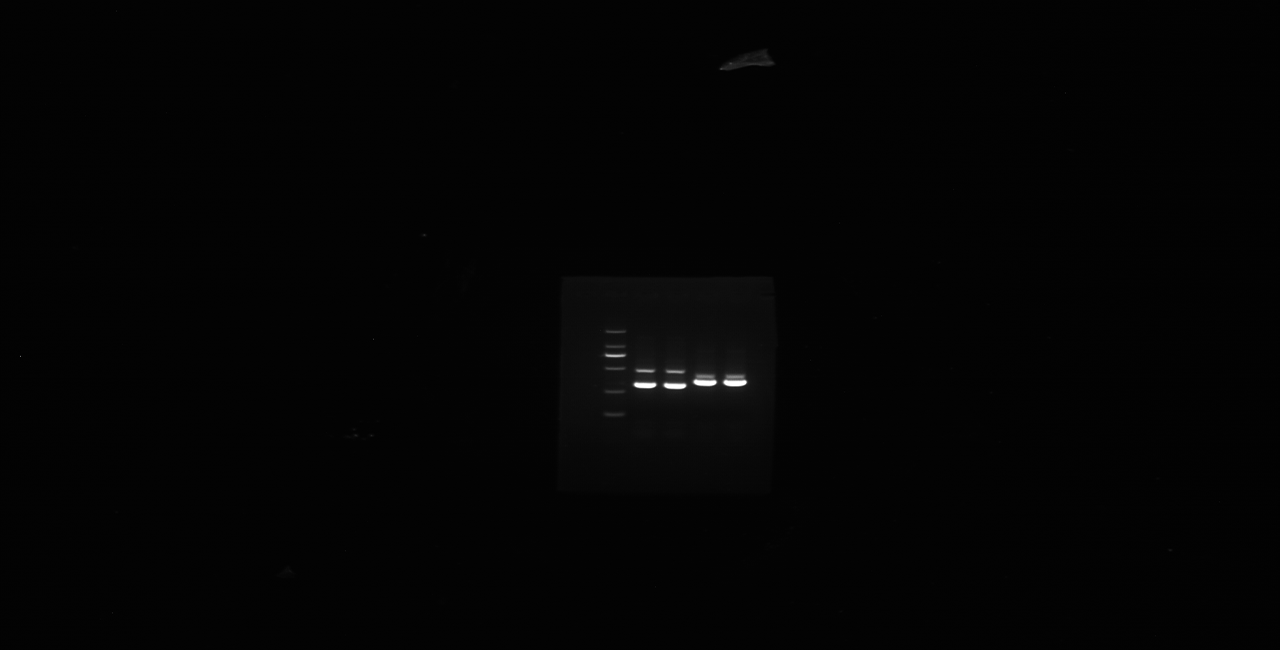

Supplement: Figure 7—figure supplement 2—source data 2. [file elife-100497-fig7-figsupp2-data2.zip › Figure 7—figure supplement 2B/alternative 5' splice site_mapk1 add1.tif]

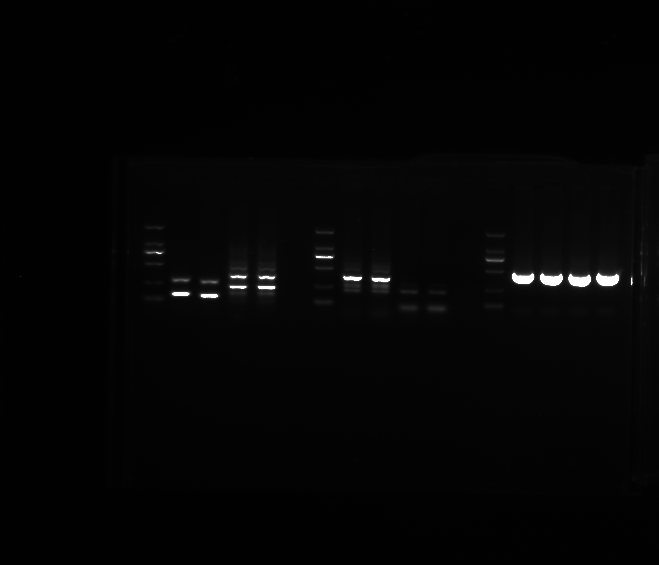

Supplement: Figure 7—figure supplement 2—source data 2. [file elife-100497-fig7-figsupp2-data2.zip › Figure 7—figure supplement 2B/Exon skipping_ubap2l cdk16 actin.tif]

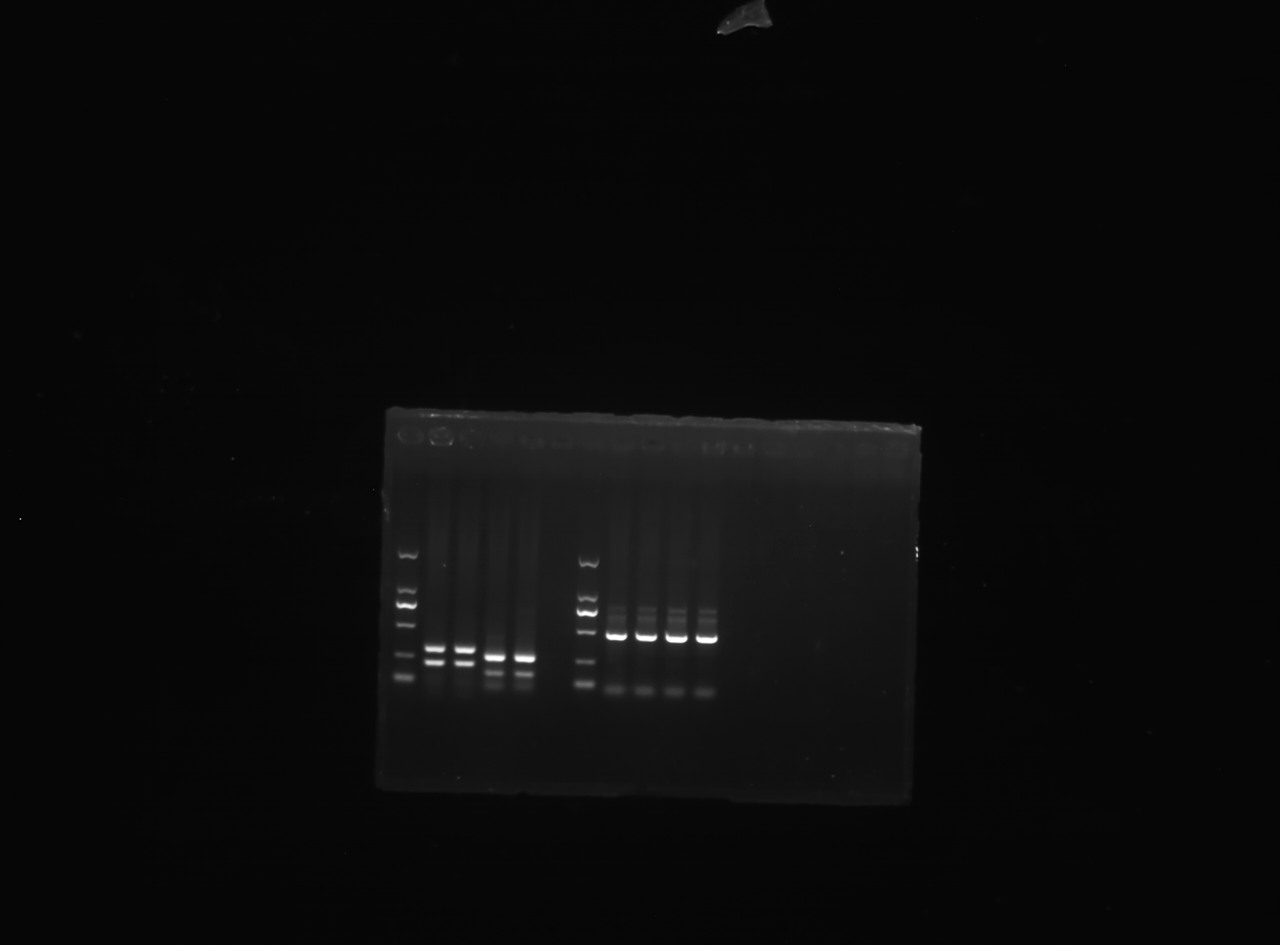

Supplement: Figure 7—figure supplement 2—source data 2. [file elife-100497-fig7-figsupp2-data2.zip › Figure 7—figure supplement 2B/Intron retention_ rbbp4 her6 actin.tif]

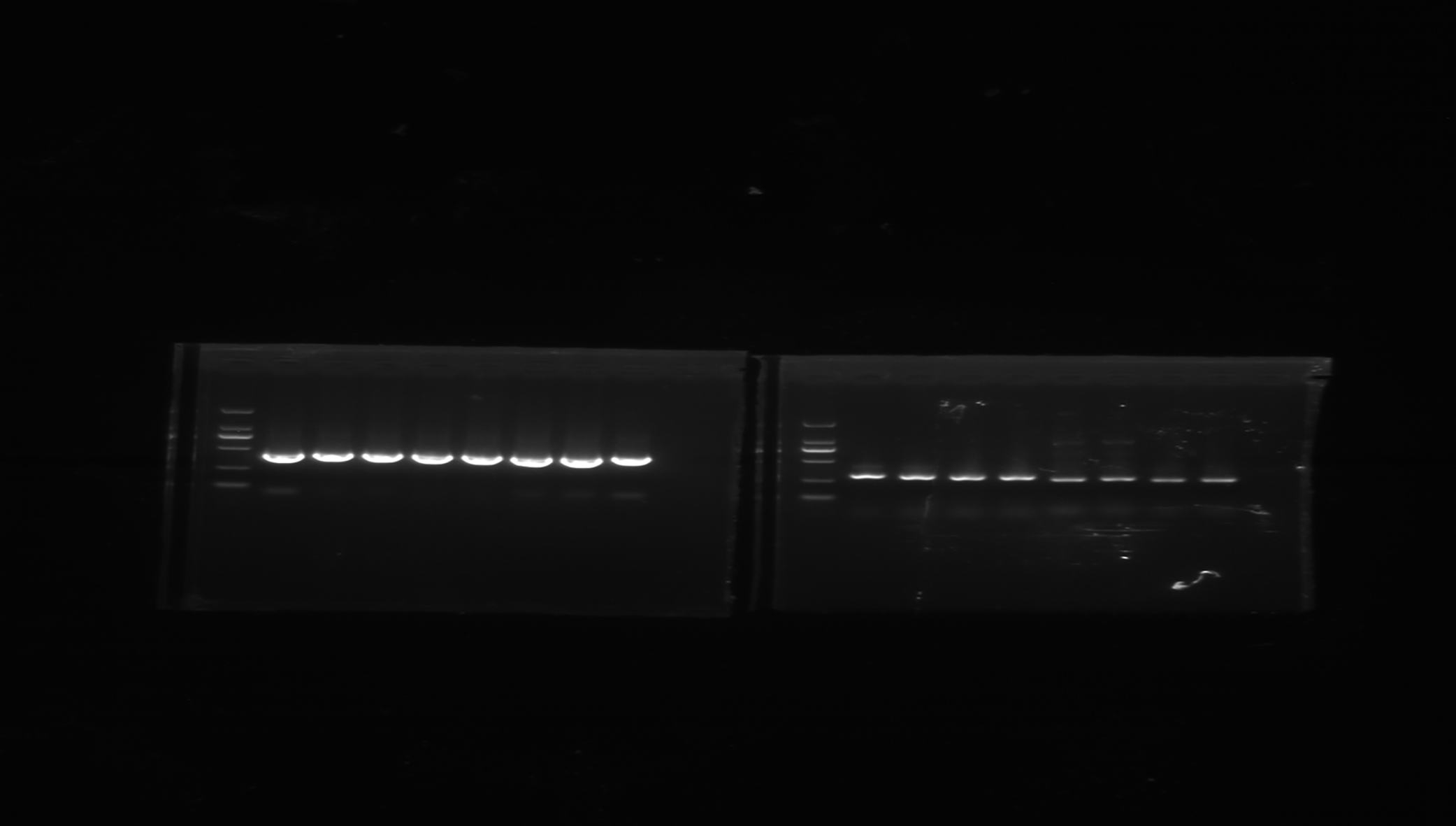

Supplement: Figure 7—figure supplement 2—source data 2. [file elife-100497-fig7-figsupp2-data2.zip › Figure 7—figure supplement 2B/mutually exclusive exons_ddb2 acox actin.tif]

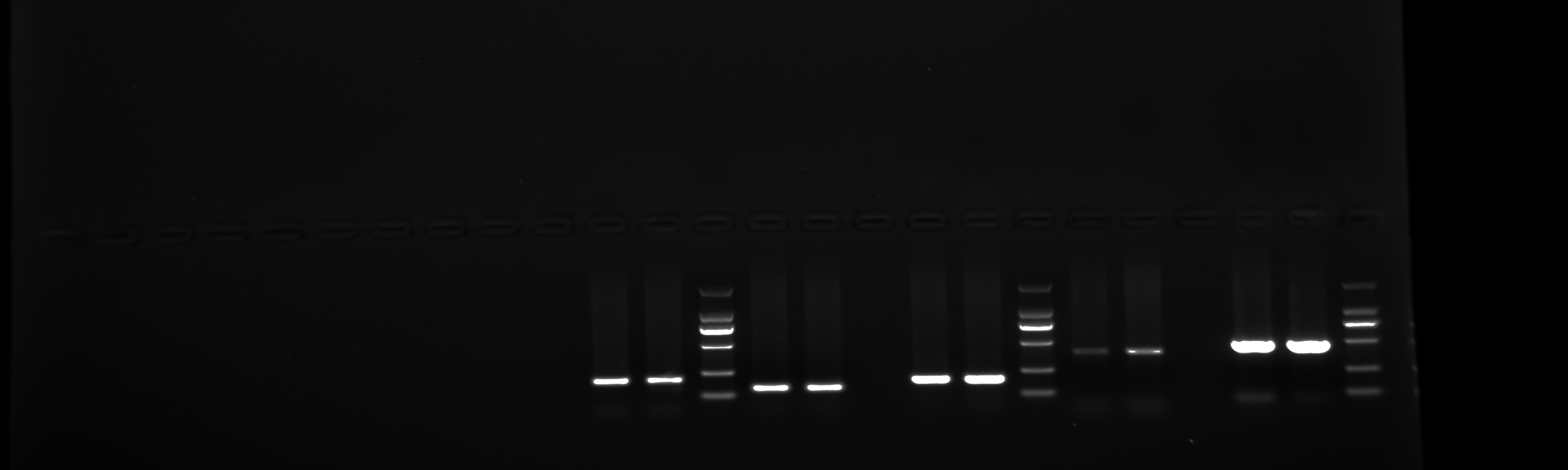

Supplement: Figure 7—figure supplement 2—source data 2. [file elife-100497-fig7-figsupp2-data2.zip › Figure 7—figure supplement 2C/mdm 4 actin.tif]

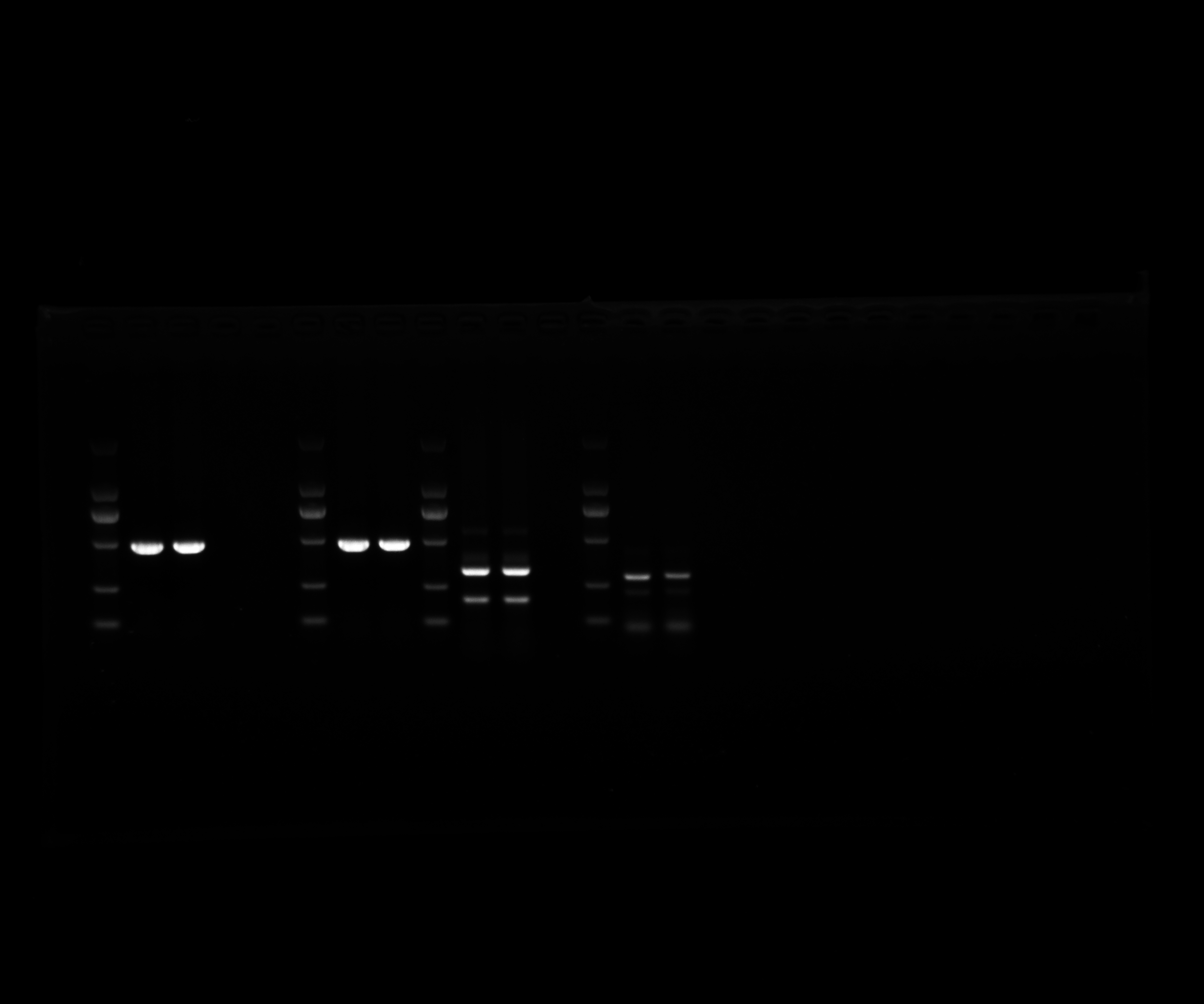

Supplement: Figure 7—figure supplement 2—source data 2. [file elife-100497-fig7-figsupp2-data2.zip › Figure 7—figure supplement 2D/B-CATENIN 2.tif]

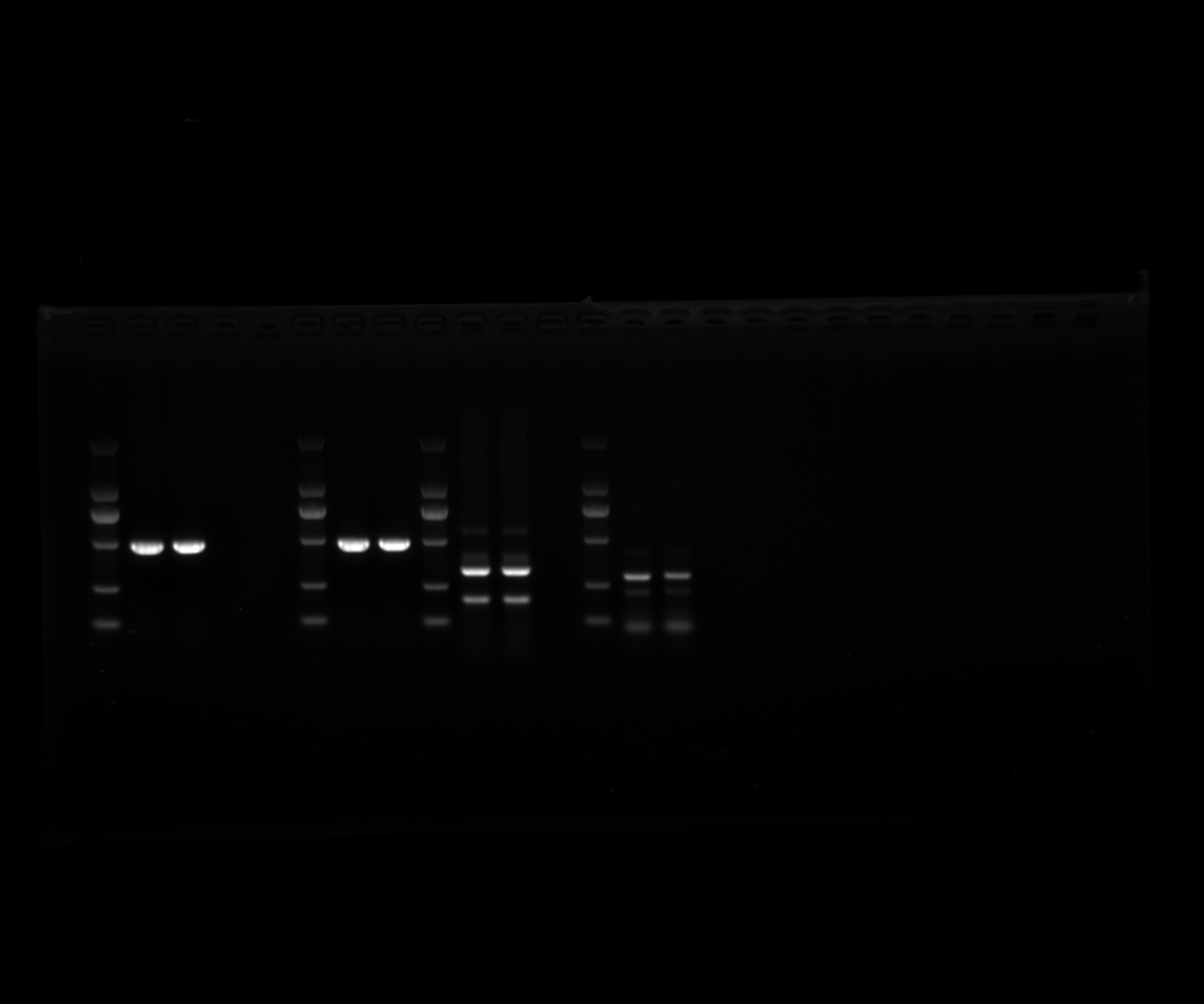

Supplement: Figure 7—figure supplement 2—source data 2. [file elife-100497-fig7-figsupp2-data2.zip › Figure 7—figure supplement 2D/b-catenin.tif]
