# Supplementary material for: BCAS2 promotes primitive hematopoiesis by sequestering β-catenin within the nucleus
Source: eLife. 2025 Jun 13;13:RP100497. doi: 10.7554/eLife.100497 (PMC12165693; doi:10.7554/eLife.100497)

Fig S14B

Uncropped gels for Supplemental Figure14B MEE

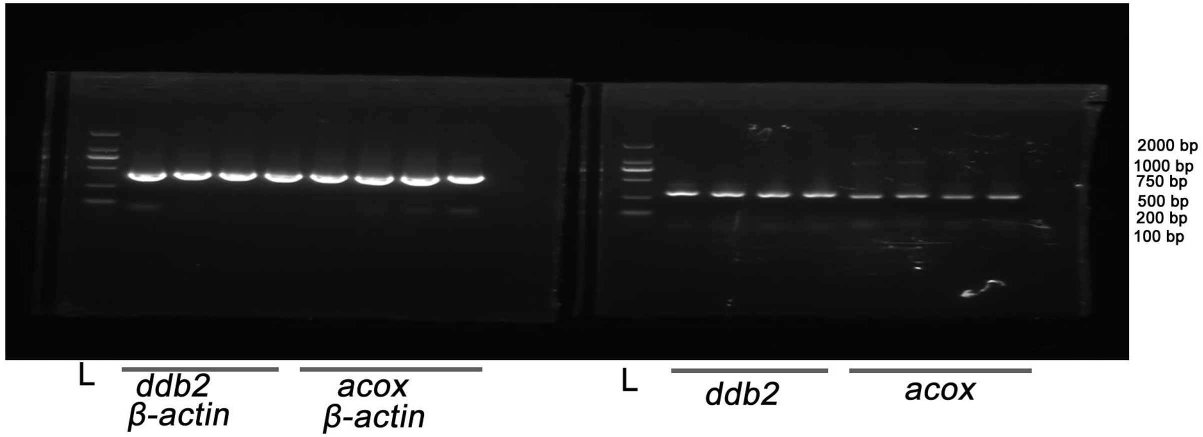

Uncropped gels for Supplemental Figure14B IR

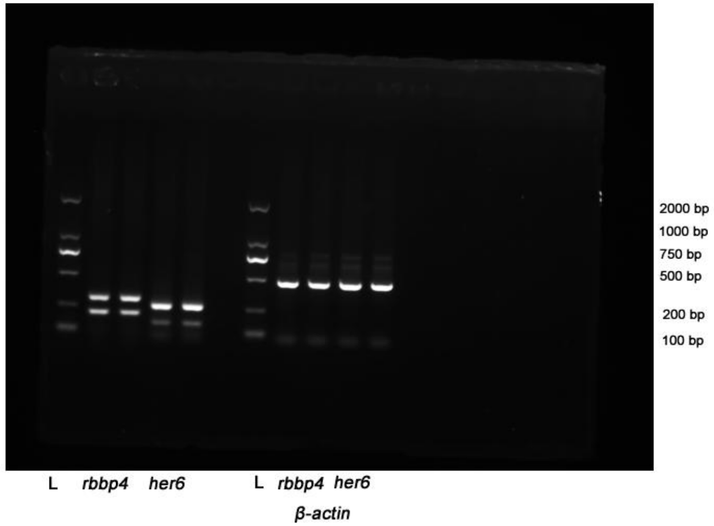

Uncropped gels for Supplemental Figure14B ES

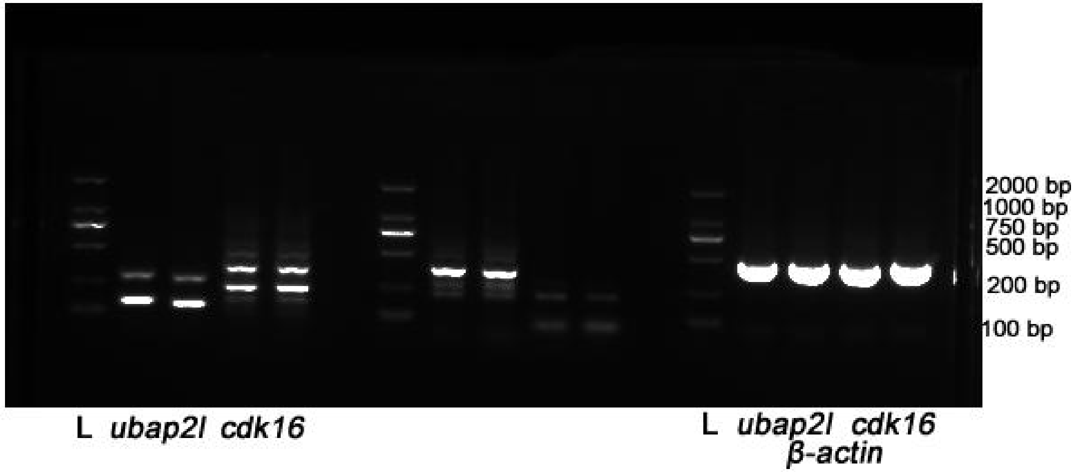

Fig S14B

Uncropped gels for Supplemental Figure14B 3’SS

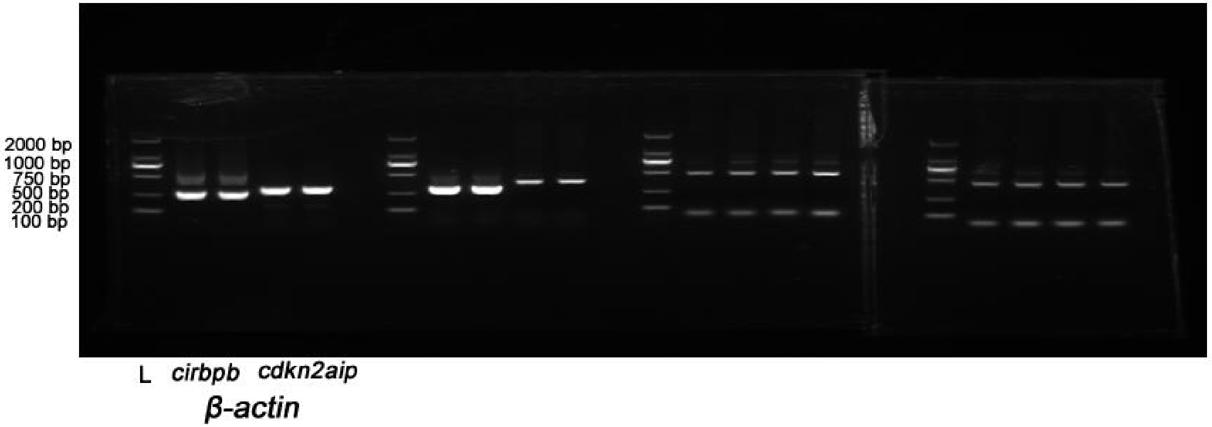

Uncropped gels for Supplemental Figure14B 3’SS

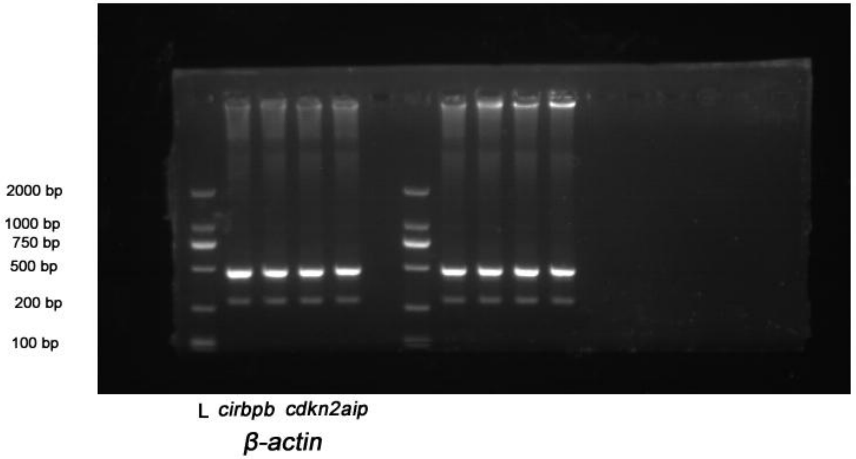

Uncropped gels for Supplemental Figure14B 5’SS

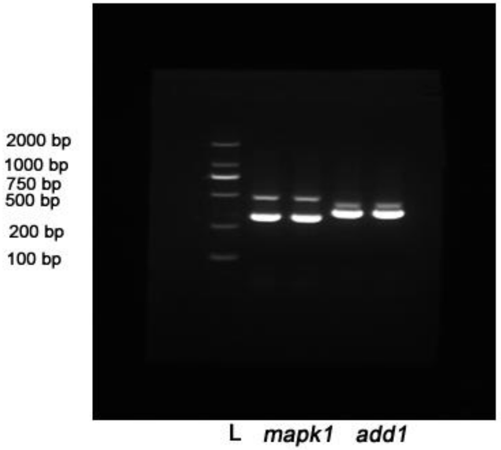

Uncropped gels for Supplemental Figure14B 5’SS

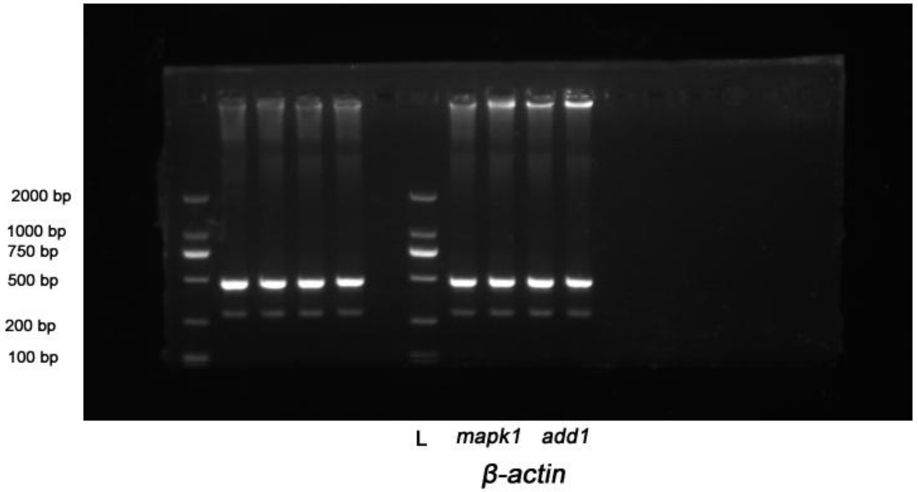

Fig S14C

Uncropped gels for Supplemental Figure14C

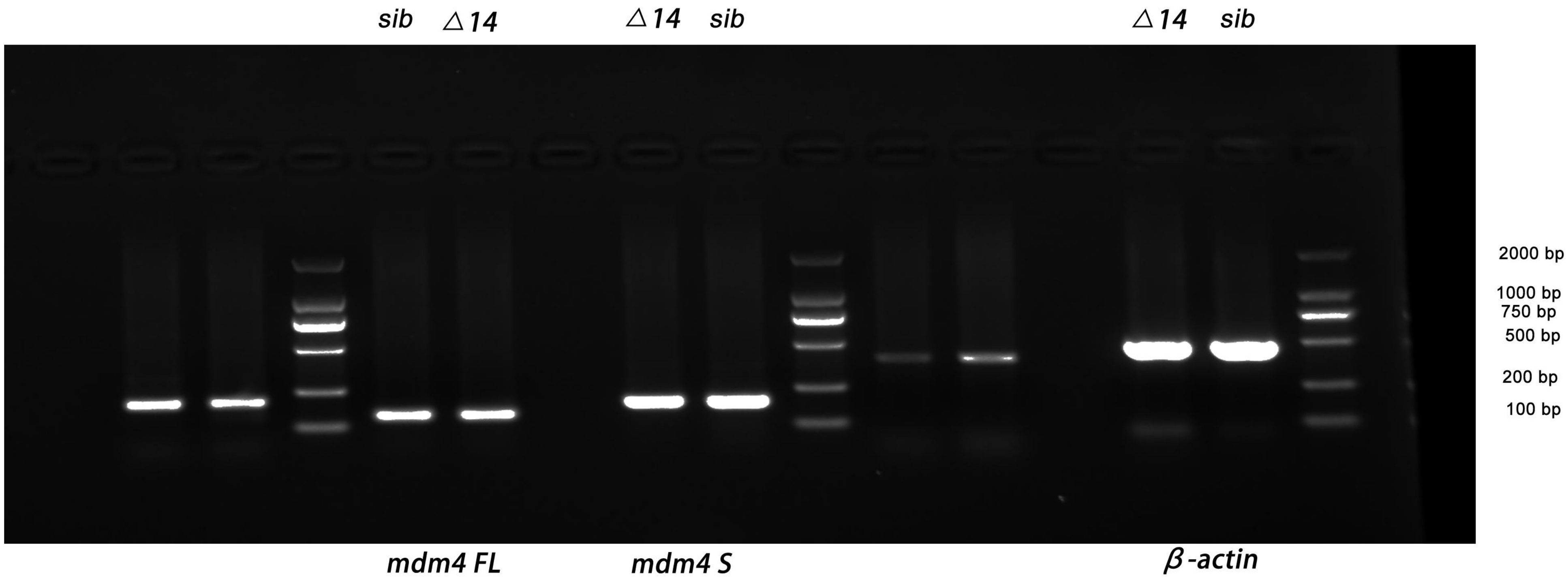

Fig S1

Uncropped gels for Supplemental Figure14D

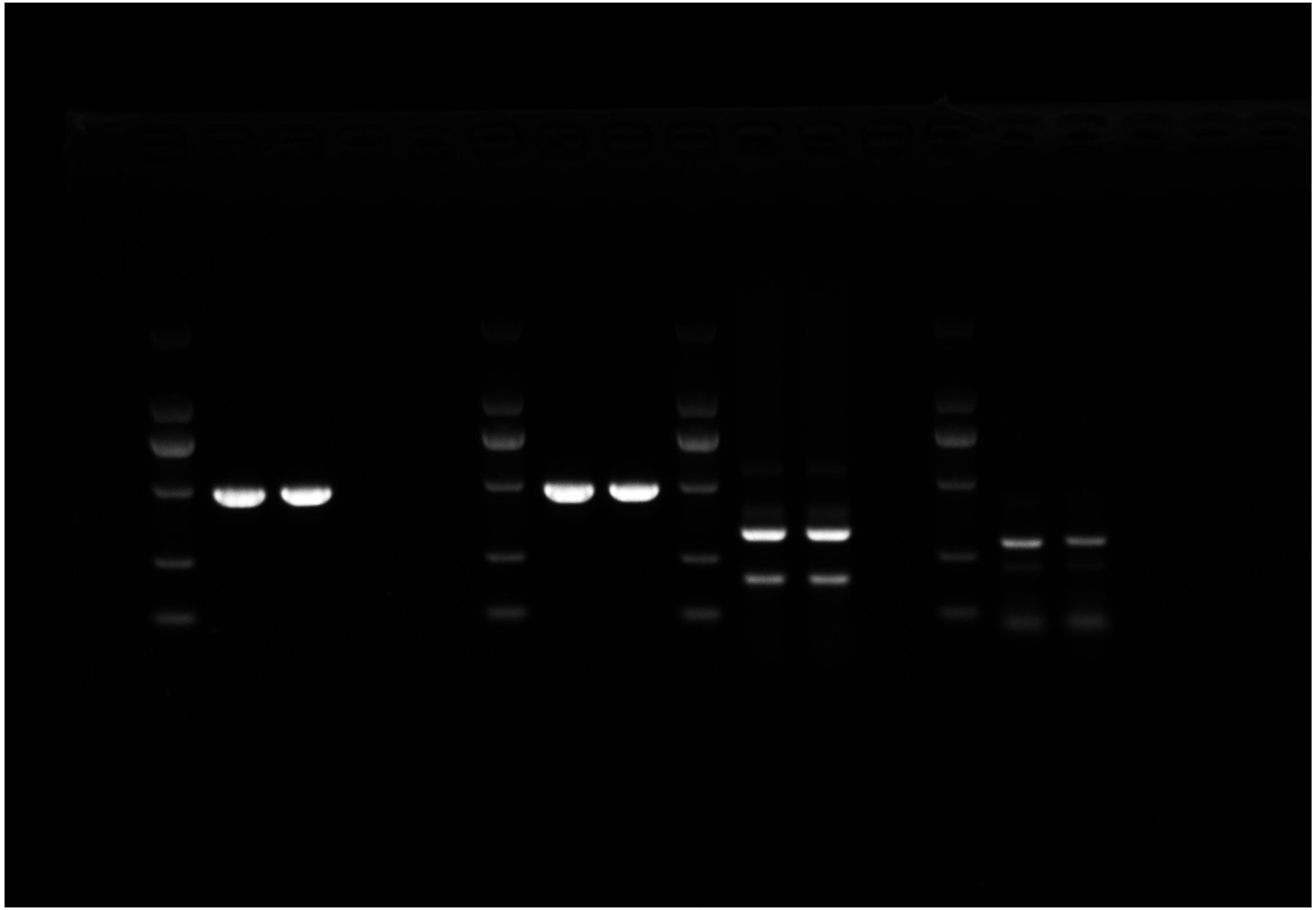

Supplement: Figure 7—figure supplement 2—source data 1. [file elife-100497-fig7-figsupp2-data1.zip › Figure 7—figure supplement 2—source data 1.pdf]
