# Supplementary material for: BCAS2 promotes primitive hematopoiesis by sequestering β-catenin within the nucleus
Source: eLife. 2025 Jun 13;13:RP100497. doi: 10.7554/eLife.100497 (PMC12165693; doi:10.7554/eLife.100497)
Supplement: Supplementary file 1. [file elife-100497-supp1.docx]

**Supplementary File 1**

**Primers Used for Genotyping**

| **Strain** | **Directions** | **Sequence (5’ to 3’)** |
| --- | --- | --- |
| *Kdr*-Cre mouse | Forward | CGGTTATTCAACTTGCACCAC |
|  | Reverse | CAGGACTGAAAGCCCAGACT |
| *Bcas2*^Flox/Flox^ mouse | Forward | ATTCCAGCAGTTGGTGTGGG |
|  | Reverse | CATTGCTGGACAGAAGGTGAG |
| *Kdr*-Cre;*Bcas2*^Flox/Flox^ mouse | Forward | AGGTGTATGAATGCCTGAACAAG |
|  | Reverse | CATTGCTGGACAGAAGGTGAG |
| *bcas2* knockout zebrafish | Forward | TGCACATACAGTAATAGGCTTACCC |
|  | Reverse | GTCTGATTTGCATCAAAAGATGTGA |
