# Supplementary material for: BCAS2 promotes primitive hematopoiesis by sequestering β-catenin within the nucleus
Source: eLife. 2025 Jun 13;13:RP100497. doi: 10.7554/eLife.100497 (PMC12165693; doi:10.7554/eLife.100497)
Supplement: Supplementary file 2. [file elife-100497-supp2.docx]

**Supplementary File 2**

**Primers Used for Reverse Transcription-PCR**

| **Symbol** | **Directions** | **Sequence (5’ to 3’)** |
| --- | --- | --- |
| *ddb2* | Forward | CGCTCACTTAAATCTTACAAGCTGC |
|  | Reverse | AGTCTGGGGTACGAGACAATATCT |
|  | Reverse | TTGGCAAAGACTTGTATAACGGATC |
| *acox* | Forward | ATCCAGATTTCCAACATGAAGACCT |
|  | Reverse | AGGTGCCTAACATATGGAATGACTC |
|  | Forward | TTCTCTCTCGAAGTGAGCGATATGA |
|  | Reverse | TCTGTGCGTAGGTGCCAATAATCTC |
| *ubap2l* | Forward | GAATGTTGGTGTCAATGCATCAGCT |
|  | Reverse | CTGCGTCTTTGTGTAAACAGAGCCT |
| *cdk16* | Forward | ATGAGGAAGATCAAACGGCAGCTGT |
|  | Reverse | AAATTTTGCGACCGGGGTTGTTTCT |
| *rbbp4* | Forward | TGGGACACTCGTTCCAATAACACAT |
|  | Reverse | CTGGAAGATTTCATCTTTGTGCGAC |
| *her6* | Forward | GGAGAAAAGAAGAAGAGCGAGAATC |
|  | Reverse | GCTGCATGTTTCTGAGATGTTTCAC |
| *cirbpb* | Forward | TCCTACAGAGACGGTTACGACAGTT |
|  | Reverse | CCTCACCAGGCTTGAAACTCTTACA |
| *cdkn2aip* | Forward | TCGCTAATGAGGACTTGTCTTTGGA |
|  | Reverse | ATTTATCTTGTCCATCACACGCTGC |
| *mapk1* | Forward | GTCTGTTGGTTGTATTCTGGCTGAG |
|  | Reverse | AGGATCATAGTACTGCTCCAGGTAC |
| *add1* | Forward | AGGATGCTGGATAATCTGGGCTACA |
|  | Reverse | GTTGCGCATCTCTTGGACTTCTTTC |
| *Mdm4-*FL | Forward | GGTCAGGTGTCCAGTGAGTCAATAA |
|  | Reverse | AAACCATCTGAGGAGTCTTCATCTG |
| *Mdm4-*S | Forward | GAAGATCCTGGTCAGACTCCTCAGA |
|  | Reverse | CGGGAGAGAGTTGATTGGTGTGAAT |
| *β-catenin* | Forward | GATGACGATGTGGATAATCAGGTGC |
|  | Reverse | GAACTGTGTGGAAGGTATCTGCATG |
|  | Forward | GGAGACAATTTCTCATTATTCTGGC |
|  | Reverse | TCAGTAGTTTGGTGAGCTCTGGAAT |
| *β-actin* | Forward | ATGGATGATGAAATTGCCGCAC |
|  | Reverse | ACCATCACCAGAGTCCATCACG |
